# Occupational health and safety regulatory interventions to improve the work environment: An evidence and gap map of effectiveness studies

**DOI:** 10.1002/cl2.1371

**Published:** 2023-12-11

**Authors:** Anja Bondebjerg, Trine Filges, Jan Hyld Pejtersen, Malene Wallach Kildemoes, Hermann Burr, Peter Hasle, Emile Tompa, Elizabeth Bengtsen

**Affiliations:** ^1^ VIVE—The Danish Center for Social Science Research Copenhagen Denmark; ^2^ Federal Institute for Occupational Safety and Health (BAuA) Berlin Germany; ^3^ University of Southern Denmark Odense Denmark; ^4^ Institute for Work and Health Toronto Canada

## Abstract

**Background:**

Unsafe and unhealthy working conditions lead to injuries and financial losses across the globe, resulting in a need for research into effective work environment interventions.

**Objectives:**

The objective of this evidence and gap map (EGM) is to provide an overview of existing systematic reviews and primary studies examining the effects of occupational health and safety regulatory interventions.

**Search Methods:**

Relevant studies are identified through searches in published and unpublished literature performed up to January 2023.

**Selection Criteria:**

The population for this EGM is workers above the age of 15 and their workplaces within the OECD. We include randomised controlled trials, non‐randomised studies with a comparison of two or more groups of participants, and systematic reviews of effects.

**Data Collection and Analysis:**

The map has been populated based on information about interventions and outcomes, study design, OECD country, and publication status. We have performed critical appraisal of included systematic reviews using an adjusted version of the AMSTAR‐2 tool.

**Main Results:**

The included studies for this report consist of six systematic reviews, 28 primary effect studies, and three on‐going studies. The interactive map shows that the largest cluster of studies is located in the inspection activity domain, while the sickness absence outcome domain and the intervention categories for training initiatives and formulation of regulatory standards are only scarcely populated. Additionally, the AMSTAR‐appraisal suggests a lack of rigorous systematic reviews and meta‐analyses.

**Authors’ Conclusions:**

More research in the form of primary studies and rigorous systematic reviews is needed to provide stakeholders with better guidance as to what constitutes the most efficient regulatory approaches to improve the work environment.

## 1 PLAIN LANGUAGE SUMMARY

### 1.1 Mapping the evidence on occupational health and safety regulatory interventions reveals that more research is needed

Evidence for the effects of occupational health and safety regulatory interventions is unevenly distributed across intervention domains, pointing to both evidence clusters suited for further systematic review activity and evidence gaps where more primary research is needed.

#### 1.1.1 What is this evidence and gap map (EGM) about?

Across the globe, unsafe and unhealthy working conditions constitute a serious public health risk. It is therefore imperative that research about effective occupational health and safety regulatory interventions be made available to decisionmakers.

This map displays existing systematic reviews and primary studies investigating the effects of interventions aimed at introducing, monitoring, and ensuring compliance with occupational health and safety regulations.

**What is the aim of this EGM?**

The aim of this EGM is to present available evidence from systematic reviews and primary studies examining the effects of occupational health and safety regulatory interventions on a set of outcomes encompassing both organisational/workplace level domains (e.g. compliance with regulatory standards) and individual/worker level domains (e.g. work‐related injuries).

#### 1.1.2 What studies are included?

The EGM includes six systematic reviews, 28 effect studies, and three on‐going studies. Included studies had to be either randomised controlled trials, non‐randomised studies with a comparison of two or more groups of participants, or systematic reviews of effects.

Furthermore, the population of interest was restricted to workers above the age of 15 employed at workplaces within the OECD.

#### 1.1.3 What are the main findings of this EGM?

Evidence for the effects of health and safety regulatory interventions is unevenly distributed across intervention domains. The largest cluster of studies is found in the intervention domain covering inspection activity (21 studies).

There are a number of studies investigating information, guidance and consulting activities (12 studies), incentives for compliance (seven studies), and regulatory enforcement in the form of sanctions (five studies).

There are very few studies investigating the effects of training initiatives (two studies) and formulating regulatory standards (one study).

Included systematic reviews were critically appraised. This appraisal led to the conclusion that there is a lack of rigorously‐performed systematic reviews exploring the effects of occupational health and safety regulatory interventions.

In terms of the geographical spread of studies across the OECD area, North America (USA and Canada) is the most densely populated, followed by Europe (represented by countries located in both the Northern and Southern parts of the continent), and finally Eastern Asia (represented by South Korea).

#### 1.1.4 What do the findings of this EGM mean?

This EGM provides an opportunity for stakeholders from the research, policy and practice communities to gain an overview of the available evidence on the effects of occupational health and safety regulatory interventions.

By pointing to both evidence clusters and gaps in the current evidence base, the EGM can inspire future research projects and intervention activities.

#### 1.1.5 How up‐to‐date is this EGM?

The authors carried out literature searches up to January 2023.

## 2 BACKGROUND

### 2.1 Introduction

#### 2.1.1 The problem, condition or issue

Violations of occupational health and safety regulatory standards pose significant regulatory challenges to lawmakers and working environment authorities on a global scale. Around the world, unsafe and unhealthy working conditions, including, for example, exposure to hazardous materials, use of dangerous equipment, and adverse psychosocial working environments, lead to both human and financial losses that put a strain on labour markets and public welfare institutions. The International Labour Organisation (ILO) estimates that 2.78 million workers die each year from occupational accidents and diseases, with an additional 374 million workers experiencing non‐fatal occupational injuries or illnesses (The International Labour Organization, 2020). In financial terms, occupational accidents and diseases are estimated to cause an annual 4% loss of global GDP (The International Labour Organization, 2020).

With the adverse effects of contraventions of occupational health and safety regulatory standards being indisputable, there is a need to ensure that research about what constitutes effective occupational health and safety regulatory interventions is available to policymakers, regulatory agencies, and other key stakeholders. Several studies have reviewed the literature on the effects of regulatory enforcement tools such as workplace inspections, consultative activities, and awareness campaigns. Tompa (2007) performed a systematic review of insurance and regulatory mechanisms, which was followed up by two reviews published in 2016, focusing on the quantitative and the qualitative regulatory enforcement literature, respectively (MacEachen, 2016; Tompa, 2016). Findings from the 2007‐review found evidence that first hand experience of citations and penalties (which can be defined as specific deterrence) reduced injuries, whereas there was limited evidence for the effects of first hand experience of inspection. Similarly, there was limited evidence for the probability of inspections, citations, and penalties (defined as general deterrence) leading to reduced injuries (Tompa, 2007). The quantitative review from 2016 also found evidence to suggest that several regulatory enforcement mechanisms had an effect on injuries and compliance with regulation. Particularly, there was a specific deterrence effect from inspections with penalties which resulted in a decrease in injuries, similar to findings from the 2007‐review (Tompa, 2016). Results from a Cochrane intervention review by Mischke (2013) were also in line with the findings from the above mentioned reviews, in that evidence was found for inspections leading to decreases in injuries, with indications of specific, focused inspections having potentially larger effects than inspections in general. However, this review also pointed to a lack of high‐quality evidence and an urgent need for better designed evaluations to establish the effects of enforcement methods (Mischke, 2013). Finally, a systematic review by Andersen (2019) indicated that legislative and regulatory policy may reduce injuries and fatalities and improve compliance with regulation, but major research gaps were identified concerning the effects of occupational health and safety regulation targeting psychological and musculoskeletal disorders.

As can be seen from the above presentation of previous reviews, there is evidence to suggest that regulatory interventions may serve as effective tools for governments tasked with protecting workers from health and safety risks at workplaces. At the same time, it is clear that there are knowledge gaps in the literature which make it hard to determine relationships of cause and effect pertaining to particular intervention and outcome domains. In addition, there is a multitude of different regulatory interventions which may lead to diverse effects on a large number of outcomes, ranging from intermediate outcomes on the organisational level (such as compliance with regulations and workplace exposures) to final outcomes for individual workers (such as the prevalence of ill‐health and injuries). Finally, occupational health and safety regulation as a research field is characterised by methodological diversity, with researchers drawing on various types of both quantitative and qualitative methods to gain insight into the complexities of regulatory interventions. Taken together, the existence of knowledge gaps and the extent and diversity of the research literature can make it challenging for both government stakeholders, regulatory agencies, and researchers alike to gain an overview of what evidence is available for particular interventions. This points to the need for applying methods of knowledge mapping that are suited to the task of creating overviews of large amounts of data in a format that is accessible to various stakeholders. One such method is the evidence and gap map (EGM) approach which we apply in this report.

As stated by Saran (2018) in their overview of approaches to evidence mapping, evidence maps constitute a relatively new approach to systematically reporting research activity for broader topic areas than those made possible by traditional, focused research syntheses, such as systematic reviews. Evidence maps are well suited for guiding stakeholders to high quality research, identifying research gaps, and pointing out the direction for more focused research syntheses (Saran, 2018). The current EGM intends to do just that: to provide an easily accessible, visual representation of the availability of evidence for the effects of a number of occupational health and safety regulatory interventions on a predefined set of workplace and worker level outcomes. In this study, we adopt a functional definition of regulation in which regulation involves: (a) setting regulatory standards, (b) monitoring compliance through inspections, and (c) enforcing regulatory standards (incentives and sanctions). This functional definition is reflected in our intervention framework which covers these three regulatory functions, as well as information and training initiatives initiated by regulatory agencies to improve compliance with regulatory standards. As mentioned, enforcement of working environment regulation can influence a host of outcomes, including both intermediate workplace outcomes and individual worker outcomes. Therefore, this EGM includes a number of outcome domains intended to cover both the intermediate and final outcome levels.

As noted, research in occupational safety and health regulation is characterised by methodological diversity, with strong traditions of both quantitative and qualitative enquiry. However, we do not attempt to cover the occupational health and safety regulatory enforcement literature in its entirety. Our aim with this EGM lies specifically in finding studies designed to make causal inferences about intervention effects. Therefore, we limit our scope to a subset of studies within the larger occupational health and safety regulation literature, that is, primary studies of effectiveness and systematic reviews of effects. This choice has been guided by the input we have received from our stakeholders within The Danish Working Environment Authority, who have expressed a need for a better overview of existing research and a clearer picture of potential research gaps and avenues for future research. A possible next step following this EGM is to perform a full systematic review and meta‐analysis for an intervention and outcome combination covered by sufficient evidence. As noted by Snilstveit (2016), identifying areas or ‘synthesis gaps’ where systematic reviews can be of particular value are one of the key potentials of EGMs and a fruitful avenue for making research evidence available and useful to stakeholders.

#### 2.1.2 The intervention

As noted, the intervention framework reflects the proposed definition of regulation as involving three main functions: setting regulatory standards, monitoring compliance through inspections, and enforcing regulatory standards (incentives and sanctions). In addition to this, we are interested in other efforts made by regulatory agencies to improve compliance or deter non‐compliance, specifically information and training initiatives. In accordance with this, we focus on six types of occupational health and safety regulatory initiatives: (1) formulation of regulatory standards, (2) incentives for compliance, (3) inspection by regulatory agencies, (4) enforcement by regulatory agencies (sanctions), (5) information, guidance, and consulting, and finally (6) training initiatives. We provide further details on the intervention categories in Table 1. Our outcomes of interest include both workplace and worker level categories, with examples being compliance with occupational health and safety regulatory standards, exposure to harmful substances, and incidence of work‐related injuries and ill‐health.

**Table 1:** Intervention categories.

Formulation of regulatory standards	Introduction of occupational safety and health regulatory standards or legislation.
Incentives for compliance	Incentives introduced by regulatory agencies to facilitate compliance with regulatory standards, including financial incentives such as funding or subsidy programmes as well as non‐financial incentives in the form of, e.g., recognition schemes or occupational health and safety certification.
Inspection by regulatory agencies	Inspections of all types carried out by regulatory agencies.
Enforcement by regulatory agencies (sanctions)	Sanctions imposed due to lack of compliance with regulatory standards, including, e.g., fines, enforceable undertakings, and orders to comply.
Information, guidance, and consulting activity	Information, guidance, and consulting activities initiated by regulatory agencies, e.g., targeting specific types of workplaces or particular workplace hazards (such as falls in the construction industry).
Training initiatives	Initiatives taken by regulatory agencies to train or educate workplaces, workers, managers, and other OHS professionals in, e.g., safety management or use of mechanical aids or protective gear.

#### 2.1.3 Why it is important to develop the EGM

EGMs are a valuable tool in helping researchers and decision makers make sense of the evidence available, supporting the creation of evidence‐informed policies and guiding research prioritisation. We intend this EGM to be of use to both researchers, decision‐makers, and practitioners working in the field of occupational health and safety regulation. To ensure that our work is relevant to stakeholders, we have taken guidance from key persons within The Danish Working Environment Authority. Furthermore, we have included a group of international researchers with content knowledge expertise as co‐authors of the EGM.

To our knowledge, no EGMs exist that assess the available evidence on the effects of occupational health and safety regulatory interventions. The EGM approach can be instrumental in a number of ways, for example, by guiding decision‐makers and other stakeholders to areas covered by sufficient evidence, identifying areas with a sufficient number of studies to merit a systematic review and meta‐analysis, and enabling targeted commissioning of new research in areas characterised by knowledge gaps (Snilstveit, 2016).

## 3 OBJECTIVES

The current EGM presents available systematic reviews and primary studies examining the effectiveness of interventions aimed at the setting/introduction, monitoring, and/or compliance with occupational health and safety regulations. The three overarching objectives of the map are to:

1. Provide an overview of the existing evidence base by identifying available systematic reviews and primary effect studies,
2. Identify clusters of evidence suitable for a systematic review,
3. Identify gaps in evidence where primary research is needed.

## 4 METHODS

In the published protocol (Bondebjerg, 2022), we provided descriptions of the EGM framework and the choices made concerning study selection, methodology, and analytical procedures. In the following sections, we will reiterate these choices and their justifications.

### 4.1 EGM: Definition and purpose

This EGM provides a visual overview of the available evidence from primary quantitative effect studies and systematic reviews on the effects of a set of occupational health and safety regulatory interventions. In this sense, the EGM identifies what quantitative empirical knowledge is available about the effects of these interventions by mapping available systematic reviews and primary studies of effectiveness and placing them in a graphical matrix displaying areas characterised by strong, weak, or nonexistent evidence. It is important to note here that an EGM shows the availability of data, but does not synthesise the data. As expressed by Saran (2018) in their paper on approaches to EGMs: ‘Evidence maps summarize what evidence there is, not what the evidence says’ (Saran, 2018, p. 11).

The present EGM consists of two primary dimensions: rows listing interventions, and columns listing outcomes, meaning that each cell shows studies containing evidence on that particular combination of intervention and outcome. The map also contains relevant filters (study design including AMSTAR‐2, OECD country, and publication status) making it possible to focus on a subset of studies meeting certain criteria. The map has been populated based on:

- Criteria for inclusion and exclusion of studies.
- Types of studies to be included.
- Quality appraisals of systematic reviews using AMSTAR‐2 (Assessing Methodological Quality of Systematic Reviews [Shea, 2017]). Please note that we used the AMSTAR‐2 tool with three additions; see ‘Tools for assessing risk of bias/study quality of included reviews’.

### 4.2 Framework development and scope

We have followed the standard EGM framework as a matrix, with rows containing intervention domains and columns containing outcome categories. Our framework of specific interventions and outcomes, as described in the following, has been developed in cooperation with stakeholders from The Danish Working Environment Authority. It has been our goal to build a framework of relevance, not only to other researchers, but also to decision makers and practitioners who are tasked with developing regulatory standards, performing inspections and enforcing compliance, and taking other measures to improve working environment conditions.

### 4.3 Stakeholder engagement

Engaging primary stakeholders in an EGM can play a central role in defining the scope of the investigation, developing a coherent framework, and making sure that the questions asked are the most relevant and pertinent to the research subject. As noted by Keown (2008), a systematic review (or in this case, an EGM) provides a number of engagement opportunities in various phases of the review process, from cooperating with stakeholders on finding a topic of mutual relevance, to arranging on‐going feedback meetings, and involving stakeholders in the dissemination of the review or EGM to reach, for example, practitioner groups. In the current study, we have drawn on the support of a group of working environment experts within The Danish Working Environment Authority, as they are uniquely positioned to judge what the most pertinent topics are when it comes to enforcing occupational safety and health regulations. In line with Keown (2008), it has been our aim to engage our stakeholders in all parts of the EGM process, starting by consulting with them in the initial process of setting the scope of the EGM and defining relevant outcomes and interventions. This process was initiated in the Fall of 2019 when an open meeting was held between the research team from VIVE and working environment experts from The Danish Ministry of Employment and The Danish Working Environment Authority who expressed their needs for a better overview of existing occupational health and safety research. Having guided us in determining the scope of the EGM, the stakeholders further provided feedback on the title registration and subsequent protocol drafts. This cooperative process has been instrumental in increasing the relevance and precision of the EGM framework. Going forward with the EGM, we have kept our stakeholders updated on the mapping process and the ensuing results, asking for their assessments of, for example, the clarity of presentation and user‐friendliness of the final map. Following publication of the EGM, we will also enlist the support of our stakeholders in determining how best to disseminate the results of the investigation to key interest groups.

In addition to the VIVE research team, three international researchers in working environment issues have contributed as co‐authors of the EGM:

- Professor Peter Hasle, University of Southern Denmark.
- Senior Scientist Hermann Burr, Federal Institute for Occupational Safety and Health (BAuA), Berlin, Germany.
- Senior Scientist Émile Tompa, Institute for Work & Health, Toronto, Canada.

It is important to state that the involvement of The Danish Ministry of Employment and The Danish Working Environment Authority in this EGM has amounted only to consultative support and has not entailed ownership or financial support. Any research decisions made have been the responsibility of the review authors (i.e., the research team from VIVE and the three international researchers) and the conclusions of this EGM are the sole responsibility of the authors.

### 4.4 Conceptual framework

**Figure 1:**
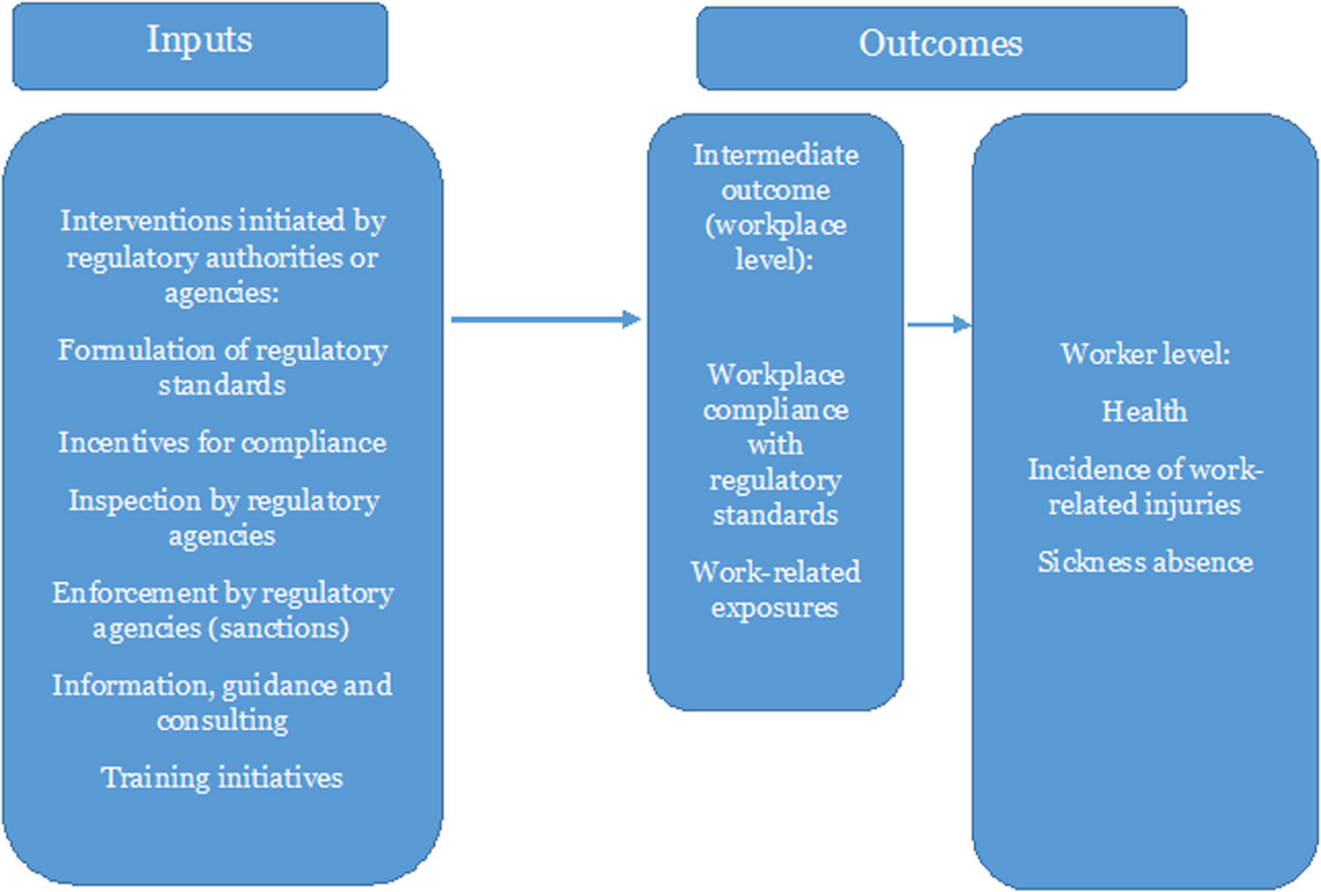
Logic model of interventions and outcomes.

Figure 1 shows the logic model for the interventions and how they link to the outcomes. This does not provide a detailed theory of change of how the interventions may work, but provides a conceptual framework of how we imagine that the inputs in focus may lead to the chosen outcomes. As noted previously, we focus on six types of occupational health and safety regulatory initiatives: (1) formulation of regulatory standards, (2) incentives for compliance, (3) inspection by regulatory agencies, (4) enforcement by regulatory agencies (sanctions), (5) information, guidance, and consulting, and finally (6) training initiatives. These six types of initiatives may have an effect on outcomes through processes of general deterrence, which would be the case if, for example, inspections led to an increased general awareness among businesses of the risk of inspection and potential punishment, resulting in increased compliance with regulatory standards. Interventions may also have specific deterrence effects, as would be the case if actual inspections and consequent punishments of non‐compliant businesses lead to decreases in violations among those punished (Mischke, 2013). As the focus of this EGM is on initiatives by working environment regulatory authorities or regulatory agencies and other organisations assigned as regulators, we have excluded interventions started by individual businesses or employers at their own initiative.

In terms of outcomes, regulatory enforcement is thought to influence health and safety at workplaces both on an organisational (workplace) level and on an individual (worker) level. Workplace compliance with regulatory standards and work‐related exposures are positioned as intermediate outcomes, since the ultimate goal of regulatory enforcement is to reduce negative final outcomes (which are work‐related injuries, health, and sickness absence in this EGM); compliance and reductions in exposure to hazardous materials are essentially means to that end (Tompa, 2016).

### 4.5 Dimensions

As stated previously, this EGM has two primary dimensions: interventions (rows) and outcomes (columns). Additional dimensions in the form of filters are:

1. Study design, including AMSTAR‐rating for systematic reviews.
2. OECD country.
3. Publication status (published or ongoing).

In the following, we specify the criteria that have been used to determine eligibility of references based on study design, interventions, population, and outcomes.

### 4.6 Types of study design

This EGM focuses on the effectiveness of occupational health and safety regulatory interventions, including measures to ensure compliance and/or deter non‐compliance with regulatory standards. To provide an overview of what is known about the effects of such interventions, at the individual and workplace level, we have included all study designs that use a well‐defined control group. The study designs eligible for inclusion in this EGM have therefore been:

1. Randomised controlled trials (RCTs).
2. Non‐randomised studies (NRS), where there is a comparison of two or more groups of participants.
3. Systematic reviews (SR) of effects, provided that they have reported replicable methods to synthesise and summarise the available research evidence and provided that the individual studies fulfil criteria (1) or (2). We have included both systematic reviews in which meta‐analyses were performed and systematic reviews where such analyses were planned, but ended up not being feasible (e.g., due to a low number of included studies). We have excluded systematic reviews in which the authors drew solely on narrative synthesis methods.

We have defined a systematic review and a meta‐analysis in line with the definitions used by PRISMA and The Cochrane Collaboration, in which it is stated that: ‘(…) a systematic review is a review of a clearly formulated question that uses systematic and explicit methods to identify, select, and critically appraise relevant research, and to collect and analyse data from the studies that are included in the review (…) Meta‐analysis refers to the use of statistical techniques in a systematic review to integrate the results of included studies’^1^ (see also Higgins, 2011). We have appraised the quality of systematic reviews using the AMSTAR‐2 tool (Shea, 2017).^2^ The quality of RCTs and NRS has not been appraised, since the purpose of this EGM has been to map the literature, not to make decisions based on individual studies (Welch, 2019).

Studies using single group pre–post comparisons have been excluded. A further requirement posed on all types of studies (randomised as well as non‐randomised) has concerned their ability to identify an intervention effect. Studies where, for example, the treatment was given to one single organisation/company/workplace only and the comparison group was another organisation/company/workplace (or more organisations/companies/workplaces for that matter) would not be able to separate the treatment effect from the organisation/company/workplace effect. Therefore, it has been a requirement that included studies must have at least two organisations/companies/workplaces in both the treatment and comparison group.

Furthermore, NRS using an instrumental variable approach have been excluded—see Supporting Information: Appendix 1 (*Justification of exclusion of studies using an instrumental variable (IV) approach*) for our rationale for excluding studies with these designs.

We have also taken special caution concerning studies using regression discontinuity designs (RDDs) to estimate the treatment effect. In sharp RDDs, a threshold of a (non‐manipulable) forcing/running variable determines which workplaces receive a treatment and which do not, that is, the design is similar to an RCT in the sense that the random sequence determining treatment assignment can be seen as a running variable (Lee, 2008). In contrast, in ‘fuzzy’ RDDs, being on one side of a threshold only makes it more likely that a workplace ends up in the treatment or control group, and the threshold is used as an instrument to estimate local average treatment effects (LATE) (Angrist, 2009; Imbens, 2008). That is, fuzzy RDD is a form of IV analysis, which we have excluded. Sharp RDDs have been included.

Finally, we have not included qualitative research.

Please note that in implementing the above restrictions on types of study designs, we acknowledge that we have limited our focus to a selected field of the occupational health and safety literature on regulatory interventions. We are fully aware of the methodological diversity of this research field and of the value generated by using different types of methodologies to gain insight into the complexities of such interventions. An example of this is the contributions made within the sociology of law, represented by, for example, Keith Hawkins,^3^ which have helped to advance the study of occupational health and safety regulation.

Our approach in this EGM has specifically targeted quantitative effect studies. In conducting the EGM and reporting on its’ results, we wish to make it clear that this study does not aim to give a full and comprehensive overview of the regulatory intervention literature per se; it covers a select subset of studies characterised by the use of distinct, quantitative methods of effect estimation.

### 4.7 Types of intervention/problem

In this EGM, our focus has been exclusively on interventions initiated by working environment regulatory authorities or regulatory agencies and other organisations authorised as regulators, thus excluding interventions started by individual businesses or employers at their own initiative. In Table 1, we present the included intervention categories.

### 4.8 Types of population

The population of relevance to this EGM has been workers above the age of 15 and their workplaces. Conceivably, significant differences exist between the occupational health issues faced by developing nations as opposed to those seen in high‐income countries, which makes it difficult to create a coherent and comparable framework to cover occupational health interventions on a global scale. Therefore, we have chosen to limit our scope to workplaces located in nations within the OECD. Note here that it is the workplaces that had to be located in OECD countries, whereas workers in these workplaces may be citizens of all countries.

### 4.9 Types of outcome measures

Contraventions of occupational health and safety regulatory standards and poor working conditions may result in a number of adverse outcomes of which we have included two intermediate and three final outcomes (see also Figure 1):

- *Workplace compliance with regulatory standards (intermediate outcome)*: Workplace compliance with regional, national, or international working environment regulatory standards concerning, for example, the company's health and safety performance, ergonomics, use of technical machinery, and dangerous chemicals, as monitored by regulatory agencies.
- *Work‐related exposures (intermediate outcome)*: Exposure to substances or hazards that carry the risk of damage to worker health, for example, biological hazards (such as viruses or bacteria), chemical hazards, or ergonomic hazards (due to e.g., manual handling or repetitive, strenuous work positions) (Martinelli, 2019).
- *Health (final outcome)*: Examples of health issues include musculoskeletal disorders, allergies or asthma, hearing loss, and mental ill‐health. Mental ill‐health may include stress symptoms, depressive mood, and absence of wellbeing.
- *Incidence of work‐related injuries (final outcome)*: Covers all injuries, non‐fatal or fatal, resulting from incidents arising out of or in the course of work. This includes traffic accidents happening during working hours.
- *Sickness absence (final outcome)*: Covers short‐ and long‐term periods of absence from work, for example, measured in terms of work days lost.

### 4.10 Other eligibility criteria

As previously noted, we have included only workplaces located in OECD nations.

### 4.11 Search methods and sources

Relevant studies were identified through searches in electronic databases, hand search in journals, grey literature searches, searches in repositories and web resources, citation tracking, and contact to international experts, with the aim of retrieving both published, on‐going, and unpublished studies.

#### 4.11.1 Electronic databases

The following electronic databases were searched in January 2022:

- Academic Search (EBSCO)
- EconLit (EBSCO)
- PsycINFO (EBSCO)
- SocINDEX (EBSCO)
- CINAHL (EBSCO)
- International Bibliography of the Social Sciences (ProQuest)
- Sociological Abstracts (ProQuest)
- Science Citation Index Expanded (Web Of Science)
- Social Sciences Citation Index (Web Of Science)
- MEDLINE (PubMed)
- ERIC (EBSCO)

In determining what databases to search, we consulted the list of databases comprised in the article by Kugley (2017).

#### 4.11.2 Description of search string

The search string was based on the PICO(s)‐model and contained three concepts, of which we developed three corresponding search facets: population, intervention, and study type/methodology. The search string included searches in title, abstract and subject terms for each facet. To increase the sensitivity of the search, we also searched in full text for the intervention terms. The subject terms in the facets were selected according to the thesaurus of each database.

#### 4.11.3 Example of a search string

The search string below was used to search SocINDEX through the EBSCO search interface and exemplifies the search facets:

**Table:** 

#	Query
S15	S4 AND S10 AND S14
S14	S11 OR S12 OR S13
S13	DE (‘effect size’ OR ‘Control Groups’ OR ‘Experimental Groups’ OR ‘Experiments’ OR ‘Matched Groups’ OR ‘Quasiexperimental Design’ OR ‘Randomized Controlled Trials’ OR ‘Comparative Testing’)
S12	AB (((control* OR difference* OR matched* OR random* OR reference* OR compare* OR longitudinal OR cohort*) N3 (group* OR trial* OR test* OR study OR studies OR analy*))) OR AB ((intervent* OR experiment* OR impact* OR ‘systematic review’ OR ‘meta analy*’ OR metaanaly* OR meta‐analy* OR ‘gap map’ OR ‘follow‐up stud*’ OR ‘follow up stud*’ OR ‘followup stud*’))
S11	TI (control* OR difference* OR matched* OR random* OR reference* OR compare* OR group* OR trial* OR test* OR intervent* OR experiment* OR impact* OR ‘systematic review’ OR ‘meta analy*’ OR metaanaly* OR meta‐analy* OR ‘gap map’ OR study OR studies OR analy* OR longitudinal OR ‘follow‐up stud*’ OR ‘follow up stud*’ OR ‘followup stud*’ OR cohort*)
S10	S5 OR S6 OR S7 OR S8 OR S9
S9	SU ((occupational N3 (health OR safety))) OR SU ((work* N3 (health OR safety)))
S8	TI ((occupation* N3 (health OR safety))) OR TI ((work* N3 (health OR safety)))
S7	DE ‘INDUSTRIAL hygiene’
S6	AB ((incentive* OR fund* OR subsid* OR recogni* OR award* OR inspect* OR audit* OR consult* OR sanction* OR penalt* OR fine* OR prosecution* OR ‘enforceable undertaking*’ OR ‘order to comply’ OR citation* OR notification* OR violation* OR breach* OR enforce* OR regulation)) OR AB (((information* OR awareness OR training) N3 (campaign* OR initiative* OR program*)))
S5	TI (incentive* OR fund* OR subsid* OR recogni* OR award* OR inspect* OR audit* OR consult* OR sanction* OR penalt* OR fine* OR prosecution* OR citation* OR notification* OR violation* OR breach* OR ‘enforceable undertaking*’ OR ‘order to comply’ OR information* OR awareness OR training OR enforce* OR regulation)
S4	S1 OR S2 OR S3
S3	(((DE ‘WORK environment’) OR (DE ‘WORK environment—Psychological aspects’ OR DE ‘WORK environment—Research’)) OR (DE ‘ERGONOMICS’)) OR (DE ‘WORK environment—Social aspects’)
S2	AB ((work* OR company OR companies* OR firm OR firms OR organization* OR organisation* OR business* OR institut* OR employe* OR worker* OR staff* OR job) N5 (environ* OR occupational))
S1	TI (work* OR company OR companies* OR firm OR firms OR organization* OR organisation* OR business* OR institut* OR employe* OR worker* OR staff* OR job)) AND TI ((environ* OR occupation*))

**Figure 2:**
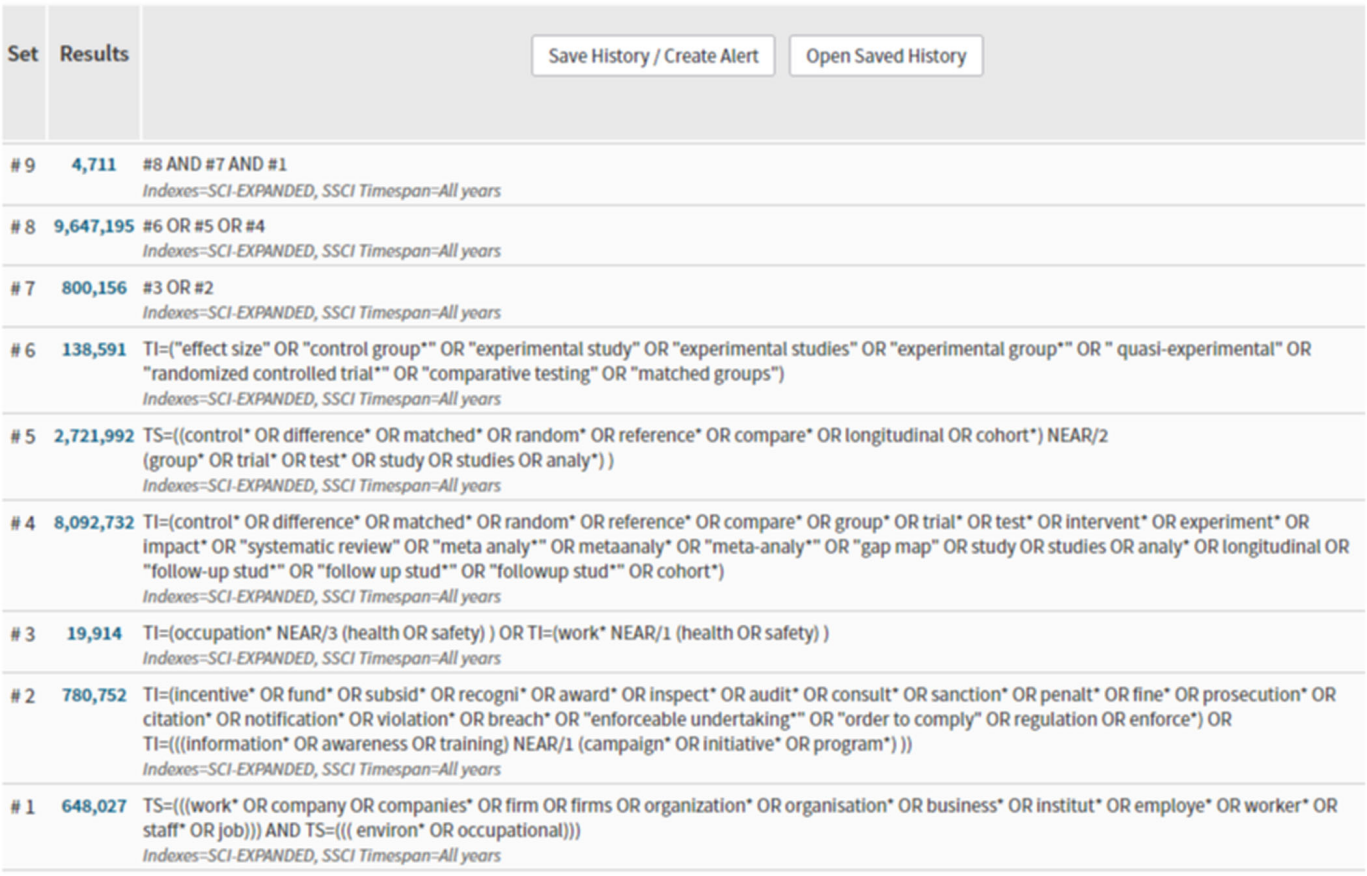
Search in Social Citation Index Expanded (1900–2022) and Social Sciences Citation Index (1956–2022). Search date: 04.01.2022 through the Web of Science platform. Search modes—Boolean/Phrase.

A complete overview of the search strings used and the resulting references found for each database can be found in Supporting Information: Appendix 2 and Figure 2.

#### 4.11.4 Limitations of the search string

There were no language or time limits to our searches (except in the case of hand searches in journals, which were limited to 2015‐present, see next section). In processing the references found, we were however limited by the language proficiencies available on the review team which allowed us to consider studies published in English, German, French, Danish, Norwegian, and Swedish. Potentially relevant studies in other languages were excluded and are listed in Table 2.

**Table 2:** Potentially eligible studies written in language no one in the review team masters.

Study	Notes
Fajkus (1953)	Journal from Slovakia written in Slovenian
Lazzarotto (2017)	Journal from Italy written in Italian
Ruggiero (2018)	Journal from Italy written in Italian
Skowron (2016)	Journal from Poland written in Polish

#### 4.11.5 Hand search in targeted journals

We conducted hand searches of the below mentioned journals, to make sure that all relevant articles were found. The hand search focused on editions published between 01/01/2015 and 31/12/2022 to secure recently published articles which had not yet been indexed in the bibliographic databases.

- *Work & Stress*
- *Policy and Practice in Health and Safety*
- *American Journal of Industrial Medicine*
- *Journal of Labor Economics*
- *Journal of Occupational and Environmental Medicine*
- *International Journal of Environmental Health Research*
- *Scandinavian Journal of Work Environment and Health*
- *Safety Science*
- *Regulation and Governance*
- *Work*
- *Accident Analysis & Prevention*
- *Safety and Health at Work*
- *Journal of Industrial Relations*
- *Law and Policy*
- *Industrial Law Journal*
- *Journal of Safety Research*

Hand searches were performed between September 2022 and January 2023 to ensure that all 2022‐issues were included in the search. An overview of the hand searches and the resulting references can be found in Supporting Information: Appendix 3.

#### 4.11.6 Grey literature searches

We searched for dissertations, working papers, conference proceedings, reports, on‐going studies, and systematic reviews in a comprehensive selection of grey literature venues between September and December 2022. Most of the resources searched included multiple types of references, both published and unpublished. In general, there is a great amount of overlap between the types of references in the chosen resources, but we have listed them according to the category of literature most prevalent in each resource. For the grey literature searches, we used intervention and outcome key words and subject filters where relevant (see Supporting Information: Appendix 4 for full overview of terms and filters used for each resource).

##### Dissertations

We searched the following resources for dissertations:

- EBSCO Open Dissertations (EBSCO‐host)

*Working papers and conference proceedings*

We searched the following resources for working papers/conference proceedings:

- OpenGrey in DANS EASY Archive: https://doi.org/10.17026/dans-xtf-47w5
- Google Scholar: https://scholar.google.com/
- Social Science Research Network: https://www.ssrn.com/index.cfm/en/
- OECD iLibrary: https://www.oecd-ilibrary.org/
- NBER working paper series: http://www.nber.org

In addition, it should be noted that some of the databases included in the database search, for example, Academic Search Premier, CINAHL, PubMed, Science Citation Index Expanded (Web Of Science), and Social Sciences Citation Index (Web Of Science) also contain conference proceedings.

##### Reports

We searched for reports at the websites of governmental institutions and various international organisations as well as in online research repositories and on Google.

- The International Labour Organisation (ILO): https://www.ilo.org/global/publications/lang--en/index.htm
- World Health Organisation—IRIS: https://apps.who.int/iris/
- The European Agency for Safety and Health at Work (EU‐OSHA): https://osha.europa.eu/en
- European Union Senior Labour Inspectors Committee: https://ec.europa.eu/social/main.jsp?catId=148&intPageId=685
- European Foundation for the Improvement of Living and Working Conditions (Eurofound): https://www.eurofound.europa.eu/
- United States Department of Labor ‐ Occupational Safety and Health Administration: https://www.osha.gov/
- Canadian Centre for Occupational Health and Safety: https://www.ccohs.ca/
- The Health and Safety Executive (UK): www.hse.gov.uk
- Safe Work Australia: https://www.safeworkaustralia.gov.au/
- WorkSafe (New Zealand): https://www.worksafe.govt.nz/
- The Danish Working Environment Authority (Arbejdstilsynet): https://at.dk
- The Swedish Work Environment Authority (Arbetsmiljöverket): https://www.av.se/
- The Norwegian Labour Inspection Authority (Arbeidstilsynet): https://www.arbeidstilsynet.no/en/
- CORE—research outputs from international repositories: https://core.ac.uk/
- Google searches: https://www.google.com/
- Best Evidence Encyclopedia: http://www.bestevidence.org/
- The National Research Centre for the Working Environment (NFA): https://nfa.dk/
- The National Institute of Occupational Health in Norway (STAMI): https://stami.no/
- Finnish Institute of Occupational Health (FIOH): https://www.ttl.fi/en/
- ANU—The National Research Centre for OHS Regulation (NRCOHSR): http://regnet.anu.edu.au/research/centres/national-research-centre-ohs-regulation-nrcohsr
- Institute for Work & Health: https://iwh.on.ca
- National Institute for Occupational Safety and Health (NIOSH): https://www.cdc.gov/niosh/
- Federal Institute for Occupational Safety and Health (BAuA): https://www.baua.de/EN/Home/Home_node.html

##### EGMs and systematic reviews

To locate EGMs and systematic reviews, we searched the following resources:

- 3ie Systematic Review Database: https://www.3ieimpact.org/evidence-hub/publications/systematic-reviews
- 3ie Evidence and Gap Map Repository: https://www.3ieimpact.org/evidence-hub/evidence-gap-maps
- Evidence‐Based Synthesis Program (Department of Veteran Affairs): https://www.hsrd.research.va.gov/publications/esp/
- Evidence based policing matrix: https://cebcp.org/evidence-based-policing/the-matrix/
- Campbell Systematic Reviews Journal: https://onlinelibrary.wiley.com/journal/18911803?af=R
- Cochrane Library: https://www.cochranelibrary.com/
- EPPI‐Centre publications: https://eppi.ioe.ac.uk/cms/Default.aspx?tabid=116
- PROSPERO: https://www.crd.york.ac.uk/prospero/
- Open Science Framework: https://osf.io/
- Epistemonikos: https://www.epistemonikos.org/

##### Trial registries

We searched the following trial registries with special attention to relevant on‐going studies:

- AEA Social Science RCT Registry: https://www.socialscienceregistry.org/
- ClinicalTrials.gov: https://clinicaltrials.gov/
- WHO International Clinical Trials Registry Platform: https://www.who.int/clinical-trials-registry-platform
- CENTRAL Trials Register within the Cochrane Library: https://www.cochranelibrary.com/central

When selecting grey literature resources, we consulted the list of websites comprised in the article by Kugley (2017).

A complete overview of search terms and hits for the grey literature resources can be found in Supporting Information: Appendix 4.

##### Citation‐tracking and snowballing methods

We performed citation‐tracking on all included studies (systematic reviews, primary effectiveness studies, and ongoing studies). For the included systematic reviews, we also conducted forwards citation‐tracking through Google Scholar and Web of Science to identify potentially relevant references that had cited these reviews.

In addition to citation‐tracking the included references, we tracked the reference lists of a number of other reviews, research overviews, and primary studies identified during the search process that we judged to potentially contain relevant references.

A table documenting the citation‐tracking process of included and additional studies is provided in Supporting Information: Appendix 5, with number of references screened for each study.

*Contact to experts* We drew on the content knowledge expertise of our stakeholders at The Danish Working Environment Authority and our three international co‐authors (Hermann Burr, Peter Hasle and Émile Tompa) in identifying additional relevant references that were not located in our primary searches. This was done by forwarding a reference list of the included studies found through our database searches and asking the experts to identify any references they knew of that were not already on the list.

### 4.12 Analysis and presentation

#### 4.12.1 Report structure

In the following, we will present the results of our mapping and discuss the implications of our results in terms of identifying areas covered by sufficient evidence and areas characterised by evidence gaps. It should be noted that several of the interventions in this map are multi‐faceted, meaning that they contain elements from more than one of the intervention categories included in our conceptual framework. Our approach has been to list studies under each relevant intervention category since the primary purpose of the map is to show the existence of evidence for each particular combination of intervention and outcome, and some interventions (and studies) may provide evidence relevant to more than one such combination.

In reporting our results, we will provide the following tables and figures:

- PRISMA flowchart.
- Number of studies by intervention and outcome category.
- Number of studies by study design.
- AMSTAR‐rating of systematic reviews.
- Number of studies by country.

#### 4.12.2 Filters for presentation

Interventions and outcomes make up the primary dimensions of our map, with coding of the following filters:

1. Study design, including AMSTAR‐rating of systematic reviews.
2. OECD country.
3. Publication status (published or ongoing).

### 4.13 Dependency

In cases where we have located multiple references reporting the same study results, we use the latest or most complete version in the map. That is, we include the version that reports most fully on the outcomes, and in case of no differences between reports, the latest edition. The following included studies: Kemmlert (1996), Rautiainen (2008), van der Molen (2018), Johnson (2019), and McLeod (2019) have one or more secondary references which are listed under their primary references in the list of included studies (see Included studies).

Furthermore, the systematic reviews included in the map contain numerous of the primary studies shown on the map. Our approach has been to include all relevant primary studies whether they have been included in a systematic review or not, and we have performed ‘un‐zipping’ of the systematic reviews, meaning that we have ensured that all primary studies included in the systematic reviews are also included individually on the map.

### 4.14 Data collection and analysis

#### 4.14.1 Screening and study selection

Screening on both title/abstract and full text was performed in duplicate by the review authors and a team of research assistants. Two persons first independently screened titles and abstracts to exclude clearly irrelevant studies. Studies considered eligible by at least one person or studies where there was insufficient information in the title and abstract to judge eligibility were retrieved in full text. The full texts were then screened independently by two persons, with any doubts or disagreements of eligibility resolved by the review authors. See Supporting Information: Appendix 6 for the screening guide used. None of the review authors were blind to the authors, institutions, or journals responsible for the publication of articles.

Exclusion of studies that otherwise might be expected to be eligible is documented and presented in Tables 3 and 4.

**Table 3:** Studies excluded with reasons.

Study	Reason
Arocena (2009)	Before‐after study.
Baggs (2001)	Outcome is compensable claims rates, which is not an eligible outcome in this EGM.
Baggs (2003)	Outcome is compensable claims rates, which is not an eligible outcome in this EGM.
Burstyn (2010)	Outcomes not relevant to this EGM.
Carpenter (2009)	Can only identify the location of the respondent's residence, not the location of work (which is the unit of analysis). In general only three‐quarters of workers aged 16 and older in the United States live and work in the same county.
Cooke (1981)	States that: ‘To assess OSHA's effectiveness, we regress the number of citations OSHA inspectors rendered per plant through June, 1976 against the change in injury rates from 1970 to 1976 (ceteris paribus)’. This means that in theory, the decrease could have occured before inspection or they could have experienced an increase in 1975–1976, had an inspection in 1976, and then be counted in the treated group.
Farina (2016)	Compares companies with either a planned investigation (PI) or an accident investigation (AI) and perform survival analysis with first accident after inspection as event.
Gray (1991)	Employs fixed effect (BA) although treatment dummy is in levels and equal to 1 if treated at any time during the last x (varies) years (i.e., no well defined control group and both groups are a mix of treated and control at some point) and Tobit models.
Gray (1991a)	Fixed effects version of the Poisson and negative binomial count models, outcomes are citations and overexposure.
Gray (1993)	Fixed effect (BA). This study controlled for firm‐level fixed effects, which means they examined within‐firm changes over time.
Gray (2005)	Employs fixed effect (BA) although treatment dummy is in levels and equal to 1 if treated at any time during the last x (varies) years (i.e., no well defined control group and both groups are a mix of treated and control at some point) and Tobit models.
Haviland (2010)	Uses the method applied in the Gray articles although only 2 years of inspection but still firms can be in treated group in some years and in control in others.
Haviland (2012)	Uses the method applied in the Gray articles although only 2 years of inspection but still firms can be in treated group in some years and in control in others.
Imamura (2018)	Before‐after study.
Ko (2010)	Outcome is citations (proxy for compliance, number of violations cited) at inspection number 1–5. Includes only firms with multiple inspections and outcome is change in number of violations cited.
Lee (2022)	Outcome (injury compensation claims) not relevant to this EGM.
Li (2019)	Uses fuzzy regression discontinuity.
Mendeloff (2005a)	Uses the method applied in the Gray articles meaning firms can be in treated group in some years and in control in others.
Mendeloff (2005b)	Uses the method applied in the Gray articles meaning firms can be in treated group in some years and in control in others.
Parker (2016)	No control group and participation is voluntary.
Peek‐Asa (2004)	The intervention/course is not specifically demanded by an authority.
Scholz (1990)	Estimates the effect of predicted probability of an inspection with penalty (i.e., not just inspection) and effect of amount of penalty, given an inspection with penalty. It is a first difference estimation.
Smitha (2001)	Industry is unit of analysis.

Abbreviation: EGM, evidence and gap map.

**Table 4:** Reviews excluded with reasons.

Review	Reason
Feltner (2016)	Outcomes not relevant and interventions do not seem to be of a regulatory nature.
Johannessen (2017)	No meta‐analysis, vote counting model, includes BA‐studies.
Tompa (2016)	No meta‐analysis, vote counting model, includes BA‐studies.
Tompa (2007)	Narrative review (best evidence synthesis).

### 4.15 Data extraction and management

Data extraction was performed in EPPI‐Reviewer (EPPI‐Reviewer) using a codeset designed to accommodate the creation of the EGM in EPPI‐Mapper (EPPI‐Mapper). The codeset included clickable options with free‐text fields supplied to capture all relevant details surrounding the publication itself, the study design, intervention and outcome domains, and population details, as shown in Supporting Information: Appendix 7. The extraction was performed by a research assistant or review author and independently checked by another member of the review team.

#### 4.15.1 Tools for assessing risk of bias/study quality of included reviews

**Figure 3:**
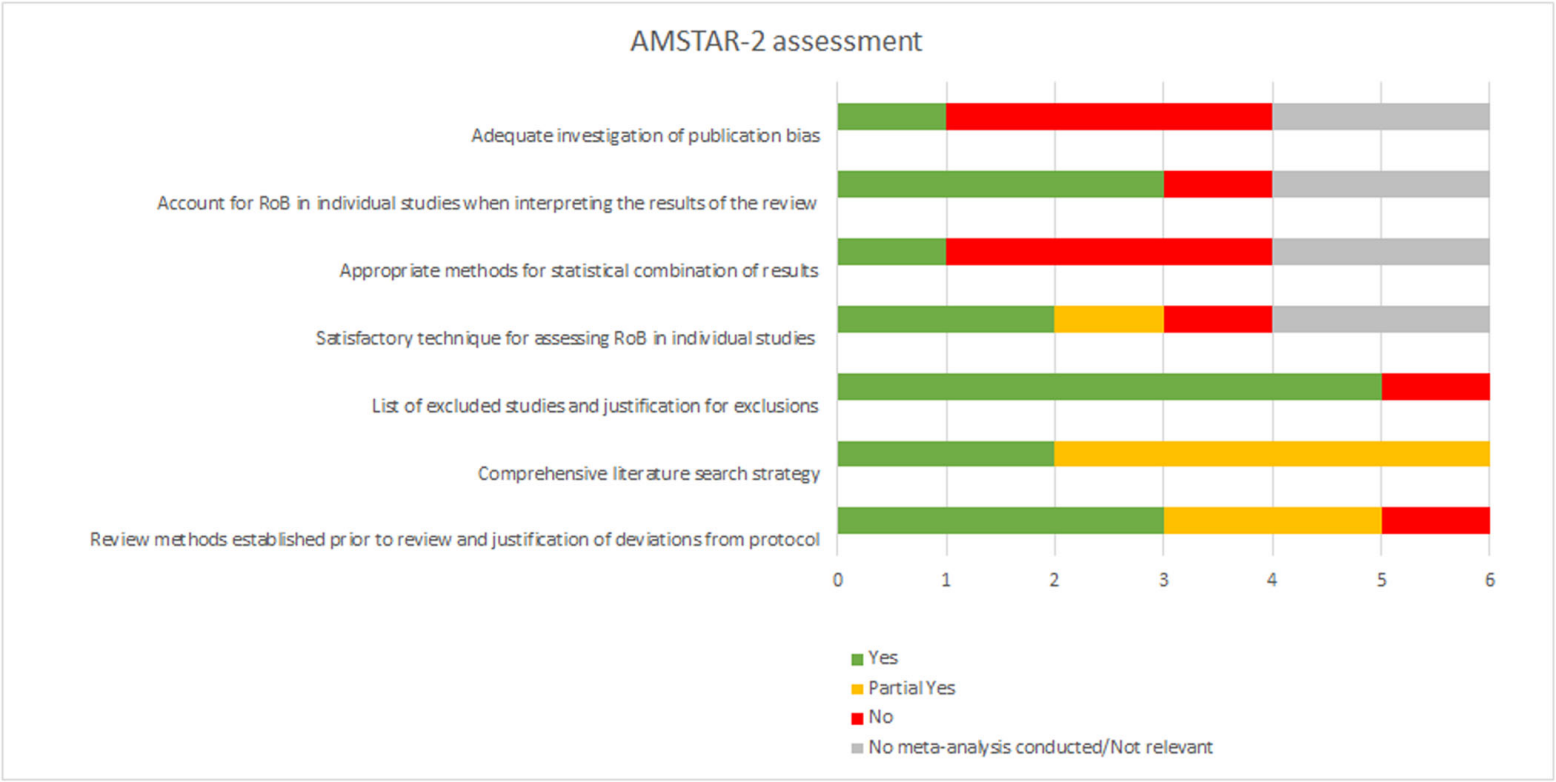
Assessment of critical domains of the AMSTAR‐2 tool.

Since systematic reviews are often used for decision making, we performed critical appraisals of the included systematic reviews using AMSTAR‐2 (Shea, 2017), which covers 16 domains, seven of which are critical (these critical domains are shown in Figure 3). The 16 domains take the assessor through the integral parts of the review process, from protocol to literature search and over to risk of bias assessment, meta‐analytic procedures, and exploration of potential publication biases, ending in an overall rating of high, moderate, low, and critically low confidence. The overall rating is based on the existence of critical or non‐critical weaknesses in the 16 domains. We used the full AMSTAR‐2 tool, but added three additions that we found to be important for the critical appraisal: (1) assessment of whether the authors present a plan for handling dependent effect sizes, (2) assessment of whether risk of bias assessments of included studies are performed in duplicate, that is, performed independently by two reviewers after which a consensus coding is agreed upon, and (3) assessment of the authors’ investigation of causes for heterogeneity under the domain covering statistical combination of results in the meta‐analysis. The overall rating for the systematic reviews is included in the study design filter of the interactive map. It should be noted that we performed quality appraisal of the included systematic reviews in duplicate, that is, two reviewers independently assessed each review and reached a consensus, with the involvement of a third reviewer in cases of uncertainty or disagreement. We did not assess the quality of included primary effect studies, since the purpose of the EGM was to identify existing studies, not to make decisions based on single trials (Welch, 2019).

#### 4.15.2 Methods for mapping

We used EPPI‐Mapper software (EPPI‐Mapper), powered by EPPI‐Reviewer (EPPI‐Reviewer), to generate an online, interactive map, with interventions and outcomes serving as the primary dimensions of the map. In addition to interventions and outcomes, the map contains the following filters:

- Study design, including AMSTAR‐rating of systematic reviews.
- OECD country.
- Publication status (published or ongoing).

When clicking a bubble on the interactive map, a list of studies for that particular intervention and outcome domain is shown, with abstract and main publication details provided for each study. By clicking on the ‘Filters’ tab, clickable boxes will appear which enables the viewer to choose that only studies using one or more particular designs, studies carried out in specific OECD countries, or studies with a certain publication status (e.g., only completed studies) are shown on the map.

## 5 RESULTS

### 5.1 Description of studies

#### 5.1.1 Results of the search

**Figure 4:**
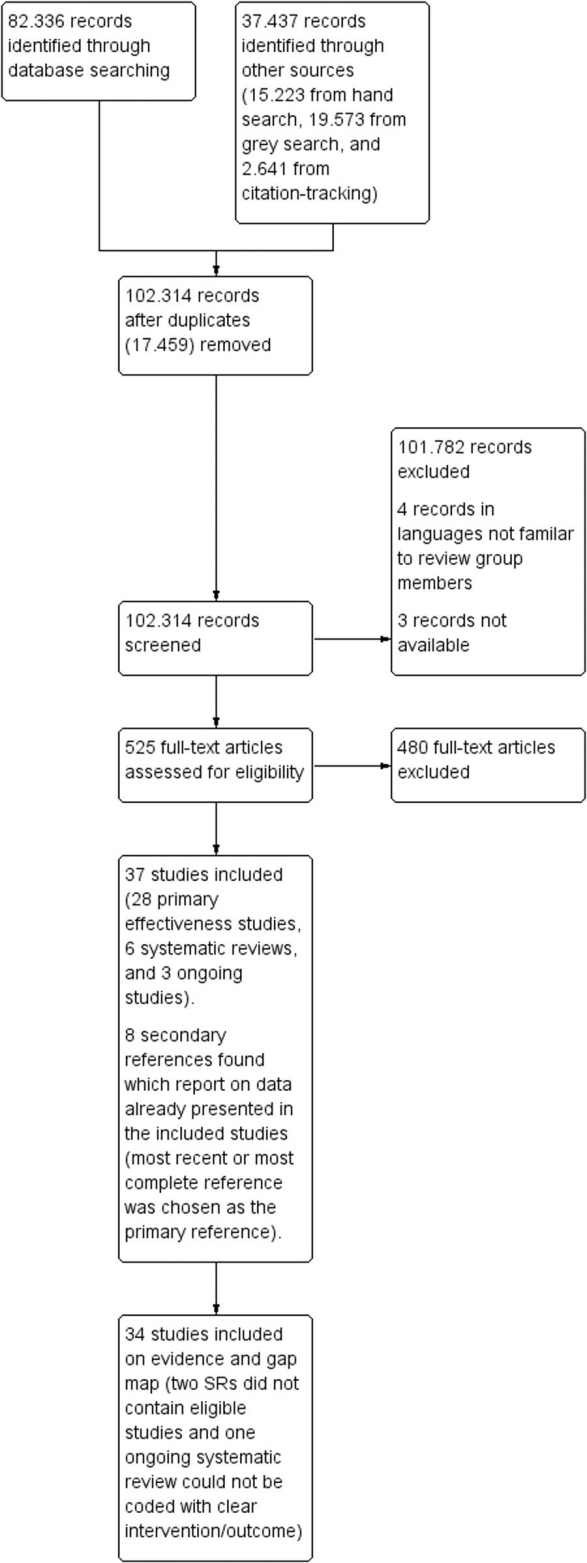
Flowchart.

We summarise the search results in a flow chart in Figure 4. The total number of potential relevant studies was 102.314 after excluding duplicates (electronic databases: 82.336, grey literature: 19.573, hand searches: 15.223, and citation‐tracking: 2.641). We screened all studies based on title and abstract; 101.782 references were excluded for not fulfilling the screening criteria. Four studies were written in a language no one in the review team mastered (they are listed in Table 2). Three studies were unobtainable despite efforts to locate them through libraries and searches on the Internet (these studies are listed in Table 5). Accordingly, 525 studies were ordered, retrieved, and screened in full text. Of these, 480 studies did not fulfil the screening criteria and were excluded. As a result, we located a total of 37 studies, of which six were systematic reviews (and in addition, there were 4 secondary systematic reviews, that is, if a systematic review was updated, we characterised the latest publication as the primary review and the rest as secondary), 28 were primary effect studies (in addition, there were 4 secondary effect studies, that is, if the same intervention using identical data was reported in several papers, we characterised the latest publication as the primary study and the rest as secondary), and three were on‐going studies. References to the 37 included studies (and their secondary references) are listed in Included studies and Ongoing studies.

**Table 5:** Studies not available.

Study	Notes
ERG (2004)	Found on the United States Department of Labor's CLEAR (Clearinghouse for Labor Evaluation and Research) website: https://clear.dol.gov/study/evaluation-osha%E2%80%99s-impact-workplace-injuries-and-illnesses-manufacturing-using-establishment
Mendeloff (1976)	Report prepared for the Office of the Assistant Secretary for Policy, Evaluation and Research (ASPER), U.S. Department of Labor, Washington DC, 1976.
Macpherson (2021)	Final report submitted to the Saskatchewan Workers’ Compensation Board. Partnership for Work, Health and Safety. School of Population and Public Health, University of British Columbia, Vancouver, BC.

The six systematic reviews all had broader inclusion criteria for including studies than what is the case for this EGM; that is, they aimed to locate both studies relevant to this EGM (i.e., studies investigating the effects of regulatory enforcement using designs with a well‐defined control/comparison condition) as well as studies irrelevant to this EGM (i.e., studies not investigating regulatory interventions or not using a design with a control/comparison condition). All six systematic reviews were therefore included in this report based on their inclusion criteria. However, the searches performed in the six systematic reviews did not necessarily result in the location of studies of relevance to this EGM. In some cases, only studies not eligible for this EGM were located (i.e., studies that either did not investigate the effects of regulatory enforcement or did not use an eligible study design). Studies that did not end up including eligible studies are described and listed in this report, but not shown on the interactive map. The following four systematic reviews included studies relevant to this EGM and are accordingly shown on the interactive map: Andersen (2019); Dyreborg (2022); Mischke (2013); van der Molen (2018). The remaining two systematic reviews which were Rautiainen (2008) and Cashman (2009) did not include any studies relevant to this EGM and are therefore not shown on the map.

The primary effect studies predominantly included NRS; 24 of the effect studies were NRS and three were RCTs. The following studies used non‐randomised designs: Agnesi (2016); Alwall (2020); Björkdahl (2008); Chen (2008); Dahl (2013); Dahl (2022); Foley (2012); Johnson (2019); Kim (2021); Lewchuk (1996); López‐Ruiz (2013); Macpherson (2022); Mancini (2005); McCaffrey (1983); McLeod (2018); McLeod (2019); Moran (1985); Nelson (1997); Rubio‐Romero (2015); Ruser (1991); Smith (1979); Stocks (2015); Weil (1996); Wickizer (2004). The following studies used randomised controlled designs: Hogg‐Johnson (2012); Kemmlert (1996); Soriano‐Serrano (2020). Finally, Peto (2016) was both an RCT and a NRS‐study.

The three on‐going studies included an RCT (Abildgaard, 2021), a CRCT (Indregard, 2019), and a systematic review (Sundstrup, 2022). The ongoing systematic review is not shown on the interactive map, since no clear intervention/outcome categories could be coded based on the trial information available at the time of writing this report.

**Figure 5:**
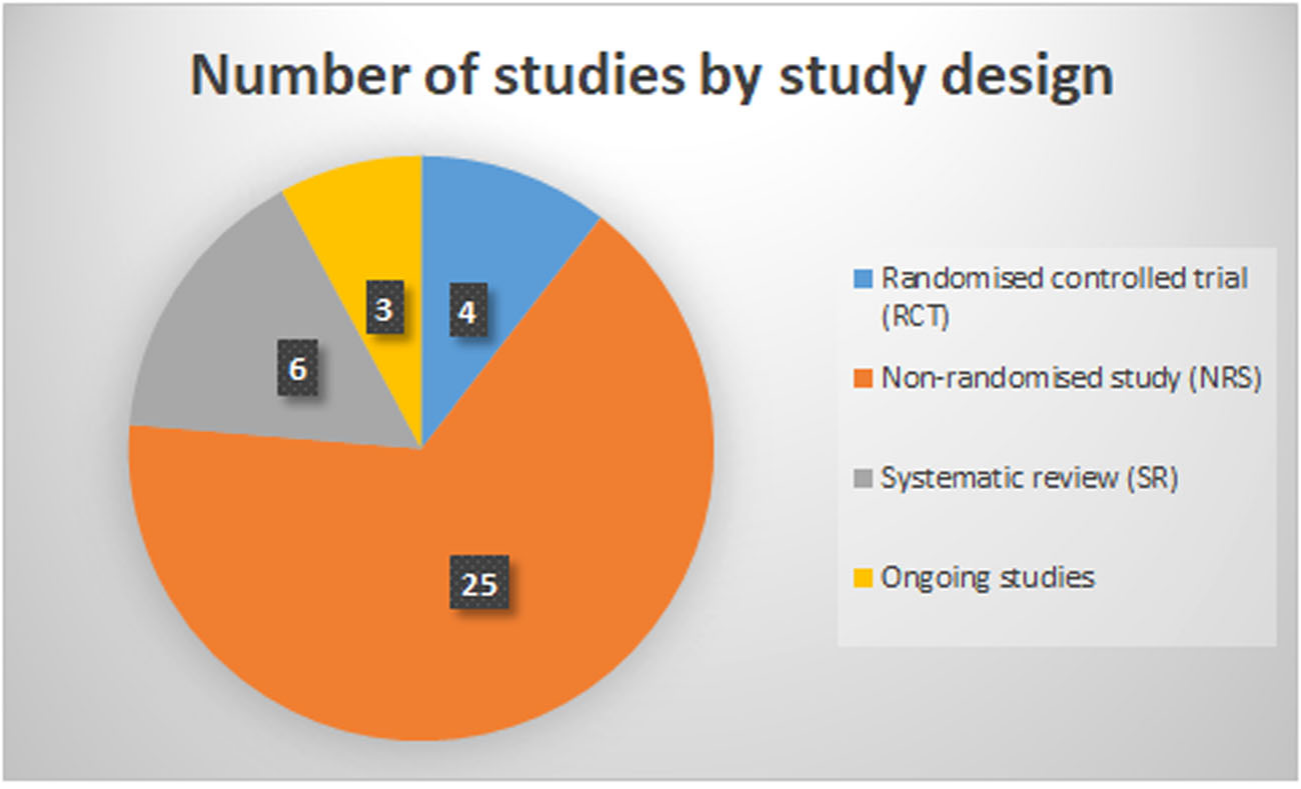
Number of studies by study design. Please note that one study used both an randomised controlled trial and a non‐randomised studies design and is therefore counted in both categories.

For a visualisation of the division of included studies by study design, see Figure 5.

#### 5.1.2 Excluded studies

In addition to the 37 studies that met the inclusion criteria for this map, 27 studies (excluding 5 secondary studies), of which four were systematic reviews, at first sight appeared relevant but did not meet our criteria for inclusion. These studies and reviews are listed in Excluded studies and the reasons for exclusion are given in Tables 3 and 4.

### 5.2 Synthesis of included studies

The interactive EGM contains 34 studies (since two systematic reviews did not include studies relevant to this map, and one ongoing systematic review did not contain sufficient information to allow a clear intervention/outcome coding, as described in ‘Results of the search’). A link for the interactive map is provided in Supporting Information: Supporting Information: Appendix 8 and additionally, Table 6 shows the number of primary studies, systematic reviews, and ongoing studies for each combination of intervention and outcome (with systematic reviews and ongoing studies reported in brackets). When looking at the map, it is immediately clear that the intervention category covering inspection activity is the most densely populated, with a total of 21 studies, five of which are RCTs (one is an ongoing study), 14 are NRSs, and three are systematic reviews (note that one study is both an RCT and a NRS and therefore counted in both categories). The studies exploring the effects of inspections cover all outcome categories, but with most studies located in the injuries domain (15 studies, where one study is counted as both an NRS and an RCT), followed by compliance (11 studies, where one study is counted as both an NRS and an RCT), health (three studies, where one study is counted as both an NRS and an RCT), work‐related exposures (three studies), and finally sickness absence (which is covered by only one ongoing study).

**Table 6:** Number of primary studies by intervention and outcome category (with number of systematic reviews [SR] and ongoing studies in brackets).

	Compliance with regulation or regulatory action	Work‐related exposures	Incidence of work‐related injuries	Health	Sickness absence
Formulation of regulatory standards	0	0	1	0	0
Incentives for compliance	1 (both NRS and RCT)	0	6 (one both RCT and NRS), (1 SR)	1 (both NRS and RCT)	0
Inspection by regulatory agency	7 (one both RCT and NRS), (3 SR, 1 ongoing)	1 (2 SR)	11 (one both RCT and NRS), (3 SR, 1 ongoing)	1 (both RCT and NRS), (1 SR, 1 ongoing)	(1 ongoing)
Enforcement by regulatory agency (sanctions)	1 (3 SR)	(2 SR)	1 (3 SR)	(1 SR)	0
Information, guidance and consulting activity	1 (2 SR, 2 ongoing)	1 (1 SR)	4 (3 SR, 1 ongoing)	1 (1 SR, 1 ongoing)	(1 ongoing)
Training initiatives	1	1	0	0	0

Abbreviations: NRS, non‐randomised studies; RCT, randomised controlled trial.

The intervention category covered by the second most studies is that of ‘Information, guidance, and consulting activity’, where 12 studies are located, three of which are RCTs (two are ongoing studies), six are NRSs, and three are systematic reviews. In terms of outcomes, the injuries domain is again the most densely populated with eight studies (one of which is ongoing), followed by compliance (five studies, two of which are ongoing), health (three studies, one of which is ongoing), work‐related exposures (two studies), and sickness absence (only one ongoing study).

There are seven studies investigating the effects of incentives for compliance, including one RCT, six NRSs, and one systematic review (note that one study is both an RCT and a NRS and therefore counted in both categories). All seven studies in this intervention category include injury incidence as an outcome, with one study additionally looking at health outcomes and compliance (counted as both and RCT and a NRS and therefore illustrated with two bubbles). There are no studies investigating the effects of incentives for compliance on work‐related exposures or sickness absence.

Five studies are focused on the effects of enforcement activities: one RCT, one NRS, and three systematic reviews. Four of these studies include injury incidence as an outcome, four studies include a compliance outcome, two studies include work‐related exposures, and one study includes health outcomes. As was the case for the incentives category, there are no studies investigating the effects of enforcement activity on sickness absence.

The final two intervention categories, training initiatives and formulation of regulatory standards, are only scarcely populated, with two NRS studies for the training domain and one NRS study for the formulation of regulatory standards domain. The NRS study investigating the effects of setting regulatory standards includes injury incidence as an outcome, and as for the two studies exploring training initiatives, one study includes work‐related exposures and one includes compliance as an outcome.

Reflected in the above summation of the map is the fact that we find both relatively well‐populated intervention and outcome domains and domains which are empty or only very scarcely covered, the latter of which points to the existence of gaps in the evidence concerning the effects of occupational health and safety regulatory interventions. As for the well‐populated domains, there is an evidence base available to those seeking information about the effects of inspection activity, especially related to injury incidence and compliance outcomes. The same is the case for the effects of information, guidance, and consulting activities and incentives for compliance, again centred on incidence of work‐related injuries. There is furthermore a smaller evidence base covering the effects of regulatory enforcement in the form of sanctions, scattered with relatively few studies for each outcome domain (excluding sickness absence which has no studies in this intervention domain). When it comes to the evidence gaps identified by this map, two main areas stand out. Firstly, the sickness absence outcome domain is almost empty (populated by only one ongoing study located under two intervention domains). While it is possible that sickness absence is in some cases integrated into or covered by injury incidence or health outcomes, the lack of studies for this domain is still conspicuous and points to a relevant venue for new research. Similarly, two intervention categories, training initiatives and formulation of regulatory standards, are very scarcely populated, meaning that at present point, there is little guidance in the literature as to how regulatory agencies can most effectfully enact new regulatory standards or implement training initiatives.

**Figure 6:**
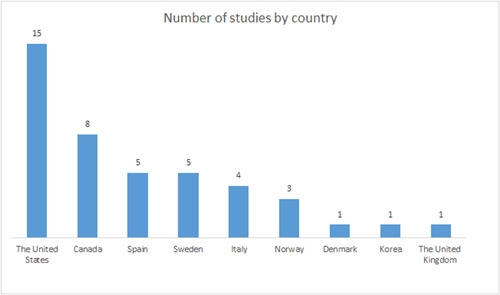
Number of studies by country. Please note that included systematic reviews may include studies from several countries.

In terms of the geographical spread of studies across the OECD, the country filter of the interactive map and Figure 6 illustrates that most studies were conducted in The United States (15 studies), followed by Canada (eight studies), Spain and Sweden (five studies each), Italy (four studies), and Norway (three studies). Finally, South Korea, The United Kingdom, and Denmark are represented by one study each. It should be noted that the systematic reviews were coded according to which countries were represented in their included studies, meaning that a systematic review could have more than one country code. As is evident from Figure 6, North America (USA and Canada) is the most densely populated area in terms of number of studies, followed by Europe (represented by countries located in both the Northern and Southern parts of the continent). Finally, Eastern Asia is represented by one study from South Korea.

We had originally considered implementing a filter on the map for work sectors/industrial groupings, but refrained from doing so since detailed information about work sectors was not available or unclear in a number of studies, and there was no consistent sector division or typology common to the included studies. However, it was evident that the manufacturing and construction sectors were well represented in the included studies.

### 5.3 Risk of bias in included reviews

We located six systematic reviews in our searches and critically appraised their quality using AMSTAR‐2 (with three additions, see ‘Tools for assessing risk of bias/study quality of included reviews’). The full rating with justification for each domain is documented in Supporting Information: Appendix 9 and Figure 3 gives a visual illustration of our assessment of critical domains of the AMSTAR‐2 tool for the six included systematic reviews. As a result of the appraisal, three systematic reviews received a ‘critically low’ rating, one received a ‘low’ rating, and two did not receive a full rating due to them not containing studies eligible for this EGM.

The four systematic reviews that received a full AMSTAR‐rating all included both studies which were eligible for this EGM (relevant intervention, outcome and study design) and studies which were ineligible (e.g., interrupted time series designs without a control group). When performing the AMSTAR‐assessments, we focused only on the authors’ treatment of studies eligible for this EGM. As for the three systematic reviews with ‘critically low’ confidence, main limitations included the absence of appropriate methods for statistical combination of results in the meta‐analysis and inadequate investigation of the potential impact of publication bias. Furthermore, heterogeneity was not sufficiently assessed and discussed. According to the AMSTAR‐guideline, ‘critically low’ confidence entails that the review has more than one critical flaw (with or without non‐critical weaknesses) and should not be relied on to provide an accurate and comprehensive summary of the included studies. As for the review with low confidence, the only flaw was a failure to adequately investigate publication bias; otherwise, this review was well‐performed and followed standard systematic review procedures.

The two systematic reviews without eligible studies for this EGM were those of Rautiainen (2008) and Cashman (2009). Rautiainen (2008) aimed to include randomised controlled trials, cluster‐randomised controlled trials, prospective cohort studies with a concurrent control group, and interrupted time series studies. However, the literature search only located studies irrelevant to this EGM (five RCTs/CRCTs that did not investigate regulatory enforcement and three ITS studies with no control group). Therefore, the AMSTAR‐assessment of this review was only performed in relevant domains (e.g., risk of bias and meta‐analysis domains were not relevant when no eligible studies were involved) and no overall AMSTAR‐rating was given. Cashman (2009) aimed to include RCTs, cluster‐randomised trials, controlled clinical trials, controlled before‐and‐after studies (more than three time points to be measured before and after the study), and interrupted time‐series (ITS) studies. However, as a result of the searches, authors located just two interrupted time‐series studies, none of which were relevant to this EGM, and the AMSTAR‐assessment was performed accordingly, with no overall rating given.

Overall, our assessment of the included reviews suggests that there is a lack of high quality systematic reviews and meta‐analyses exploring the effects of occupational health and safety regulatory interventions. This points to the relevance of commissioning and performing more rigorous systematic reviews in areas sufficiently covered by primary effectiveness studies. One such area which stands out in the current EGM is that of inspection activity which is populated by 17 completed primary studies and one ongoing cluster‐randomised controlled trial.

## 6 DISCUSSION

### 6.1 Summary of main results

After performing comprehensive searches for both published and unpublished literature, we ended up with a total of 37 studies, six of which were systematic reviews (with four related secondary systematic reviews), 28 of which were primary effect studies (with an additional four related secondary effect studies), and three of which were on‐going studies. The 28 primary effect studies predominantly used non‐randomised designs. It should be noted that since two of the systematic reviews did not contain intervention and outcome domains and/or study designs eligible for this EGM and one ongoing systematic review did not contain sufficient information to allow a clear intervention and outcome coding, the final map includes 34 studies. Furthermore, as a result of the AMSTAR‐rating, three systematic reviews received a ‘critically low’ rating, one received a ‘low’ rating, and two did not receive a full AMSTAR‐rating due to them not containing studies eligible for this map.

As noted in Results, this EGM uncovers both relatively well‐populated and empty or nearly empty intervention and outcome domains. To sum up, there is an evidence base available concerning the effects of inspection activity on injury incidence and compliance outcomes as well as for the effects of information, guidance, and consulting activities and incentives for compliance on incidence of work‐related injuries. Additionally, there is some evidence covering the effects of regulatory enforcement in the form of sanctions.

### 6.2 Areas of major gaps in the evidence

The map identifies two main evidence gaps: (1) there is a lack of evidence for the sickness absence outcome domain, which is populated by only one ongoing study, and (2) there is very limited evidence available concerning the effects of regulatory agencies formulating regulatory standards and carrying out training initiatives. Furthermore, our assessment of the included reviews points to a lack of rigorously performed systematic reviews and meta‐analyses investigating the effects of occupational health and safety regulatory interventions. It is therefore of relevance to researchers and research commissioners to consider initiating more systematic reviews in areas sufficiently covered by primary effectiveness studies, such as that of inspection activity which is well populated on this EGM.

### 6.3 Potential biases in the mapping process

For this EGM, we performed extensive searches for both published and unpublished literature and screened the records found in duplicate. Furthermore, coding of included studies and assessment of systematic reviews with the AMSTAR‐tool was also performed in duplicate and thoroughly checked. There was a small number of potentially relevant studies which were excluded due to inavailability or language proficiency restrictions within the review group. Otherwise, we believe that we have taken all possible precautions to eliminate or diminish the potential for biases in the mapping process.

### 6.4 Limitations of the EGM

As is the case for all types of evidence maps or syntheses, it has been necessary to implement restrictions on types of interventions and outcomes, types of population (only OECD countries), and types of study designs to create a coherent map and secure a manageable mapping process in terms of number of references (note that we have processed more than 100.000 references for this map). This means that, for example, other types of work environment interventions initiated by workplaces or other non‐regulatory institutions are not covered by the current EGM, just as research drawing on other methods, be it quantitative studies without control/comparison conditions, qualitative studies, or narrative reviews, is not included. Also, this EGM only covers the OECD and not, for example, developing nations. It is important for us to stress that the above restrictions are implemented from a wish to ensure coherence and adequate detail in the final map and to create a manageable workload, not from a perspective of, for example, judging some methods or study designs as better than others or to suggest that work conditions outside the OECD are of less relevance. We acknowledge that we have limited our focus to a select field of effectiveness research within a limited set of nations and are fully aware of the diversity and value generated by using different types of methodologies and exploring work conditions across a wide array of geographic locations, just as we are aware of the inherent complexity of regulatory processes and the challenges in performing controlled trials in this field. Since the effects of regulatory measures are complex and related to a number of mechanisms and processes in the overall regulatory system, there is a need for more research drawing not only on quantitative effect measures, but also on qualitative and mixed methods approaches exploring the depths and complexities of occupational safety and health regulatory enforcement in different institutional structures.

### 6.5 Stakeholder engagement throughout the EGM process

We are very grateful for the engagement of our stakeholders within The Danish Working Environment Authority throughout the process of planning, designing, and completing this map. Their engagement has played a central role in defining the scope of the map and developing a coherent framework of interventions and outcomes. Furthermore, we have received constructive feedback on both the title registration, protocol, and the final report. Following publication of the EGM, we will cooperate with our stakeholders disseminating our findings to key interest groups.

## 7 AUTHORS’ CONCLUSIONS

### 7.1 Implications for research, practice and/or policy

This EGM of systematic reviews and primary effect studies uncovers both well‐populated and empty or nearly empty intervention and outcome domains in the area of occupational health and safety regulatory intervention research. Most evidence is available concerning the effects of inspection activity, but we have also located a number of studies investigating information, guidance, and consulting activities, incentives for compliance, and, to a lesser extent, regulatory enforcement in the form of sanctions. On the other hand, the map points to a lack of studies including sickness absence as an outcome and a very limited evidence base concerning the effects of formulating regulatory standards and carrying out training activities (initiated by regulatory agencies). More research using rigorous effectiveness designs is therefore needed to better cover these intervention and outcome domains such that work environment authorities, lawmakers, administrators, practitioners, and other key stakeholders may find guidance in their endeavours to improve work conditions and prevent adverse outcomes for workers. This call for more research is however not limited to quantitative effectiveness research, but extends to other types of research as well, including mixed methods designs and in‐depth qualitative inquiries which are needed to shed light on the complex mechanisms behind how different types of occupational health and safety regulatory enforcement function and interact with other processes taking place within a given institutional system.

Furthermore, based on our assessment of the included systematic reviews, we find that there is a need for more high quality reviews investigating the effects of all types of occupational health and safety regulatory interventions, as well as for rigorous systematic reviews of other types of research literature exploring central processual or institutional structures within different regulatory systems. One suggestion based on the evidence located with this map would be to carry out a focused systematic review and meta‐analysis of the effects of inspection activity since this domain is relatively well‐populated by primary effect studies. It should be noted that since we did not perform risk of bias assessments of the included primary studies, no judgments on the quality of primary effectiveness studies within this research field can be made on the basis of this EGM.

Finally, this EGM only includes effect studies investigating workplaces located in OECD member countries. It is of utmost relevance to perform similar mapping procedures for studies performed, for example, in developing nations and more generally, to explore the particular conditions surrounding work environment regulations in different regions of the world with an array of different research methods.

## CONTRIBUTIONS OF AUTHORS

**Content:**

*Jan Hyld Pejtersen, Hermann Burr, Peter Hasle, and Émile Tompa*

*Jan Hyld Pejtersen* is a senior researcher, PhD, and has worked with occupational health and safety for more than 25 years. Jan has studied the association between psychosocial work environment and health both in population studies and in studies at the workplace level. Jan is the author and co‐author of several peer‐reviewed articles in the field of occupational health and safety.

*Hermann Burr* is a senior researcher, PhD, at Bundesanstalt für Arbeitsschutz und Arbeitsmedizin, Berlin. Hermann is a specialist in social epidemiology and his research deals with health and labour market effects of working conditions among employees. He is also active in the international COPSOQ network and in the Work IPD consortium and has authored numerous peer‐reviewed articles in the field of occupational health.

*Peter Hasle* is a Professor in Sustainable Production at The Department of Technology and Innovation, University of Southern Denmark. His research interests include sustainable work‐ and production processes. Peter has worked with occupational health and safety research for more than 40 years and is the author and co‐author of numerous peer‐reviewed articles in the field.

*Émile Tompa* is a senior scientist, PhD, at the Institute for Work & Health, Toronto, Canada. Émile is a labour and health economist and his research interests include the consequences of occupational health and safety system design on the health and well‐being of individuals and populations and the economic evaluation of workplace interventions for improving the health and well‐being of workers. Émile has authored numerous peer‐reviewed articles in the field of occupational health and safety, including systematic reviews.

**EGM methods:**

*Trine Filges, Anja Bondebjerg, and Malene Wallach Kildemoes*

*Trine Filges* holds a PhD in Economics and has extensive experience as a systematic reviewer and methodologist, having completed a number of systematic reviews in social welfare topic areas as well as in the field of education. Trine has published a number of Campbell Systematic reviews as well as systematic and meta‐analytic reviews in high‐impact journals and is also currently authoring or co‐authoring a number of other Campbell systematic reviews. Trine's fields of expertise are systematic review methods and statistical analysis.

*Anja Bondebjerg* holds a Master's degree in Sociology and has worked extensively with systematic reviews and research mappings, primarily in the fields of education and early childhood education and care. Anja's field of expertise is systematic review methods. Anja has been the author or co‐author of several Campbell systematic reviews and has also co‐authored a qualitative review published as a journal article. Anja is currently co‐authoring a number of other Campbell systematic reviews.

*Malene Wallach Kildemoes* holds a Master's degree in public health and has worked with Campbell reviews for several years. For the present EGM, Malene has contributed to both screening, data extraction, AMSTAR‐appraisal, map construction, and writing of the EGM report.

**Information retrieval:**

*Elizabeth Bengtsen*

*Elizabeth Bengtsen* (information specialist) is an experienced research librarian and search specialist who has worked for core research institutions in Denmark for many years, including The National Research Centre for the Working Environment. With her experience and expertise, Elizabeth contributed to this EGM both with her information retrieval skills, her profound insight into the research field, and her familiarity with systematic review methodology.

## DECLARATIONS OF INTEREST

None of the authors hold any conflicts of interest in this EGM.

## PLANS FOR UPDATING THE EGM

Anja Bondebjerg and Trine Filges will be responsible for regular updates of the EGM, but this is subject to financing being available.

## DIFFERENCES BETWEEN PROTOCOL AND REVIEW

We had planned to perform seaches in ProQuest Dissertations & Theses Global (ProQuest), Conference Proceedings Citation Index, and Index of Conference Proceedings, but were unable to do so due to lack of access. We believe nonetheless that our other searches were comprehensive enough to secure adequate coverage of both dissertations and conference proceedings. Worth noting is also that several of the databases included in the literature search (Academic Search Premier, CINAHL, PubMed, Science Citation Index Expanded [Web Of Science], and Social Sciences Citation Index [Web Of Science]) already include conference proceedings.

In the protocol, we planned to perform citation‐tracking by identifying the most recently published relevant studies for citation‐tracking after the title/abstract screening, and to perform citation analysis of the most cited studies in Google Scholar and Web of Science. After performing the searches, we found it more meaningful to follow an approach where we citation‐tracked all included primary studies, systematic reviews, and ongoing studies, and additionally went through the reference lists of several excluded studies that we judged to possibly contain relevant references. Furthermore, we performed citation‐analysis for the included systematic reviews in Google Scholar and Web of Science as planned at the protocol stage.

Also regarding the literature search, we made a change to our approach to contacting experts. We had initially planned to primarily contact authors of reviews/gap maps, as well as authors with many publications on their curriculum, and/or authors of newly published works. However, we found it more sensible to draw on the international experts already involved in the project (Hermann Burr, Peter Hasle, and Émile Tompa) who we knew to have both extensive content knowledge and large research portfolios. Furthermore, we enlisted the help of our stakeholders in pointing us in the direction of potentially relevant studies that had not already been identified in our searches.

Finally, we found it relevant to introduce one more addition to the AMSTAR‐2 tool on top of the two already detailed in the protocol. Hence, we used the full AMSTAR‐2 tool with the following three additions: (1) assessment of whether the authors present a plan for handling dependent effect sizes, (2) assessment of whether risk of bias assessments of included studies are performed in duplicate, that is, performed independently by two reviewers after which a consensus coding is agreed upon, and (3) assessment of the authors’ investigation of causes for heterogeneity under the domain covering statistical combination of results in the meta‐analysis.

## PUBLISHED NOTES

**Characteristics of studies**

**Characteristics of included studies**

**Table:** 

Agnesi (2016)
**Notes**
Risk of bias table
Alwall (2020)
**Notes**
Risk of bias table
Andersen (2019)
**Notes**
Risk of bias table
Björkdahl (2008)
**Notes**
Risk of bias table
Cashman (2009)
**Notes**
Risk of bias table
Chen (2008)
**Notes**
Risk of bias table
Dahl (2013)
**Notes**
Risk of bias table
Dahl (2022)
**Notes**
Risk of bias table
Dyreborg (2022)
**Notes**
Risk of bias table
Foley (2012)
**Notes**
Risk of bias table
Hogg‐Johnson (2012)
**Notes**
Risk of bias table
Johnson (2019)
**Notes**
Risk of bias table
Kemmlert (1996)
**Notes**
Risk of bias table
Kim (2021)
**Notes**
Risk of bias table
Lewchuk (1996)
**Notes**
Risk of bias table
López‐Ruiz (2013)
**Notes**
Risk of bias table
Macpherson (2022)
**Notes**
Risk of bias table
Mancini (2005)
**Notes**
Risk of bias table
McCaffrey (1983)
**Notes**
Risk of bias table
McLeod (2018)
**Notes**
Risk of bias table
McLeod (2019)
**Notes**
Risk of bias table
Mischke (2013)
**Notes**
Risk of bias table
Moran (1985)
**Notes**
Risk of bias table
Nelson (1997)
**Notes**
Risk of bias table
Peto (2016)
**Notes**
Risk of bias table
Rautiainen (2008)
**Notes**
Risk of bias table
Rubio‐Romero (2015)
**Notes**
Risk of bias table
Ruser (1991)
**Notes**
Risk of bias table
Smith (1979)
**Notes**
Risk of bias table
Soriano‐Serrano (2020)
**Notes**
Risk of bias table
Stocks (2015)
**Notes**
Risk of bias table
van der Molen (2018)
**Notes**
Risk of bias table
Weil (1996)
**Notes**
Risk of bias table
Wickizer (2004)
**Notes**
Risk of bias table
Footnotes
Characteristics of excluded studies
Arocena (2009)
**Reason for exclusion**
Baggs (2001)
**Reason for exclusion**
Baggs (2003)
**Reason for exclusion**
Burstyn (2010)
**Reason for exclusion**
Carpenter (2009)
**Reason for exclusion**
Cooke (1981)
**Reason for exclusion**
Farina (2016)
**Reason for exclusion**
Feltner (2016)
**Reason for exclusion**
Gray (1991)
**Reason for exclusion**
Gray (1991a)
**Reason for exclusion**
Gray (1993)
**Reason for exclusion**
Gray (2005)
**Reason for exclusion**
Haviland (2010)
**Reason for exclusion**
Haviland (2012)
**Reason for exclusion**
Imamura (2018)
**Reason for exclusion**
Johannessen (2017)
**Reason for exclusion**
Ko (2010)
**Reason for exclusion**
Lee (2022)
**Reason for exclusion**
Li (2019)
**Reason for exclusion**
Mendeloff (2005a)
**Reason for exclusion**
Mendeloff (2005b)
**Reason for exclusion**
Parker (2016)
**Reason for exclusion**
Peek‐Asa (2004)
**Reason for exclusion**
Scholz (1990)
**Reason for exclusion**
Smitha (2001)
**Reason for exclusion**
Tompa (2007)
**Reason for exclusion**
Tompa (2016)
**Reason for exclusion**

**Characteristics of ongoing studies**

**Table:** 

Abildgaard (2021)
**Study name**
**Starting date**
**Contact information**
**Notes**
Indregard (2019)
**Study name**
**Starting date**
**Contact information**
**Notes**
Sundstrup (2022)
**Study name**
**Starting date**
**Contact information**
**Notes**

## SOURCES OF SUPPORT

**Internal sources**

- VIVE, The Danish Center for Social Science Research, Denmark

**External sources**

- No sources of support provided

## Supporting information

Supporting information.

Supporting information.

## Footnotes

^1^

http://www.prisma-statement.org/PRISMAStatement/. Accessed January 7, 2021.

^2^
We have used the AMSTAR‐2 tool with three additions, see ‘Tools for assessing risk of bias/study quality of included reviews’.

^3^
See, for example, Hawkins (1989).
