# Supplementary material for: Occupational health and safety regulatory interventions to improve the work environment: An evidence and gap map of effectiveness studies
Source: Campbell Syst Rev. 2023 Dec 11;19(4):e1371. doi: 10.1002/cl2.1371 (PMC10712440; doi:10.1002/cl2.1371)
Supplement: Supplementary file 1 — Supporting information. [file CL2-19-e1371-s001.docx]

Appendices

1 Justification of exclusion of studies using an instrumental variable (IV) approach

Studies using instrument variables (IV) for causal inference in non-randomised studies were not included since the interpretation of IV estimates is challenging. IV only provides an estimate for a specific group, namely people whose behaviour changes due to changes in the particular instrument used. It is not informative about effects on never-takers and always-takers because the instrument does not affect their treatment status. The estimated effect is thus applicable only to the subpopulation whose treatment status is affected by the instrument. As a consequence, the effects differ for different IVs and care has to be taken as to whether they provide useful information. The effect is interesting when the instrument it is based on is interesting, in the sense that it corresponds to a policy instrument of interest. Further, if those that are affected by the instrument are not affected in the same way, the IV estimate is an average of the impacts of changing treatment status in both directions and cannot be interpreted as a treatment effect. To turn the IV estimate into a LATE requires a monotonicity assumption. The movements induced by the instrument go in one direction only, from no treatment to treatment. The IV estimate, interpreted as a LATE, is only applicable to the complier population, those that are affected by the instrument in the ‘right way’. It is not possible to characterise the complier population, as an observation’s subpopulation cannot be determined and defiers do not exist by assumption.

In the binary-treatment–binary-instrument context, the IV estimate can, given monotonicity, be interpreted as a LATE; i.e. the average treatment effect for the subpopulation of compliers. If treatment or instruments are not binary, interpretation becomes more complicated. In the binary-treatment–multivalued-instrument (ordered to take values from 0 to *J*) context, the IV estimate, given monotonicity, is a weighted average of pairwise LATE parameters (comparing subgroup *j* with subgroup *j*−1). The IV estimate can thus be interpreted as the weighted average of average treatment effects in each of the *J* subgroups of compliers. In the multivalued-treatment (ordered to take values from 0 to *T*) – multivalued-instrument (ordered to take values from 0 to *J*) context, the IV estimate for *each pair of instrument values*, given monotonicity, is a weighted average of the effects from going from *t*-1 to *t* for persons induced by the change in the value of the instrument to move from any level below *t* to the level *t* or any level above. Persons can be counted multiple times in forming the weights.

Bibliography:

- Angrist, J.D., & Pischke, J.S. (2009*). Mostly Harmless Econometrics: An Empiricist’s Companion.* Princeton, NJ: Princeton University Press.
- Heckman, J.J. & Urzúa, S. (2010). Comparing IV with structural models: What simple IV can and cannot identify. *Journal of Econometrics, 156*, 27-37.
- Heckman, J.J., Urzúa, S. & Vytlacil, E. (2006). Understanding instrumental variables in models with essential heterogeneity. *The Review of Economics* *and Statistics, 88*(3), 389-432.

## 2 Database searches with results

Database searches were performed in January 2022 and the search strings and results for each database are shown below.

**Academic Search Premier** (1931 – 2022). Search date: 03.01.2022 through the EBSCO platform. Expanders - Apply equivalent subjects. Search modes - Boolean/Phrase.

| **#** | **Query** | **Results** |
| --- | --- | --- |
| S18 | S6 AND S13 AND S17 | 12,213 |
| S17 | S14 OR S15 OR S16 | 8,513,426 |
| S16 | (((((DE “EFFECT sizes (Statistics)”) OR (DE “CONTROL groups”)) OR (DE “EXPERIMENTAL groups”)) OR (DE “EXPERIMENTS”)) OR (DE “MATCHED groups”)) OR (DE “RANDOMIZED controlled trials”) | 140,593 |
| S15 | AB ( (((control* OR difference* OR matched* OR random* OR reference* OR compare* OR longitudinal OR cohort*) N3 (group* OR trial* OR test* OR study OR studies OR analy*))) OR AB ( intervent* OR experiment* OR impact*) OR AB ( “systematic review” OR “meta analy*“ OR metaanaly* OR “meta-analy*“) OR AB ( “gap map” OR “follow-up stud*“ OR “follow up stud*“ OR “followup stud*“)) | 5,722,120 |
| S14 | TI (control* OR difference* OR matched* OR random* OR reference* OR compare* OR group* OR trial* OR test* OR intervent* OR experiment* OR impact* OR “systematic review” OR “meta analy*“ OR metaanaly* OR “meta-analy*“ OR “gap map” OR study OR studies OR analy* OR longitudinal OR “follow-up stud*“ OR “follow up stud*“ OR “followup stud*“ OR cohort*) | 4,308,755 |
| S13 | S7 OR S8 OR S9 OR S10 OR S11 OR S12 | 3,561,276 |
| S12 | KW ( ((occupational N3 (health OR safety))) | 14,907 |
| S11 | SU ( ((occupational N3 (health OR safety))) OR SU ( ((work* N3 (health OR safety))) | 14,490 |
| S10 | TI ( ((occupation* N3 (health OR safety))) OR TI ( ((work* N3 (health OR safety))) | 19,668 |
| S9 | DE “INDUSTRIAL hygiene” | 20,748 |
| S8 | AB (incentive* OR fund* OR subsid* OR recogni* OR award* OR inspect* OR audit* OR consult* OR sanction* OR penalt* OR fine* OR prosecution* OR “enforceable undertaking*“ OR “order to comply” OR citation* OR notification* OR violation* OR breach* OR “regulation” OR enforce* OR ((information* OR awareness OR training) N3 (campaign* OR initiative* OR program*))) | 3,028,786 |
| S7 | TI ( incentive* OR fund* OR subsid* OR recogni* OR award* OR inspect* OR audit* OR consult* OR sanction* OR penalt* OR fine# OR prosecution* OR citation* OR notification* OR violation* OR breach* OR “enforceable undertaking*“ OR TI “order to comply” OR information* OR awareness OR training OR regulation OR enforce*) | 891,375 |
| S6 | S3 OR S4 OR S5 | 144,754 |
| S5 | (((DE “WORK environment”) OR (DE “WORK environment -- Psychological aspects” OR DE “WORK environment -- Research” OR DE “WORK environment -- Social aspects”)) OR (DE “ERGONOMICS”) | 44,606 |
| S4 | AB ((work* OR company OR companies* OR firm OR firms OR organization* OR organisation* OR business* OR institut* OR employe* OR worker* OR staff* OR job) N5 (environ* OR occupational)) | 103,131 |
| S3 | (S1 AND S2) | 13,467 |
| S2 | TI (environ* OR occupation*) | 278,763 |
| S1 | TI (work* OR company OR companies OR firm OR firms OR organization* or organisation* OR business* OR institut* OR employe* OR worker* OR staff* OR job) | 807,993 |

**EconLit** (1969 – 2022). Search date: 04.01.2022 through the EBSCO platform. Expanders - Apply equivalent subjects. Search modes - Boolean/Phrase.

| **#** | **Query** | **Results** |
| --- | --- | --- |
| S15 | (S4 AND S10 AND S14) | 2,198 |
| S14 | S11 OR S12 OR S13 | 323,320 |
| S13 | SU (“effect size” OR “control groups” OR “experimental groups” OR experiments OR “matched groups” OR “quasiexperimental design” OR “randomized controlled trials” OR “comparative testing”) | 10,198 |
| S12 | AB (((control* OR difference* OR matched* OR random* OR reference* OR compare* OR longitudinal OR cohort*) N3 (group* OR trial* OR test* OR study OR studies OR analy*)) OR intervent* OR experiment* OR impact* OR “systematic review” OR “meta analy*“ OR metaanaly* OR “meta-analy* OR “gap map” OR “follow-up stud*“ OR “follow up stud*“ OR “followup stud*“) | 236,180 |
| S11 | TI (control* OR difference* OR matched* OR random* OR reference* OR compare* OR group* OR trial* OR test* OR intervent* OR experiment* OR impact* OR “systematic review” OR “meta analy*“ OR metaanaly* OR “meta-analy* OR “gap map” OR study OR studies OR analy* OR longitudinal OR “follow-up stud*“ OR “follow up stud*“ OR “followup stud*“ OR cohort*) | 135,825 |
| S10 | S5 OR S6 OR S7 OR S8 OR S9 | 356,634 |
| S9 | SU ((occupation* N3 (health OR safety)) OR SU ((work* N3 (health OR safety)) | 312 |
| S8 | TI ((occupation* N3 (health OR safety)) OR TI ((work* N3 (health OR safety)) | 966 |
| S7 | SU industrial hygiene | 33 |
| S6 | AB ( (incentive* OR fund* OR subsid* OR recogni* OR award* OR inspect* OR audit* OR consult* OR sanction* OR penalt* OR fine* OR prosecution* OR citation* OR notification* OR violation* OR breach* OR “enforceable undertaking*“ OR “order to comply” OR information* OR awareness OR training OR injunction* OR enforcement* OR regulat* OR legislation)) OR AB ( ((information* OR awareness OR training) N3 (campaign* OR initiative* OR program*)))) | 317,707 |
| S5 | TI (incentive* OR fund* OR subsid* OR recogni* OR award* OR inspect* OR audit* OR consult* OR sanction* OR penalt* OR fine* OR prosecution* OR citation* OR notification* OR violation* OR breach* OR “enforceable undertaking*“ OR “order to comply” OR information* OR awareness OR training OR regulation OR enforce*) | 95,381 |
| S4 | S1 OR S2 OR S3 | 16,879 |
| S3 | (ZW “work”) or (ZW “working environment”) or (ZW “working environments”) | 17 |
| S2 | AB ((work* OR company OR companies* OR firm OR firms OR organizaton* OR organisation* OR business* OR institut* OR employe* OR worker* OR staff* OR job) N5 (environ* OR occupational)) | 14,710 |
| S1 | TI ( (work* OR company OR companies* OR firm OR firms OR organizaton* OR organisation* OR business* OR institut* OR employe* OR worker* OR staff* OR job)) AND TI ( environ* OR occupation*)) | 3,384 |

**PsycINFO** (1890 – 2022)**.** Search date: 04.01.2022 through the EBSCO platform. Expanders - Apply equivalent subjects. Search modes - Boolean/Phrase.

| **#** | **Query** | **Results** |
| --- | --- | --- |
| S16 | S6 AND S11 AND S15 | 8,104 |
| S15 | S12 OR S13 OR S14 | 2,078,761 |
| S14 | (((DE “Effect Size (Statistical)”) OR (DE “Experiment Controls”)) OR (DE “Quasi Experimental Methods”)) OR (DE “Randomized Controlled Trials”) | 3,806 |
| S13 | AB (((control* OR difference* OR matched* OR random* OR reference* OR compare* OR longitudinal OR cohort*) N3 (group* OR trial* OR test* OR study OR studies OR analy*)) OR intervent* OR experiment* OR impact* OR “systematic review” OR “meta analy*“ OR metaanaly* OR “meta-analy* OR “gap map” OR “follow-up stud*“ OR “follow up stud*“ OR “followup stud*“) | 1,478,799 |
| S12 | TI (control* OR difference* OR matched* OR random* OR reference* OR compare* OR group* OR trial* OR test* OR intervent* OR experiment* OR impact* OR “systematic review” OR “meta analy*“ OR metaanaly* OR “meta-analy*“ OR “gap map” OR study OR studies OR analy* OR longitudinal OR “follow-up stud*“ OR “follow up stud*“ OR “followup stud*“ OR cohort*) | 1,053,558 |
| S11 | S7 OR S8 OR S9 OR S10 | 794,024 |
| S10 | KW ((occupation* N3 (health OR safety)) OR KW ((work* N3 (health OR safety)) | 7,937 |
| S9 | TI ((occupation* N3 (health OR safety)) OR TI ((work* N3 (health OR safety)) | 7,769 |
| S8 | AB ( (incentive* OR fund* OR subsid* OR recogni* OR award* OR inspect* OR audit* OR consult* OR sanction* OR penalt* OR fine* OR prosecution* OR citation* OR notification* OR violation* OR breach* OR “enforceable undertaking*“ OR “order to comply” OR regulation OR enforce*)) OR AB ( ((information* OR awareness OR training) N3 (campaign* OR initiative* OR program*)))) | 660,362 |
| S7 | TI (incentive* OR fund* OR subsid* OR recogni* OR award* OR inspect* OR audit* OR consult* OR sanction* OR penalt* OR fine* OR prosecution* OR citation* OR notification* OR violation* OR breach* OR “enforceable undertaking*“ OR “order to comply” OR information* OR awareness OR training OR regulation OR enforce*) | 243,099 |
| S6 | S1 OR S2 OR S3 OR S4 OR S5 | 76,221 |
| S5 | DE “Occupational Health” | 6,511 |
| S4 | KW “work environment” OR “work environments” OR “working conditions” OR “working condition” | 5,844 |
| S3 | (DE “Occupational Health Psychology” OR DE “Working Conditions”) OR (DE “Human Factors Engineering”) | 33,144 |
| S2 | AB ((work* OR company OR companies* OR firm OR firms OR organization* OR organisation* OR business* OR institut* OR employe* OR worker* OR staff* OR job) N5 (environ* OR occupational)) | 43,279 |
| S1 | TI ( (work* OR company OR companies* OR firm OR firms OR organization* OR organisation* OR business* OR institut* OR employe* OR worker* OR staff* OR job)) AND TI ( environ* OR occupation*)) | 8,032 |

**SocINDEX** (1895 – 2022). Search date: 04.01.2022 through the EBSCO platform. Expanders - Apply equivalent subjects. Search modes - Boolean/Phrase.

| **#** | **Query** | **Results** |
| --- | --- | --- |
| S15 | S4 AND S10 AND S14 | 2,215 |
| S14 | S11 OR S12 OR S13 | 573,592 |
| S13 | DE (“effect size” OR “Control Groups” OR “Experimental Groups” OR “Experiments” OR “Matched Groups” OR “Quasiexperimental Design” OR “Randomized Controlled Trials” OR “Comparative Testing”) | 3,823 |
| S12 | AB ( ((control* OR difference* OR matched* OR random* OR reference* OR compare* OR longitudinal OR cohort*) N3 (group* OR trial* OR test* OR study OR studies OR analy*))) OR AB ( (intervent* OR experiment* OR impact* OR “systematic review” OR “meta analy*“ OR metaanaly* OR meta-analy* OR “gap map” OR “follow-up stud*“ OR “follow up stud*“ OR “followup stud*“)) | 355,700 |
| S11 | TI (control* OR difference* OR matched* OR random* OR reference* OR compare* OR group* OR trial* OR test* OR intervent* OR experiment* OR impact* OR “systematic review” OR “meta analy*“ OR metaanaly* OR meta-analy* OR “gap map” OR study OR studies OR analy* OR longitudinal OR “follow-up stud*“ OR “follow up stud*“ OR “followup stud*“ OR cohort*) | 313,131 |
| S10 | S5 OR S6 OR S7 OR S8 OR S9 | 370,128 |
| S9 | SU ( (occupational N3 (health OR safety))) OR SU ( (work* N3 (health OR safety))) | 1,900 |
| S8 | TI ( (occupation* N3 (health OR safety))) OR TI ( (work* N3 (health OR safety))) | 4,316 |
| S7 | DE “INDUSTRIAL hygiene” | 2,248 |
| S6 | AB ( (incentive* OR fund* OR subsid* OR recogni* OR award* OR inspect* OR audit* OR consult* OR sanction* OR penalt* OR fine* OR prosecution* OR “enforceable undertaking*“ OR “order to comply” OR citation* OR notification* OR violation* OR breach* OR enforce* OR regulation)) OR AB ( ((information* OR awareness OR training) N3 (campaign* OR initiative* OR program*))) | 323,803 |
| S5 | TI (incentive* OR fund* OR subsid* OR recogni* OR award* OR inspect* OR audit* OR consult* OR sanction* OR penalt* OR fine* OR prosecution* OR citation* OR notification* OR violation* OR breach* OR “enforceable undertaking*“ OR “order to comply” OR information* OR awareness OR training OR enforce* OR regulation) | 85,333 |
| S4 | S1 OR S2 OR S3 | 29,362 |
| S3 | (((DE “WORK environment”) OR (DE “WORK environment -- Psychological aspects” OR DE “WORK environment -- Research”)) OR (DE “ERGONOMICS”)) OR (DE “WORK environment -- Social aspects”) | 11,256 |
| S2 | AB ((work* OR company OR companies* OR firm OR firms OR organization* OR organisation* OR business* OR institut* OR employe* OR worker* OR staff* OR job) N5 (environ* OR occupational)) | 18,422 |
| S1 | TI ( work* OR company OR companies* OR firm OR firms OR organization* OR organisation* OR business* OR institut* OR employe* OR worker* OR staff* OR job)) AND TI ( (environ* OR occupation*)) | 3,912 |

**CINAHL** (1981 - 2022). Search date: 03.01.2022 through the EBSCO platform. Expanders - Apply equivalent subjects. Search modes - Boolean/Phrase.

| **#** | **Query** | **Results** |
| --- | --- | --- |
| S21 | S5 AND S11 AND S20 | 5,982 |
| S20 | S12 OR S13 OR S14 OR S15 OR S16 OR S17 OR S18 OR S19 | 2,216,937 |
| S19 | “comparative testing” | 35 |
| S18 | (MH “Randomized Controlled Trials”) | 122,668 |
| S17 | “matched group” | 1,033 |
| S16 | (MH “Experimental Studies”) OR (MH “Quasi-Experimental Studies”) | 40,402 |
| S15 | (MH “Control Group”) | 12,716 |
| S14 | (MH “Effect Size”) | 20,096 |
| S13 | AB (((control* OR difference* OR matched* OR random* OR reference* OR compare* OR longitudinal OR cohort*) N3 (group* OR trial* OR test* OR study OR studies OR analy*)) OR intervent* OR experiment* OR impact* OR “systematic review” OR “meta analy*“ OR metaanaly* OR “meta-analy* OR “gap map” OR “follow-up stud*“ OR “follow up stud*“ OR “followup stud*“) | 1,346,200 |
| S12 | TI (control* OR difference* OR matched* OR random* OR reference* OR compare* OR group* OR trial* OR test* OR intervent* OR experiment* OR impact* OR “systematic review” OR “meta analy*“ OR metaanaly* OR “meta-analy*“ OR “gap map” OR study OR studies OR analy* OR longitudinal OR “follow-up stud*“ OR “follow up stud*“ OR “followup stud*“ OR cohort*) | 1,340,258 |
| S11 | S6 OR S7 OR S8 OR S9 OR S10 | 750,203 |
| S10 | SU ((occupation* N3 (health OR safety)) OR SU ((work* N2 (health OR safety)) | 67,439 |
| S9 | TI ((occupation* N3 (health OR safety)) OR TI ((work* N2 (health OR safety)) | 18,602 |
| S8 | (MH “Occupational Health”) | 28,322 |
| S7 | AB ( (incentive* OR fund* OR subsid* OR recogni* OR award* OR inspect* OR audit* OR consult* OR sanction* OR penalt* OR fine* OR prosecution* OR citation* OR notification* OR violation* OR breach* OR “enforceable undertaking*“ OR “order to comply” OR regulation OR enforce*) OR AB ( ((information* OR awareness OR training) N3 (campaign* OR initiative* OR program*)))) | 473,187 |
| S6 | TI (incentive* OR fund* OR subsid* OR recogni* OR award* OR inspect* OR audit* OR consult* OR sanction* OR penalt* OR fine* OR prosecution* OR citation* OR notification* OR violation* OR breach* OR “enforceable undertaking*“ OR “order to comply” OR information* OR awareness OR training OR regulation OR enforce*) | 274,733 |
| S5 | S1 OR S2 OR S3 OR S4 | 45,212 |
| S4 | (MH “Ergonomics”) | 8,446 |
| S3 | (MH “Work Environment+/LJ/PF/ST”) | 2,840 |
| S2 | AB ((work* OR company OR companies* OR firm OR firms OR organization* OR organisation* OR business* OR institut* OR employe* OR worker* OR staff* OR job) N5 (environ* OR occupational)) | 30,058 |
| S1 | TI ( (work* OR company OR companies* OR firm OR firms OR organization* OR organisation* OR business* OR institut* OR employe* OR worker* OR staff* OR job)) AND TI ( environ* OR occupation*)) | 8,455 |

**International Bibliography of the Social Sciences (IBSS)** (1951 – 2022), **Sociological Abstracts** (1952 – 2022), and **Social Services Abstracts** (1979 – 2022). Search date: 04.01.2022 through the ProQuest platform. Search modes - Boolean/Phrase.

**#1** ti(( work* OR company OR companies* OR firm OR firms OR organization* OR organisation* OR business* OR institut* OR employe* OR worker* OR staff* OR job)) AND ti((environ* OR occupation*)) 8,607

**#2** ab((((work* OR company OR companies* OR firm OR firms OR organization* OR organisation* OR business* OR institut* OR employe* OR worker* OR staff* OR job) NEAR/3 (environ* OR occupational*)))) 28,726

**#3** MAINSUBJECT.EXACT(“Work environment”) 13,096

**#4** (#1 OR #2 OR #3) 44,271

**#5** ti(incentive* OR fund* OR subsid* OR recogni* OR award* OR inspect* OR audit* OR consult* OR sanction* OR penalt* OR fine* OR prosecution* OR citation* OR notification* OR violation* OR breach* OR “enforceable undertaking*“ OR “order to comply” OR information* OR awareness OR training OR regulation OR enforce*) 205,234

**#6** ab((incentive* OR funding* OR subsid* OR recogni* OR award* OR inspect* OR audit* OR consult* OR sanction* OR penalt* OR fine* OR prosecution* OR “enforceable undertaking*“ OR “order to comply” OR citation* OR notification* OR violation* OR breach* OR regulation OR enforce*)) OR ab(((information* OR awareness OR training) N/2 (campaign* OR initiative* OR program*))) 493,079

**#7** MAINSUBJECT.EXACT(“Occupational safety”) OR

MAINSUBJECT.EXACT(“Occupational Safety and Health”) 4,861

**#8** ti((occupation* NEAR/3 (health OR safety))) OR

ti((work* NEAR/3 (health OR safety))) 7,466

**#9** su((occupation* NEAR/3 (health OR safety))) OR

su((work* NEAR/3 (health OR safety))) 12,355

**#10** (#5 OR #6 OR #7 OR #8 OR #9) 639,355

**#11** ti(control* OR difference* OR matched* OR random* OR reference* OR compare* OR group* OR trial* OR test* OR intervent* OR experiment* OR impact* OR “systematic review” OR “meta analy*“ OR metaanaly* OR “meta-analy*“ OR “gap map” OR study OR studies OR analy* OR longitudinal OR “follow-up stud*“ OR “follow up stud*“ “followup stud*“ OR cohort*) 844,966

**#12** ab(((control* OR difference* OR matched* OR random* OR reference* OR compare* OR longitudinal OR cohort*) N/3 (group* OR trial* OR test* OR study OR studies OR analy*))) OR ab((intervent* OR experiment* OR impact* OR “systematic review” OR “meta analy*“ OR metaanaly* OR “meta-analy*“ OR “gap map” OR “follow-up stud*“ OR “follow up stud*“ OR “followup stud*“)) 737,499

**#13** mainsubject.Exact(“comparative analysis” OR “research design” OR “design of experiments”) OR su(“effect size” OR “quasiexperimental” OR “quasi-experimental” OR “randomized controlled trials” OR “comparative testing”) 78,252

**#14** (#11 OR #12 OR #13) 1,427,492

**#15** (#4 AND #10 AND #15) 3,352

**Social Citation Index Expanded** and **Social Sciences Citation Index**, see Figure 2.

**MEDLINE/PubMed** (1950 – 2022). Search date: 02.01.2022 through the PubMed interface. Search modes - Boolean/Phrase.

| **Search** | **Query** | **Results** |
| --- | --- | --- |
| #18 | Search: **#3 AND #10 AND #17** Sort by: **Most Recent** | 39,805 |
| #17 | Search: **#11 OR #12 OR #13 OR #16** Sort by: **Most Recent** | 12,637,471 |
| #16 | Search: **#14 OR #15** Sort by: **Most Recent** | 710,908 |
| #15 | Search: **“effect size”[Title/Abstract] OR “experimental groups”[Title/Abstract] OR “matched groups”[Title/Abstract] OR “quasi-experimental”[Title/Abstract] OR “quasiexperimental”[Title/Abstract] OR “randomized controlled trials”[Title/Abstract] OR “comparative testing”[Title/Abstract]** Sort by: **Most Recent** | 150,910 |
| #14 | Search: **((control groups[MeSH Terms]) OR (research design[MeSH Terms])) OR (randomized controlled trials as topic[MeSH Terms])** Sort by: **Most Recent** | 599,485 |
| #13 | Search: **intervention*[Title/Abstract] OR “experimental”[Title/Abstract] OR “experiment”[Title/Abstract] OR “experiments”[Title/Abstract] OR impact*[Title/Abstract] OR “systematic review”[Title/Abstract] OR “meta-analy*“[Title/Abstract] OR metaanaly*[Title/Abstract] OR “gap map*“[Title/Abstract] OR “follow-up stud*“[Title/Abstract] OR “followup stud*“[Title/Abstract]** Sort by: **Most Recent** | 4,669,937 |
| #12 | Search: **(control*[Title/Abstract] OR difference*[Title/Abstract] OR “matched”[Title/Abstract] OR random*[Title/Abstract] OR reference*[Title/Abstract] OR compare*[Title/Abstract] OR “longitudinal”[Title/Abstract] OR cohort*[Title/Abstract]) AND (group*[Title/Abstract] OR trial*[Title/Abstract] OR test*[Title/Abstract] OR “study”[Title/Abstract] OR “studies”[Title/Abstract] OR analy*[Title/Abstract])** Sort by: **Most Recent** | 7,607,822 |
| #11 | Search: **control*[Title] OR difference*[Title] OR matched*[Title] OR random*[Title] OR reference*[Title] OR compare*[Title] OR group*[Title] OR trial*[Title] OR test*[Title] OR intervent*[Title] OR experiment*[Title] OR impact*[Title] OR “systematic review”[Title] OR “meta-analy*“[Title] OR “metaanaly*“[Title] OR “gap map*“[Title] OR “longitudinal”[Title] OR “follow-up stud*“[Title] OR “followup stud*“[Title] OR “study”[Title] OR “studies”[Title] OR analy*[Title] OR cohort*[Title]** Sort by: **Most Recent** | 5,264,938 |
| #10 | Search: **#4 OR #5 OR #6 OR #7 OR #8 OR #9** Sort by: **Most Recent** | 2,389,860 |
| #9 | Search: **(work*[Title]) AND (health[Title] OR safety[Title])** Sort by: **Most Recent** | 36,635 |
| #8 | Search: **(occupation*[Title]) AND (health[Title] OR safety[Title])** Sort by: **Most Recent** | 10,766 |
| #7 | Search: **(occupational health[MeSH Terms])** Sort by: **Most Recent** | 35,579 |
| #6 | Search: **(information*[Title/Abstract] OR awareness[Title/Abstract] OR training[Title/Abstract]) AND (campaign*[Title/Abstract] OR initiative*[Title/Abstract] OR program*[Title/Abstract])** Sort by: **Most Recent** | 259,441 |
| #5 | Search: **incentive*[Title/Abstract] OR “fund”[Title/Abstract] OR “funds”[Title/Abstract] OR “funding”[Title/Abstract] OR subsid*[Title/Abstract] OR “recognition”[Title/Abstract] OR award*[Title/Abstract] OR “inspect”[Title/Abstract] OR “inspection”[Title/Abstract] OR audit*[Title/Abstract] OR consult*[Title/Abstract] OR sanction*[Title/Abstract] OR “penalty”[Title/Abstract] OR “penalties”[Title/Abstract] OR “fines”[Title/Abstract] OR “fined”[Title/Abstract] OR prosecution*[Title/Abstract] OR citation*[Title/Abstract] OR “notification”[Title/Abstract] OR “notifications”[Title/Abstract] OR violation*[Title/Abstract] OR breach*[Title/Abstract] OR enforce*[Title/Abstract] OR “order to comply”[Title/Abstract] OR “regulation”[Title/Abstract]** Sort by: **Most Recent** | 1,838,455 |
| #4 | Search: **incentive*[Title] OR fund*[Title] OR subsid*[Title] OR recogni*[Title] OR award*[Title] OR inspect*[Title] OR audit*[Title] OR consult*[Title] OR sanction[Title] OR penalt*[Title] OR fine*[Title] OR prosecution*[Title] OR citation*[Title] OR notification*[Title] OR violation*[Title] OR breach*[Title] OR enforce*[Title] OR “order to comply”[Title] OR “regulation”[Title] OR information*[Title] OR awareness[Title] OR training[Title]** Sort by: **Most Recent** | 779,040 |
| #3 | Search: **#1 OR #2** Sort by: **Most Recent** | 328,041 |
| #2 | Search: **workplace[MeSH Terms]** Sort by: **Most Recent** | 26,624 |
| #1 | Search: **(work*[Title/Abstract] OR company[Title/Abstract] OR companies[Title/Abstract] OR “firms”[Title/Abstract] OR organization*[Title/Abstract] OR organisation*[Title/Abstract] OR business*[Title/Abstract] OR institut*[Title/Abstract] OR employe*[Title/Abstract] OR worker* OR staff*[Title/Abstract] OR job[Title/Abstract]) AND (environ*[Title/Abstract] OR “occupation*“[Title/Abstract])** Sort by: **Most Recent** | 312,119 |

**ERIC** (1966 – 2022). Search date: 04.01.2022 through the EBSCO platform. Expanders - Apply equivalent subjects. Search modes - Boolean/Phrase.

| **#** | **Query** | **Results** |
| --- | --- | --- |
| S16 | (S12 OR S13 OR S14) AND (S5 AND S11 AND S15) | 2,590 |
| S15 | S12 OR S13 OR S14 | 528,948 |
| S14 | (((DE “Effect Size” OR DE “Control Groups” OR DE “Experimental Groups” OR DE “Experiments” OR DE “Matched Groups”) OR (DE “Quasiexperimental Design”)) OR (DE “Randomized Controlled Trials”)) OR (DE “Comparative Testing”) | 30,041 |
| S13 | AB (((control* OR difference* OR matched* OR random* OR reference* OR compare* OR longitudinal OR cohort*) N3 (group* OR trial* OR test* OR study OR studies OR analy*)) OR intervent* OR experiment* OR impact* OR “systematic review” OR “meta analy*“ OR metaanaly* OR meta-analy* OR “gap map” OR “follow-up stud*“ OR “follow up stud*“ OR “followup stud*“) | 322,963 |
| S12 | TI (control* OR difference* OR matched* OR random* OR reference* OR compare* OR group* OR trial* OR test* OR intervent* OR experiment* OR impact* OR “systematic review” OR “meta analy*“ OR metaanaly* OR meta-analy* OR “gap map” OR study OR studies OR analy* OR longitudinal OR “follow-up stud*“ OR “follow up stud*“ “followup stud*“ OR cohort*) | 297,840 |
| S11 | S6 OR S7 OR S8 OR S9 OR S10 | 362,754 |
| S10 | SU ((occupation* N3 (health OR safety)) OR SU ((work* N3 (health OR safety)) | 8,606 |
| S9 | TI ((occupation* N3 (health OR safety)) OR TI ((work* N3 (health OR safety)) | 1,324 |
| S8 | DE “Occupational Safety and Health” | 1,103 |
| S7 | AB (incentive* OR fund* OR subsid* OR recogni* OR award* OR inspect* OR audit* OR consult* OR sanction* OR penalt* OR fine* OR prosecution* OR “enforceable undertaking*“ OR “order to comply” OR citation* OR notification* OR violation* OR breach* OR regulation OR enforce* OR ((information* OR awareness OR training) N3 (campaign* OR initiative* OR program*))) | 297,446 |
| S6 | TI (incentive* OR fund* OR subsid* OR recogni* OR award* OR inspect* OR audit* OR consult* OR sanction* OR penalt* OR fine* OR prosecution* OR citation* OR notification* OR violation* OR breach* OR “enforceable undertaking*“ OR “order to comply” OR information* OR awareness OR training OR regulation OR enforce*) | 102,382 |
| S5 | S1 OR S2 OR S3 OR S4 | 28,022 |
| S4 | SU “work environment” OR “work environments” or “working conditions” or “working condition” | 10,649 |
| S3 | DE “Work Environment” | 10,645 |
| S2 | AB ((work* OR company OR companies* OR firm OR firms OR organization* OR organisation* OR business* OR institut* OR employe* OR worker* OR staff* OR job) N5 (environ* OR occupational)) | 17,867 |
| S1 | TI ( work* OR company OR companies* OR firm OR firms OR organization* OR organisation* OR business* OR institut* OR employe* OR worker* OR staff* OR job) AND TI ( environ* OR occupation*) | 2,885 |

## 3 Hand searches in journals with results

We performed hand searches in 16 journals focusing on editions published between 01/01/2015 and 31/12/2022. Hand searches were performed between September 2022 and January 2023 in the following journals:

- *Work & Stress*
- *Policy and Practice in Health and Safety*
- *American Journal of Industrial Medicine*
- *Journal of Labor Economics*
- *Journal of Occupational and Environmental Medicine*
- *International Journal of Environmental Health Research*
- *Scandinavian Journal of Work Environment and Health*
- *Safety Science*
- *Regulation and Governance*
- *Work*
- *Accident Analysis & Prevention*
- *Safety and Health at Work*
- *Journal of Industrial Relations*
- *Law and Policy*
- *Industrial Law Journal*
- *Journal of Safety Research*

The table below documents the hand searches with number of hits screened for each edition of each journal. In total, we screened 15.223 hits.

| Journal | Year | Volume(number) | Hits screened |
| --- | --- | --- | --- |
| Work & Stress | 2015 | 29(1) | 5 |
| Work & Stress | 2015 | 29(2) | 6 |
| Work & Stress | 2015 | 29(3) | 6 |
| Work & Stress | 2015 | 29(4) | 5 |
| Work & Stress | 2016 | 30(1) | 6 |
| Work & Stress | 2016 | 30(2) | 5 |
| Work & Stress | 2016 | 30(3) | 5 |
| Work & Stress | 2016 | 30(4) | 5 |
| Work & Stress | 2017 | 31(1) | 5 |
| Work & Stress | 2017 | 31(2) | 5 |
| Work & Stress | 2017 | 31(3) | 5 |
| Work & Stress | 2017 | 31(4) | 5 |
| Work & Stress | 2018 | 32(1) | 5 |
| Work & Stress | 2018 | 32(2) | 5 |
| Work & Stress | 2018 | 32(3) | 6 |
| Work & Stress | 2018 | 32(4) | 5 |
| Work & Stress | 2019 | 33(1) | 5 |
| Work & Stress | 2019 | 33(2) | 7 |
| Work & Stress | 2019 | 33(3) | 5 |
| Work & Stress | 2019 | 33(4) | 5 |
| Work & Stress | 2020 | 34(1) | 5 |
| Work & Stress | 2020 | 34(2) | 5 |
| Work & Stress | 2020 | 34(3) | 6 |
| Work & Stress | 2020 | 34(4) | 5 |
| Work & Stress | 2021 | 35(1) | 6 |
| Work & Stress | 2021 | 35(2) | 5 |
| Work & Stress | 2021 | 35(3) | 5 |
| Work & Stress | 2021 | 35(4) | 5 |
| Work & Stress | 2022 | 36(1) | 6 |
| Work & Stress | 2022 | 36(2) | 6 |
| Work & Stress | 2022 | 36(3) | 5 |
| Work & Stress | 2022 | 36(4) | 4 |
| Scandinavian Journal of Work, Environment and Health | 2015 | 41(1) | 13 |
| Scandinavian Journal of Work, Environment and Health | 2015 | 41(2) | 12 |
| Scandinavian Journal of Work, Environment and Health | 2015 | 41(3) | 13 |
| Scandinavian Journal of Work, Environment and Health | 2015 | 41(4) | 13 |
| Scandinavian Journal of Work, Environment and Health | 2015 | 41(5) | 11 |
| Scandinavian Journal of Work, Environment and Health | 2015 | 41(6) | 10 |
| Scandinavian Journal of Work, Environment and Health | 2016 | 42(1) | 14 |
| Scandinavian Journal of Work, Environment and Health | 2016 | 42(2) | 9 |
| Scandinavian Journal of Work, Environment and Health | 2016 | 42(3) | 10 |
| Scandinavian Journal of Work, Environment and Health | 2016 | 42(4) | 11 |
| Scandinavian Journal of Work, Environment and Health | 2016 | 42(5) | 10 |
| Scandinavian Journal of Work, Environment and Health | 2016 | 42(6) | 13 |
| Scandinavian Journal of Work, Environment and Health | 2017 | 43(1) | 13 |
| Scandinavian Journal of Work, Environment and Health | 2017 | 43(2) | 12 |
| Scandinavian Journal of Work, Environment and Health | 2017 | 43(3) | 13 |
| Scandinavian Journal of Work, Environment and Health | 2017 | 43(4) | 12 |
| Scandinavian Journal of Work, Environment and Health | 2017 | 43(5) | 13 |
| Scandinavian Journal of Work, Environment and Health | 2017 | 43(6) | Volume does not exist online |
| Scandinavian Journal of Work, Environment and Health | 2018 | 44(1) | 13 |
| Scandinavian Journal of Work, Environment and Health | 2018 | 44(2) | 13 |
| Scandinavian Journal of Work, Environment and Health | 2018 | 44(3) | 13 |
| Scandinavian Journal of Work, Environment and Health | 2018 | 44(4) | 13 |
| Scandinavian Journal of Work, Environment and Health | 2018 | 44(5) | 12 |
| Scandinavian Journal of Work, Environment and Health | 2018 | 44(6) | 12 |
| Scandinavian Journal of Work, Environment and Health | 2019 | 45(1) | 12 |
| Scandinavian Journal of Work, Environment and Health | 2019 | 45(2) | 14 |
| Scandinavian Journal of Work, Environment and Health | 2019 | 45(3) | 14 |
| Scandinavian Journal of Work, Environment and Health | 2019 | 45(4) | 13 |
| Scandinavian Journal of Work, Environment and Health | 2019 | 45(5) | 13 |
| Scandinavian Journal of Work, Environment and Health | 2019 | 45(6) | 12 |
| Scandinavian Journal of Work, Environment and Health | 2020 | 46(1) | 13 |
| Scandinavian Journal of Work, Environment and Health | 2020 | 46(2) | 12 |
| Scandinavian Journal of Work, Environment and Health | 2020 | 46(3) | 12 |
| Scandinavian Journal of Work, Environment and Health | 2020 | 46(4) | 14 |
| Scandinavian Journal of Work, Environment and Health | 2020 | 46(5) | 13 |
| Scandinavian Journal of Work, Environment and Health | 2020 | 46(6) | 11 |
| Scandinavian Journal of Work, Environment and Health | 2021 | 47(1) | 12 |
| Scandinavian Journal of Work, Environment and Health | 2021 | 47(2) | 9 |
| Scandinavian Journal of Work, Environment and Health | 2021 | 47(3) | 10 |
| Scandinavian Journal of Work, Environment and Health | 2021 | 47(4) | 9 |
| Scandinavian Journal of Work, Environment and Health | 2021 | 47(5) | 10 |
| Scandinavian Journal of Work, Environment and Health | 2021 | 47(6) | 8 |
| Scandinavian Journal of Work, Environment and Health | 2021 | 47(7) | 7 |
| Scandinavian Journal of Work, Environment and Health | 2021 | 47(8) | 7 |
| Scandinavian Journal of Work, Environment and Health | 2022 | 48(1) | 9 |
| Scandinavian Journal of Work, Environment and Health | 2022 | 48(2) | 9 |
| Scandinavian Journal of Work, Environment and Health | 2022 | 48(3) | 8 |
| Scandinavian Journal of Work, Environment and Health | 2022 | 48(4) | 8 |
| Scandinavian Journal of Work, Environment and Health | 2022 | 48(5) | 9 |
| Scandinavian Journal of Work, Environment and Health | 2022 | 48(6) | 9 |
| Scandinavian Journal of Work, Environment and Health | 2022 | 48(7) | 11 |
| Scandinavian Journal of Work, Environment and Health | 2022 | 48(8) | 9 |
| Policy and Practice in Health and Safety | 2015 | 13(1) | 7 |
| Policy and Practice in Health and Safety | 2015 | 13(2) | 6 |
| Policy and Practice in Health and Safety | 2016 | 14(1) | 7 |
| Policy and Practice in Health and Safety | 2016 | 14(2) | 5 |
| Policy and Practice in Health and Safety | 2017 | 15(1) | 8 |
| Policy and Practice in Health and Safety | 2017 | 15(2) | 11 |
| Policy and Practice in Health and Safety | 2018 | 16(1) | 9 |
| Policy and Practice in Health and Safety | 2018 | 16(2) | 6 |
| Policy and Practice in Health and Safety | 2019 | 17(1) | 7 |
| Policy and Practice in Health and Safety | 2019 | 17(2) | 7 |
| Policy and Practice in Health and Safety | 2020 | 18(1) | 6 |
| Policy and Practice in Health and Safety | 2020 | 18(2) | 11 |
| Journal of Labor Economics | 2015 | 33(1) | 7 |
| Journal of Labor Economics | 2015 | 33(2) | 7 |
| Journal of Labor Economics | 2015 | 33(3) Part 1 | 7 |
| Journal of Labor Economics | 2015 | 33(S1) Part 2 | 10 |
| Journal of Labor Economics | 2015 | 33(4) | 7 |
| Journal of Labor Economics | 2016 | 34(1) Part 1 | 7 |
| Journal of Labor Economics | 2016 | 34(S1) Part 2 | 12 |
| Journal of Labor Economics | 2016 | 34(2)b Part 1 | 7 |
| Journal of Labor Economics | 2016 | 34(S2) Part 2 | 9 |
| Journal of Labor Economics | 2016 | 34(3) | 7 |
| Journal of Labor Economics | 2016 | 34(4) | 7 |
| Journal of Labor Economics | 2017 | 35(1) | 7 |
| Journal of Labor Economics | 2017 | 35(2) | 7 |
| Journal of Labor Economics | 2017 | 35(3) | 7 |
| Journal of Labor Economics | 2017 | 35(S1) | 10 |
| Journal of Labor Economics | 2017 | 35(4) | 7 |
| Journal of Labor Economics | 2018 | 36(1) | 7 |
| Journal of Labor Economics | 2018 | 36(S1) | 9 |
| Journal of Labor Economics | 2018 | 36(2) | 7 |
| Journal of Labor Economics | 2018 | 36(3) | 7 |
| Journal of Labor Economics | 2018 | 36(4) | 7 |
| Journal of Labor Economics | 2019 | 37(S1) | 6 |
| Journal of Labor Economics | 2019 | 37(1) | 7 |
| Journal of Labor Economics | 2019 | 37(2) | 8 |
| Journal of Labor Economics | 2019 | 37(S2) | 13 |
| Journal of Labor Economics | 2019 | 37(3) | 8 |
| Journal of Labor Economics | 2019 | 37(4) | 8 |
| Journal of Labor Economics | 2020 | 38(1) | 8 |
| Journal of Labor Economics | 2020 | 38(2) | 8 |
| Journal of Labor Economics | 2020 | 38(3) | 6 |
| Journal of Labor Economics | 2020 | 38(4) | 10 |
| Journal of Labor Economics | 2021 | 39(S1) | 8 |
| Journal of Labor Economics | 2021 | 39(1) | 8 |
| Journal of Labor Economics | 2021 | 39(S2) | 10 |
| Journal of Labor Economics | 2021 | 39(2) | 8 |
| Journal of Labor Economics | 2021 | 39(3) | 6 |
| Journal of Labor Economics | 2021 | 39(4) | 10 |
| Journal of Labor Economics | 2022 | 40(1) | 7 |
| Journal of Labor Economics | 2022 | 40(2) | 7 |
| Journal of Labor Economics | 2022 | 40(S1) | 13 |
| Journal of Labor Economics | 2022 | 40(3) | 6 |
| Journal of Labor Economics | 2022 | 40(4) | 8 |
| Journal of Occupational and Environmental Medicine | 2015 | 57(1) | 23 |
| Journal of Occupational and Environmental Medicine | 2015 | 57(2) | 22 |
| Journal of Occupational and Environmental Medicine | 2015 | 57(3) | 23 |
| Journal of Occupational and Environmental Medicine | 2015 | 57(4) | 23 |
| Journal of Occupational and Environmental Medicine | 2015 | 57(5) | 20 |
| Journal of Occupational and Environmental Medicine | 2015 | 57(6) | 23 |
| Journal of Occupational and Environmental Medicine | 2015 | 57(7) | 26 |
| Journal of Occupational and Environmental Medicine | 2015 | 57(8) | 16 |
| Journal of Occupational and Environmental Medicine | 2015 | 57(9) | 16 |
| Journal of Occupational and Environmental Medicine | 2015 | 57(10) | 21 |
| Journal of Occupational and Environmental Medicine | 2015 | 57(11) | 22 |
| Journal of Occupational and Environmental Medicine | 2015 | 57(12) | 26 |
| Journal of Occupational and Environmental Medicine | 2016 | 58(1) | 27 |
| Journal of Occupational and Environmental Medicine | 2016 | 58(2) | 26 |
| Journal of Occupational and Environmental Medicine | 2016 | 58(3) | 28 |
| Journal of Occupational and Environmental Medicine | 2016 | 58(4) | 26 |
| Journal of Occupational and Environmental Medicine | 2016 | 58(5) | 26 |
| Journal of Occupational and Environmental Medicine | 2016 | 58(6) | 26 |
| Journal of Occupational and Environmental Medicine | 2016 | 58(7) | 23 |
| Journal of Occupational and Environmental Medicine | 2016 | 58(8) | 30 |
| Journal of Occupational and Environmental Medicine | 2016 | 58(9) | 25 |
| Journal of Occupational and Environmental Medicine | 2016 | 58(10) | 16 |
| Journal of Occupational and Environmental Medicine | 2016 | 58(11) | 23 |
| Journal of Occupational and Environmental Medicine | 2016 | 58(12) | 24 |
| Journal of Occupational and Environmental Medicine | 2017 | 59(1) | 21 |
| Journal of Occupational and Environmental Medicine | 2017 | 59(2) | 17 |
| Journal of Occupational and Environmental Medicine | 2017 | 59(3) | 18 |
| Journal of Occupational and Environmental Medicine | 2017 | 59(4) | 26 |
| Journal of Occupational and Environmental Medicine | 2017 | 59(5) | 20 |
| Journal of Occupational and Environmental Medicine | 2017 | 59(6) | 25 |
| Journal of Occupational and Environmental Medicine | 2017 | 59(7) | 18 |
| Journal of Occupational and Environmental Medicine | 2017 | 59(8) | 21 |
| Journal of Occupational and Environmental Medicine | 2017 | 59(9) | 14 |
| Journal of Occupational and Environmental Medicine | 2017 | 59(10) | 25 |
| Journal of Occupational and Environmental Medicine | 2017 | 59(11) | 27 |
| Journal of Occupational and Environmental Medicine | 2017 | 59(12) | 19 |
| Journal of Occupational and Environmental Medicine | 2018 | 60(1) | 24 |
| Journal of Occupational and Environmental Medicine | 2018 | 60(2) | 21 |
| Journal of Occupational and Environmental Medicine | 2018 | 60(3) | 18 |
| Journal of Occupational and Environmental Medicine | 2018 | 60(4) | 23 |
| Journal of Occupational and Environmental Medicine | 2018 | 60(5) | 23 |
| Journal of Occupational and Environmental Medicine | 2018 | 60(6) | 18 |
| Journal of Occupational and Environmental Medicine | 2018 | 60(7) | 23 |
| Journal of Occupational and Environmental Medicine | 2018 | 60(8) | 31 |
| Journal of Occupational and Environmental Medicine | 2018 | 60(9) | 28 |
| Journal of Occupational and Environmental Medicine | 2018 | 60(10) | 25 |
| Journal of Occupational and Environmental Medicine | 2018 | 60(11) | 24 |
| Journal of Occupational and Environmental Medicine | 2018 | 60(12) | 24 |
| Journal of Occupational and Environmental Medicine | 2019 | 61(1) | 16 |
| Journal of Occupational and Environmental Medicine | 2019 | 61(2) | 27 |
| Journal of Occupational and Environmental Medicine | 2019 | 61(3) | 15 |
| Journal of Occupational and Environmental Medicine | 2019 | 61(4) | 23 |
| Journal of Occupational and Environmental Medicine | 2019 | 61(5) | 24 |
| Journal of Occupational and Environmental Medicine | 2019 | 61(6) | 26 |
| Journal of Occupational and Environmental Medicine | 2019 | 61(7) | 15 |
| Journal of Occupational and Environmental Medicine | 2019 | 61(8) | 18 |
| Journal of Occupational and Environmental Medicine | 2019 | 61(9) | 20 |
| Journal of Occupational and Environmental Medicine | 2019 | 61(10) | 18 |
| Journal of Occupational and Environmental Medicine | 2019 | 61(11) | 19 |
| Journal of Occupational and Environmental Medicine | 2019 | 61(12) | 32 |
| Journal of Occupational and Environmental Medicine | 2020 | 62(1) | 21 |
| Journal of Occupational and Environmental Medicine | 2020 | 62(2) | 24 |
| Journal of Occupational and Environmental Medicine | 2020 | 62(3) | 17 |
| Journal of Occupational and Environmental Medicine | 2020 | 62(4) | 17 |
| Journal of Occupational and Environmental Medicine | 2020 | 62(5) | 18 |
| Journal of Occupational and Environmental Medicine | 2020 | 62(6) | 19 |
| Journal of Occupational and Environmental Medicine | 2020 | 62(7) | 26 |
| Journal of Occupational and Environmental Medicine | 2020 | 62(8) | 32 |
| Journal of Occupational and Environmental Medicine | 2020 | 62(9) | 29 |
| Journal of Occupational and Environmental Medicine | 2020 | 62(10) | 27 |
| Journal of Occupational and Environmental Medicine | 2020 | 62(11) | 28 |
| Journal of Occupational and Environmental Medicine | 2020 | 62(12) | 35 |
| Journal of Occupational and Environmental Medicine | 2021 | 63(1) | 20 |
| Journal of Occupational and Environmental Medicine | 2021 | 63(2) | 25 |
| Journal of Occupational and Environmental Medicine | 2021 | 63(3) | 25 |
| Journal of Occupational and Environmental Medicine | 2021 | 63(4) | 25 |
| Journal of Occupational and Environmental Medicine | 2021 | 63(5) | 22 |
| Journal of Occupational and Environmental Medicine | 2021 | 63(6) | 29 |
| Journal of Occupational and Environmental Medicine | 2021 | 63(7) | 27 |
| Journal of Occupational and Environmental Medicine | 2021 | 63(8) | 25 |
| Journal of Occupational and Environmental Medicine | 2021 | 63(9) | 26 |
| Journal of Occupational and Environmental Medicine | 2021 | 63(10) | 29 |
| Journal of Occupational and Environmental Medicine | 2021 | 63(11) | 33 |
| Journal of Occupational and Environmental Medicine | 2021 | 63(12) | 32 |
| Journal of Occupational and Environmental Medicine | 2022 | 64(1) | 22 |
| Journal of Occupational and Environmental Medicine | 2022 | 64(2) | 22 |
| Journal of Occupational and Environmental Medicine | 2022 | 64(3) | 26 |
| Journal of Occupational and Environmental Medicine | 2022 | 64(4) | 26 |
| Journal of Occupational and Environmental Medicine | 2022 | 64(5) | 28 |
| Journal of Occupational and Environmental Medicine | 2022 | 64(6) | 23 |
| Journal of Occupational and Environmental Medicine | 2022 | 64(7) | 20 |
| Journal of Occupational and Environmental Medicine | 2022 | 64(8) | 27 |
| Journal of Occupational and Environmental Medicine | 2022 | 64(9) | 25 |
| Journal of Occupational and Environmental Medicine | 2022 | 64(10) | 24 |
| Journal of Occupational and Environmental Medicine | 2022 | 64(11) | 35 |
| Journal of Occupational and Environmental Medicine | 2022 | 64(12) | 23 |
| Journal of Safety Research | 2015 | 52 | 9 |
| Journal of Safety Research | 2015 | 53 | 13 |
| Journal of Safety Research | 2015 | 54 | 18 |
| Journal of Safety Research | 2015 | 55 | 23 |
| Journal of Safety Research | 2016 | 56 | 15 |
| Journal of Safety Research | 2016 | 57 | 11 |
| Journal of Safety Research | 2016 | 58 | 12 |
| Journal of Safety Research | 2016 | 59 | 13 |
| Journal of Safety Research | 2017 | 60 | 18 |
| Journal of Safety Research | 2017 | 61 | 22 |
| Journal of Safety Research | 2017 | 62 | 27 |
| Journal of Safety Research | 2017 | 63 | 27 |
| Journal of Safety Research | 2018 | 64 | 18 |
| Journal of Safety Research | 2018 | 65 | 18 |
| Journal of Safety Research | 2018 | 66 | 22 |
| Journal of Safety Research | 2018 | 67 | 26 |
| Journal of Safety Research | 2019 | 68 | 25 |
| Journal of Safety Research | 2019 | 69 | 22 |
| Journal of Safety Research | 2019 | 70 | 33 |
| Journal of Safety Research | 2019 | 71 | 31 |
| Journal of Safety Research | 2020 | 72 | 32 |
| Journal of Safety Research | 2020 | 73 | 34 |
| Journal of Safety Research | 2020 | 74 | 34 |
| Journal of Safety Research | 2020 | 75 | 34 |
| Journal of Safety Research | 2021 | 76 | 36 |
| Journal of Safety Research | 2021 | 77 | 35 |
| Journal of Safety Research | 2021 | 78 | 33 |
| Journal of Safety Research | 2021 | 79 | 34 |
| Journal of Safety Research | 2022 | 80 | 43 |
| Journal of Safety Research | 2022 | 81 | 33 |
| Journal of Safety Research | 2022 | 82 | 49 |
| Journal of Safety Research | 2022 | 83 | 43 |
| Regulation and Governance | 2015 | 9 (1) | 6 |
| Regulation and Governance | 2015 | 9 (2) | 7 |
| Regulation and Governance | 2015 | 9 (3) | 8 |
| Regulation and Governance | 2015 | 9 (4) | 5 |
| Regulation and Governance | 2016 | 10 (1) | 7 |
| Regulation and Governance | 2016 | 10 (2) | 6 |
| Regulation and Governance | 2016 | 10 (3) | 6 |
| Regulation and Governance | 2016 | 10 (4) | 6 |
| Regulation and Governance | 2017 | 11 (1) | 7 |
| Regulation and Governance | 2017 | 11 (2) | 7 |
| Regulation and Governance | 2017 | 11 (3) | 6 |
| Regulation and Governance | 2017 | 11 (4) | 8 |
| Regulation and Governance | 2018 | 12 (1) | 9 |
| Regulation and Governance | 2018 | 12 (2) | 7 |
| Regulation and Governance | 2018 | 12 (3) | 6 |
| Regulation and Governance | 2018 | 12 (4) | 6 |
| Regulation and Governance | 2019 | 13 (1) | 7 |
| Regulation and Governance | 2019 | 13 (2) | 9 |
| Regulation and Governance | 2019 | 13 (3) | 7 |
| Regulation and Governance | 2019 | 13 (4) | 9 |
| Regulation and Governance | 2020 | 14 (1) | 8 |
| Regulation and Governance | 2020 | 14 (2) | 12 |
| Regulation and Governance | 2020 | 14 (3) | 12 |
| Regulation and Governance | 2020 | 14 (4) | 12 |
| Regulation and Governance | 2021 | 15 (1) | 13 |
| Regulation and Governance | 2021 | 15 (2) | 11 |
| Regulation and Governance | 2021 | 15 (3) | 27 |
| Regulation and Governance | 2021 | 15 (4) | 25 |
| Regulation and Governance | 2021 | 15 (s1) | 9 |
| Regulation and Governance | 2022 | 16 (1) | 19 |
| Regulation and Governance | 2022 | 16 (2) | 15 |
| Regulation and Governance | 2022 | 16(3) | 19 |
| Regulation and Governance | 2022 | 16(4) | 29 |
| Law & Policy | 2015 | 37 (1-2) | 5 |
| Law & Policy | 2015 | 37 (3) | 4 |
| Law & Policy | 2015 | 37 (4) | 4 |
| Law & Policy | 2016 | 38 (1) | 4 |
| Law & Policy | 2016 | 38 (2) | 4 |
| Law & Policy | 2016 | 38 (3) | 5 |
| Law & Policy | 2016 | 38 (4) | 4 |
| Law & Policy | 2017 | 39 (1) | 4 |
| Law & Policy | 2017 | 39 (2) | 5 |
| Law & Policy | 2017 | 39 (3) | 4 |
| Law & Policy | 2017 | 39 (4) | 6 |
| Law & Policy | 2018 | 40 (1) | 6 |
| Law & Policy | 2018 | 40 (2) | 4 |
| Law & Policy | 2018 | 40 (3) | 4 |
| Law & Policy | 2018 | 40 (4) | 4 |
| Law & Policy | 2019 | 41 (1) | 7 |
| Law & Policy | 2019 | 41 (2) | 5 |
| Law & Policy | 2019 | 41 (3) | 4 |
| Law & Policy | 2019 | 41 (4) | 4 |
| Law & Policy | 2020 | 42 (1) | 4 |
| Law & Policy | 2020 | 42 (2) | 4 |
| Law & Policy | 2020 | 42 (3) | 4 |
| Law & Policy | 2020 | 42 (4) | 4 |
| Law & Policy | 2021 | 43 (1) | 4 |
| Law & Policy | 2021 | 43 (2) | 4 |
| Law & Policy | 2021 | 43 (3) | 4 |
| Law & Policy | 2021 | 43 (4) | 4 |
| Law & Policy | 2022 | 44 (1) | 5 |
| Law & Policy | 2022 | 44 (2) | 4 |
| Law & Policy | 2022 | 44(3) | 4 |
| Law & Policy | 2022 | 44(4) | 5 |
| Journal of Industrial Relations | 2015 | 57 (1) | 8 |
| Journal of Industrial Relations | 2015 | 57 (2) | 9 |
| Journal of Industrial Relations | 2015 | 57 (3) | 8 |
| Journal of Industrial Relations | 2015 | 57 (4) | 9 |
| Journal of Industrial Relations | 2015 | 57 (5) | 8 |
| Journal of Industrial Relations | 2016 | 58 (1) | 11 |
| Journal of Industrial Relations | 2016 | 58 (2) | 10 |
| Journal of Industrial Relations | 2016 | 58 (3) | 12 |
| Journal of Industrial Relations | 2016 | 58 (4) | 6 |
| Journal of Industrial Relations | 2016 | 58 (5) | 6 |
| Journal of Industrial Relations | 2017 | 59 (1) | 8 |
| Journal of Industrial Relations | 2017 | 59 (2) | 6 |
| Journal of Industrial Relations | 2017 | 59 (3) | 9 |
| Journal of Industrial Relations | 2017 | 59 (4) | 7 |
| Journal of Industrial Relations | 2017 | 59 (5) | 4 |
| Journal of Industrial Relations | 2018 | 60 (1) | 5 |
| Journal of Industrial Relations | 2018 | 60 (2) | 4 |
| Journal of Industrial Relations | 2018 | 60 (3) | 9 |
| Journal of Industrial Relations | 2018 | 60 (4) | 5 |
| Journal of Industrial Relations | 2018 | 60 (5) | 7 |
| Journal of Industrial Relations | 2019 | 61 (1) | 5 |
| Journal of Industrial Relations | 2019 | 61 (2) | 6 |
| Journal of Industrial Relations | 2019 | 61 (3) | 7 |
| Journal of Industrial Relations | 2019 | 61 (4) | 5 |
| Journal of Industrial Relations | 2019 | 61 (5) | 9 |
| Journal of Industrial Relations | 2020 | 62 (1) | 6 |
| Journal of Industrial Relations | 2020 | 62 (2) | 9 |
| Journal of Industrial Relations | 2020 | 62 (3) | 9 |
| Journal of Industrial Relations | 2020 | 62 (4) | 7 |
| Journal of Industrial Relations | 2020 | 62 (5) | 4 |
| Journal of Industrial Relations | 2021 | 63 (1) | 4 |
| Journal of Industrial Relations | 2021 | 63 (2) | 7 |
| Journal of Industrial Relations | 2021 | 63 (3) | 10 |
| Journal of Industrial Relations | 2021 | 63 (4) | 7 |
| Journal of Industrial Relations | 2021 | 63 (5) | 6 |
| Journal of Industrial Relations | 2022 | 64 (1) | 6 |
| Journal of Industrial Relations | 2022 | 64 (2) | 6 |
| Journal of Industrial Relations | 2022 | 64 (3) | 8 |
| Journal of Industrial Relations | 2022 | 64(4) | 5 |
| Journal of Industrial Relations | 2022 | 64(5) | 8 |
| International Journal of Environmental Health Research | 2015 | 25 (1) | 9 |
| International Journal of Environmental Health Research | 2015 | 25 (2) | 9 |
| International Journal of Environmental Health Research | 2015 | 25 (3) | 10 |
| International Journal of Environmental Health Research | 2015 | 25 (4) | 9 |
| International Journal of Environmental Health Research | 2015 | 25 (5) | 8 |
| International Journal of Environmental Health Research | 2015 | 25 (6) | 8 |
| International Journal of Environmental Health Research | 2016 | 26 (1) | 7 |
| International Journal of Environmental Health Research | 2016 | 26 (2) | 8 |
| International Journal of Environmental Health Research | 2016 | 26 (3) | 9 |
| International Journal of Environmental Health Research | 2016 | 26 (4) | 8 |
| International Journal of Environmental Health Research | 2016 | 26 (5-6) | 12 |
| International Journal of Environmental Health Research | 2017 | 27 (1) | 7 |
| International Journal of Environmental Health Research | 2017 | 27 (2) | 6 |
| International Journal of Environmental Health Research | 2017 | 27 (3) | 7 |
| International Journal of Environmental Health Research | 2017 | 27 (4) | 6 |
| International Journal of Environmental Health Research | 2017 | 27 (5) | 10 |
| International Journal of Environmental Health Research | 2017 | 27 (6) | 6 |
| International Journal of Environmental Health Research | 2018 | 28 (1) | 10 |
| International Journal of Environmental Health Research | 2018 | 28 (2) | 8 |
| International Journal of Environmental Health Research | 2018 | 28 (3) | 8 |
| International Journal of Environmental Health Research | 2018 | 28 (4) | 8 |
| International Journal of Environmental Health Research | 2018 | 28 (5) | 8 |
| International Journal of Environmental Health Research | 2018 | 28 (6) | 10 |
| International Journal of Environmental Health Research | 2019 | 29 (1) | 9 |
| International Journal of Environmental Health Research | 2019 | 29 (2) | 10 |
| International Journal of Environmental Health Research | 2019 | 29 (3) | 10 |
| International Journal of Environmental Health Research | 2019 | 29 (4) | 8 |
| International Journal of Environmental Health Research | 2019 | 29 (5) | 8 |
| International Journal of Environmental Health Research | 2019 | 29 (6) | 9 |
| International Journal of Environmental Health Research | 2020 | 30 (1) | 9 |
| International Journal of Environmental Health Research | 2020 | 30 (2) | 9 |
| International Journal of Environmental Health Research | 2020 | 30 (3) | 9 |
| International Journal of Environmental Health Research | 2020 | 30 (4) | 8 |
| International Journal of Environmental Health Research | 2020 | 30 (5) | 8 |
| International Journal of Environmental Health Research | 2020 | 30 (6) | 8 |
| International Journal of Environmental Health Research | 2021 | 31 (1) | 9 |
| International Journal of Environmental Health Research | 2021 | 31 (2) | 9 |
| International Journal of Environmental Health Research | 2021 | 31 (3) | 9 |
| International Journal of Environmental Health Research | 2021 | 31 (4) | 8 |
| International Journal of Environmental Health Research | 2021 | 31 (5) | 9 |
| International Journal of Environmental Health Research | 2021 | 31 (6) | 10 |
| International Journal of Environmental Health Research | 2021 | 31 (7) | 10 |
| International Journal of Environmental Health Research | 2021 | 31 (8) | 10 |
| International Journal of Environmental Health Research | 2022 | 32 (1) | 18 |
| International Journal of Environmental Health Research | 2022 | 32 (2) | 17 |
| International Journal of Environmental Health Research | 2022 | 32 (3) | 16 |
| International Journal of Environmental Health Research | 2022 | 32 (4) | 17 |
| International Journal of Environmental Health Research | 2022 | 32 (5) | 21 |
| International Journal of Environmental Health Research | 2022 | 32 (6) | 21 |
| International Journal of Environmental Health Research | 2022 | 32 (7) | 16 |
| International Journal of Environmental Health Research | 2022 | 32 (8) | 15 |
| International Journal of Environmental Health Research | 2022 | 32(9) | 19 |
| International Journal of Environmental Health Research | 2022 | 32(10) | 16 |
| International Journal of Environmental Health Research | 2022 | 32(11) | 18 |
| International Journal of Environmental Health Research | 2022 | 32(12) | 15 |
| Accident Analysis & Prevention | 2015 | 74 | 39 |
| Accident Analysis & Prevention | 2015 | 75 | 34 |
| Accident Analysis & Prevention | 2015 | 76 | 19 |
| Accident Analysis & Prevention | 2015 | 77 | 16 |
| Accident Analysis & Prevention | 2015 | 78 | 24 |
| Accident Analysis & Prevention | 2015 | 79 | 23 |
| Accident Analysis & Prevention | 2015 | 80 | 30 |
| Accident Analysis & Prevention | 2015 | 81 | 25 |
| Accident Analysis & Prevention | 2015 | 82 | 30 |
| Accident Analysis & Prevention | 2015 | 83 | 23 |
| Accident Analysis & Prevention | 2015 | 84 | 18 |
| Accident Analysis & Prevention | 2015 | 85 | 24 |
| Accident Analysis & Prevention | 2016 | 86 | 27 |
| Accident Analysis & Prevention | 2016 | 87 | 18 |
| Accident Analysis & Prevention | 2016 | 88 | 20 |
| Accident Analysis & Prevention | 2016 | 89 | 16 |
| Accident Analysis & Prevention | 2016 | 90 | 15 |
| Accident Analysis & Prevention | 2016 | 91 | 24 |
| Accident Analysis & Prevention | 2016 | 92 | 28 |
| Accident Analysis & Prevention | 2016 | 93 | 33 |
| Accident Analysis & Prevention | 2016 | 94 | 27 |
| Accident Analysis & Prevention | 2016 | 95 (Part A) | 33 |
| Accident Analysis & Prevention | 2016 | 95 (Part B) | 25 |
| Accident Analysis & Prevention | 2016 | 96 | 39 |
| Accident Analysis & Prevention | 2016 | 97 | 34 |
| Accident Analysis & Prevention | 2017 | 98 | 41 |
| Accident Analysis & Prevention | 2017 | 99 (Part A) | 40 |
| Accident Analysis & Prevention | 2017 | 99 (Part B) | 17 |
| Accident Analysis & Prevention | 2017 | 100 | 15 |
| Accident Analysis & Prevention | 2017 | 101 | 17 |
| Accident Analysis & Prevention | 2017 | 102 | 25 |
| Accident Analysis & Prevention | 2017 | 103 | 18 |
| Accident Analysis & Prevention | 2017 | 104 | 20 |
| Accident Analysis & Prevention | 2017 | 105 | 17 |
| Accident Analysis & Prevention | 2017 | 106 | 55 |
| Accident Analysis & Prevention | 2017 | 107 | 20 |
| Accident Analysis & Prevention | 2017 | 108 | 39 |
| Accident Analysis & Prevention | 2017 | 109 | 15 |
| Accident Analysis & Prevention | 2018 | 110 | 20 |
| Accident Analysis & Prevention | 2018 | 111 | 37 |
| Accident Analysis & Prevention | 2018 | 112 | 15 |
| Accident Analysis & Prevention | 2018 | 113 | 36 |
| Accident Analysis & Prevention | 2018 | 114 | 12 |
| Accident Analysis & Prevention | 2018 | 115 | 22 |
| Accident Analysis & Prevention | 2018 | 116 | 12 |
| Accident Analysis & Prevention | 2018 | 117 | 46 |
| Accident Analysis & Prevention | 2018 | 118 | 31 |
| Accident Analysis & Prevention | 2018 | 119 | 29 |
| Accident Analysis & Prevention | 2018 | 120 | 30 |
| Accident Analysis & Prevention | 2018 | 121 | 38 |
| Accident Analysis & Prevention | 2019 | 122 | 38 |
| Accident Analysis & Prevention | 2019 | 123 | 48 |
| Accident Analysis & Prevention | 2019 | 124 | 24 |
| Accident Analysis & Prevention | 2019 | 125 | 40 |
| Accident Analysis & Prevention | 2019 | 126 | 25 |
| Accident Analysis & Prevention | 2019 | 127 | 25 |
| Accident Analysis & Prevention | 2019 | 128 | 28 |
| Accident Analysis & Prevention | 2019 | 129 | 36 |
| Accident Analysis & Prevention | 2019 | 130 | 17 |
| Accident Analysis & Prevention | 2019 | 131 | 34 |
| Accident Analysis & Prevention | 2019 | 132 | 39 |
| Accident Analysis & Prevention | 2019 | 133 | 15 |
| Accident Analysis & Prevention | 2020 | 134 | 24 |
| Accident Analysis & Prevention | 2020 | 135 | 33 |
| Accident Analysis & Prevention | 2020 | 136 | 27 |
| Accident Analysis & Prevention | 2020 | 137 | 19 |
| Accident Analysis & Prevention | 2020 | 138 | 17 |
| Accident Analysis & Prevention | 2020 | 139 | 13 |
| Accident Analysis & Prevention | 2020 | 140 | 4 |
| Accident Analysis & Prevention | 2020 | 141 | 18 |
| Accident Analysis & Prevention | 2020 | 142 | 31 |
| Accident Analysis & Prevention | 2020 | 143 | 12 |
| Accident Analysis & Prevention | 2020 | 144 | 73 |
| Accident Analysis & Prevention | 2020 | 145 | 23 |
| Accident Analysis & Prevention | 2020 | 146 | 38 |
| Accident Analysis & Prevention | 2020 | 147 | 13 |
| Accident Analysis & Prevention | 2020 | 148 | 41 |
| Accident Analysis & Prevention | 2021 | 149 | 26 |
| Accident Analysis & Prevention | 2021 | 150 | 46 |
| Accident Analysis & Prevention | 2021 | 151 | 47 |
| Accident Analysis & Prevention | 2021 | 152 | 23 |
| Accident Analysis & Prevention | 2021 | 153 | 24 |
| Accident Analysis & Prevention | 2021 | 154 | 31 |
| Accident Analysis & Prevention | 2021 | 155 | 10 |
| Accident Analysis & Prevention | 2021 | 156 | 25 |
| Accident Analysis & Prevention | 2021 | 157 | 35 |
| Accident Analysis & Prevention | 2021 | 158 | 12 |
| Accident Analysis & Prevention | 2021 | 159 | 73 |
| Accident Analysis & Prevention | 2021 | 160 | 28 |
| Accident Analysis & Prevention | 2021 | 161 | 33 |
| Accident Analysis & Prevention | 2021 | 162 | 24 |
| Accident Analysis & Prevention | 2021 | 163 | 32 |
| Accident Analysis & Prevention | 2022 | 164 | 11 |
| Accident Analysis & Prevention | 2022 | 165 | 28 |
| Accident Analysis & Prevention | 2022 | 166 | 17 |
| Accident Analysis & Prevention | 2022 | 167 | 12 |
| Accident Analysis & Prevention | 2022 | 168 | 22 |
| Accident Analysis & Prevention | 2022 | 169 | 9 |
| Accident Analysis & Prevention | 2022 | 170 | 9 |
| Accident Analysis & Prevention | 2022 | 171 | 14 |
| Accident Analysis & Prevention | 2022 | 172 | 9 |
| Accident Analysis & Prevention | 2022 | 173 | 19 |
| Accident Analysis & Prevention | 2022 | 174 | 31 |
| Accident Analysis & Prevention | 2022 | 175 | 8 |
| Accident Analysis & Prevention | 2022 | 176 | 16 |
| Accident Analysis & Prevention | 2022 | 177 | 15 |
| Accident Analysis & Prevention | 2022 | 178 | 15 |
| Safety and Health at Work | 2015 | 6 (Issue 1) | 10 |
| Safety and Health at Work | 2015 | 6 (issue 2) | 9 |
| Safety and Health at Work | 2015 | 6 (issue 3) | 13 |
| Safety and Health at Work | 2015 | 6 (issue 4) | 12 |
| Safety and Health at Work | 2016 | 7 (issue 1) | 12 |
| Safety and Health at Work | 2016 | 7 (Issue 2) | 11 |
| Safety and Health at Work | 2016 | 7 Issue 3) | 12 |
| Safety and Health at Work | 2016 | 7 (Issue 4) | 16 |
| Safety and Health at Work | 2017 | 8 (Issue 1) | 16 |
| Safety and Health at Work | 2017 | 8 (Issue 2) | 14 |
| Safety and Health at Work | 2017 | 8 (Issue 3) | 11 |
| Safety and Health at Work | 2017 | 8 (Issue 4) | 12 |
| Safety and Health at Work | 2018 | 9 (Issue 1) | 17 |
| Safety and Health at Work | 2018 | 9 (Issue 2) | 16 |
| Safety and Health at Work | 2018 | 9 (Issue 3) | 14 |
| Safety and Health at Work | 2018 | 9 (Issue 4) | 16 |
| Safety and Health at Work | 2019 | 10 (Issue 1) | 16 |
| Safety and Health at Work | 2019 | 10(Issue 2) | 14 |
| Safety and Health at Work | 2019 | 10 (Issue 3) | 15 |
| Safety and Health at Work | 2019 | 10 (Issue 4) | 15 |
| Safety and Health at Work | 2020 | 11 (Issue 1) | 15 |
| Safety and Health at Work | 2020 | 11 (Issue 2) | 16 |
| Safety and Health at Work | 2020 | 11(Issue 3) | 19 |
| Safety and Health at Work | 2020 | 11 (Issue 4) | 20 |
| Safety and Health at Work | 2021 | 12 (Issue 1) | 16 |
| Safety and Health at Work | 2021 | 12 (Issue2) | 17 |
| Safety and Health at Work | 2021 | 12 (Issue 3) | 17 |
| Safety and Health at Work | 2021 | 12 (Issue 4) | 16 |
| Safety and Health at Work | 2022 | 13(Issue 1) | 17 |
| Safety and Health at Work | 2022 | 13 (Issue 2) | 18 |
| Safety and Health at Work | 2022 | 13 (Issue 3) | 15 |
| Safety and Health at Work | 2022 | 13 (Issue 4) | 19 |
| Industrial Law Journal | 2015 | 44(1) | 10 |
| Industrial Law Journal | 2015 | 44(2) | 10 |
| Industrial Law Journal | 2015 | 44(3) | 15 |
| Industrial Law Journal | 2015 | 44(4) | 6 |
| Industrial Law Journal | 2016 | 45(1) | 5 |
| Industrial Law Journal | 2016 | 45(2) | 14 |
| Industrial Law Journal | 2016 | 45(3) | 10 |
| Industrial Law Journal | 2016 | 45(4) | 5 |
| Industrial Law Journal | 2017 | 46(1) | 10 |
| Industrial Law Journal | 2017 | 46(2) | 7 |
| Industrial Law Journal | 2017 | 46(3) | 7 |
| Industrial Law Journal | 2017 | 46(4) | 6 |
| Industrial Law Journal | 2018 | 47(1) | 9 |
| Industrial Law Journal | 2018 | 47(2) | 8 |
| Industrial Law Journal | 2018 | 47(3) | 7 |
| Industrial Law Journal | 2018 | 47(4) | 7 |
| Industrial Law Journal | 2019 | 48(1) | 6 |
| Industrial Law Journal | 2019 | 48(2) | 11 |
| Industrial Law Journal | 2019 | 48(3) | 6 |
| Industrial Law Journal | 2019 | 48(4) | 5 |
| Industrial Law Journal | 2020 | 49(1) | 5 |
| Industrial Law Journal | 2020 | 49(2) | 4 |
| Industrial Law Journal | 2020 | 49(3) | 9 |
| Industrial Law Journal | 2020 | 49(4) | 8 |
| Industrial Law Journal | 2021 | 50(1) | 7 |
| Industrial Law Journal | 2021 | 50(2) | 9 |
| Industrial Law Journal | 2021 | 50(3) | 6 |
| Industrial Law Journal | 2021 | 50(4) | 9 |
| Industrial Law Journal | 2022 | 51(1) | 13 |
| Industrial Law Journal | 2022 | 51(2) | 12 |
| Industrial Law Journal | 2022 | 51(3) | 11 |
| American Journal of Industrial Medicine | 2015 | 58(1) | 12 |
| American Journal of Industrial Medicine | 2015 | 58(2) | 12 |
| American Journal of Industrial Medicine | 2015 | 58(3) | 10 |
| American Journal of Industrial Medicine | 2015 | 58(4) | 11 |
| American Journal of Industrial Medicine | 2015 | 58(5) | 11 |
| American Journal of Industrial Medicine | 2015 | 58(6) | 12 |
| American Journal of Industrial Medicine | 2015 | 58(7) | 12 |
| American Journal of Industrial Medicine | 2015 | 58(8) | 12 |
| American Journal of Industrial Medicine | 2015 | 58(9) | 10 |
| American Journal of Industrial Medicine | 2015 | 58(10) | 14 |
| American Journal of Industrial Medicine | 2016 | 58(S1) | 10 |
| American Journal of Industrial Medicine | 2015 | 58(11) | 10 |
| American Journal of Industrial Medicine | 2015 | 58(12) | 11 |
| American Journal of Industrial Medicine | 2016 | 59(1) | 9 |
| American Journal of Industrial Medicine | 2016 | 59(2) | 8 |
| American Journal of Industrial Medicine | 2016 | 59(3) | 10 |
| American Journal of Industrial Medicine | 2016 | 59(4) | 13 |
| American Journal of Industrial Medicine | 2016 | 59(5) | 8 |
| American Journal of Industrial Medicine | 2016 | 59(6) | 9 |
| American Journal of Industrial Medicine | 2016 | 59(7) | 9 |
| American Journal of Industrial Medicine | 2016 | 59(8) | 10 |
| American Journal of Industrial Medicine | 2016 | 59(9) | 14 |
| American Journal of Industrial Medicine | 2016 | 59(10) | 8 |
| American Journal of Industrial Medicine | 2016 | 59(11) | 13 |
| American Journal of Industrial Medicine | 2016 | 59(12) | 14 |
| American Journal of Industrial Medicine | 2017 | 60(1) | 14 |
| American Journal of Industrial Medicine | 2017 | 60(2) | 10 |
| American Journal of Industrial Medicine | 2017 | 60(3) | 11 |
| American Journal of Industrial Medicine | 2017 | 60(4) | 9 |
| American Journal of Industrial Medicine | 2017 | 60(5) | 13 |
| American Journal of Industrial Medicine | 2017 | 60(6) | 10 |
| American Journal of Industrial Medicine | 2017 | 60(7) | 9 |
| American Journal of Industrial Medicine | 2017 | 60(8) | 8 |
| American Journal of Industrial Medicine | 2017 | 60(9) | 11 |
| American Journal of Industrial Medicine | 2017 | 60(10) | 9 |
| American Journal of Industrial Medicine | 2017 | 60(11) | 10 |
| American Journal of Industrial Medicine | 2017 | 60(12) | 11 |
| American Journal of Industrial Medicine | 2018 | 61(1) | 10 |
| American Journal of Industrial Medicine | 2018 | 61(2) | 10 |
| American Journal of Industrial Medicine | 2018 | 61(3) | 13 |
| American Journal of Industrial Medicine | 2018 | 61(4) | 12 |
| American Journal of Industrial Medicine | 2018 | 61(5) | 13 |
| American Journal of Industrial Medicine | 2018 | 61(6) | 12 |
| American Journal of Industrial Medicine | 2018 | 61(7) | 11 |
| American Journal of Industrial Medicine | 2018 | 61(8) | 10 |
| American Journal of Industrial Medicine | 2018 | 61(9) | 11 |
| American Journal of Industrial Medicine | 2018 | 61(10) | 10 |
| American Journal of Industrial Medicine | 2018 | 61(11) | 10 |
| American Journal of Industrial Medicine | 2018 | 61(12) | 7 |
| American Journal of Industrial Medicine | 2019 | 62(1) | 11 |
| American Journal of Industrial Medicine | 2019 | 62(2) | 10 |
| American Journal of Industrial Medicine | 2019 | 62(3) | 10 |
| American Journal of Industrial Medicine | 2019 | 62(4) | 11 |
| American Journal of Industrial Medicine | 2019 | 62(5) | 10 |
| American Journal of Industrial Medicine | 2019 | 62(6) | 11 |
| American Journal of Industrial Medicine | 2019 | 62(7) | 12 |
| American Journal of Industrial Medicine | 2019 | 62(8) | 10 |
| American Journal of Industrial Medicine | 2019 | 62(9) | 10 |
| American Journal of Industrial Medicine | 2019 | 62(10) | 10 |
| American Journal of Industrial Medicine | 2019 | 62(11) | 11 |
| American Journal of Industrial Medicine | 2019 | 62(12) | 15 |
| American Journal of Industrial Medicine | 2020 | 63(1) | 13 |
| American Journal of Industrial Medicine | 2020 | 63(2) | 10 |
| American Journal of Industrial Medicine | 2020 | 63(3) | 10 |
| American Journal of Industrial Medicine | 2020 | 63(4) | 9 |
| American Journal of Industrial Medicine | 2020 | 63(5) | 9 |
| American Journal of Industrial Medicine | 2020 | 63(6) | 10 |
| American Journal of Industrial Medicine | 2020 | 63(7) | 11 |
| American Journal of Industrial Medicine | 2020 | 63(8) | 11 |
| American Journal of Industrial Medicine | 2020 | 63(9) | 16 |
| American Journal of Industrial Medicine | 2020 | 63(10) | 13 |
| American Journal of Industrial Medicine | 2020 | 63(11) | 14 |
| American Journal of Industrial Medicine | 2020 | 63(12) | 12 |
| American Journal of Industrial Medicine | 2021 | 64(1) | 7 |
| American Journal of Industrial Medicine | 2021 | 64(2) | 10 |
| American Journal of Industrial Medicine | 2021 | 64(3) | 11 |
| American Journal of Industrial Medicine | 2021 | 64(4) | 12 |
| American Journal of Industrial Medicine | 2021 | 64(5) | 11 |
| American Journal of Industrial Medicine | 2021 | 64(6) | 9 |
| American Journal of Industrial Medicine | 2021 | 64(7) | 11 |
| American Journal of Industrial Medicine | 2021 | 64(8) | 8 |
| American Journal of Industrial Medicine | 2021 | 64(9) | 8 |
| American Journal of Industrial Medicine | 2021 | 64(10) | 12 |
| American Journal of Industrial Medicine | 2021 | 64(11) | 9 |
| American Journal of Industrial Medicine | 2021 | 64(12) | 10 |
| American Journal of Industrial Medicine | 2022 | 65(1) | 9 |
| American Journal of Industrial Medicine | 2022 | 65(2) | 8 |
| American Journal of Industrial Medicine | 2022 | 65(3) | 8 |
| American Journal of Industrial Medicine | 2022 | 65(4) | 8 |
| American Journal of Industrial Medicine | 2022 | 65(5) | 10 |
| American Journal of Industrial Medicine | 2022 | 65(6) | 8 |
| American Journal of Industrial Medicine | 2022 | 65(7) | 12 |
| American Journal of Industrial Medicine | 2022 | 65(8) | 9 |
| American Journal of Industrial Medicine | 2022 | 65(9) | 8 |
| American Journal of Industrial Medicine | 2022 | 65(10) | 7 |
| American Journal of Industrial Medicine | 2022 | 65(11) | 11 |
| American Journal of Industrial Medicine | 2022 | 65(12) | 10 |
| Work | 2015 | 50(1) | 18 |
| Work | 2015 | 50(2) | 18 |
| Work | 2015 | 50(3) | 24 |
| Work | 2015 | 50(4) | 21 |
| Work | 2015 | 51(1) | 18 |
| Work | 2015 | 51(2) | 27 |
| Work | 2015 | 51(3) | 22 |
| Work | 2015 | 51(4) | 29 |
| Work | 2015 | 52(1) | 21 |
| Work | 2015 | 52(2) | 23 |
| Work | 2015 | 52(3) | 30 |
| Work | 2015 | 52(4) | 26 |
| Work | 2016 | 53(1) | 23 |
| Work | 2016 | 53(2) | 25 |
| Work | 2016 | 53(3) | 22 |
| Work | 2016 | 53(4) | 25 |
| Work | 2016 | 54(1) | 25 |
| Work | 2016 | 54(2) | 24 |
| Work | 2016 | 54(3) | 27 |
| Work | 2016 | 54(4) | 24 |
| Work | 2016 | 55(1) | 25 |
| Work | 2016 | 55(2) | 25 |
| Work | 2016 | 55(3) | 24 |
| Work | 2016 | 55(4) | 23 |
| Work | 2017 | 56(1) | 18 |
| Work | 2017 | 56(2) | 19 |
| Work | 2017 | 56(3) | 16 |
| Work | 2017 | 56(4) | 15 |
| Work | 2017 | 57(1) | 15 |
| Work | 2017 | 57(2) | 16 |
| Work | 2017 | 57(3) | 14 |
| Work | 2017 | 57(4) | 18 |
| Work | 2017 | 58(1) | 11 |
| Work | 2017 | 58(2) | 16 |
| Work | 2017 | 58(3) | 14 |
| Work | 2017 | 58(4) | 18 |
| Work | 2018 | 59(1) | 16 |
| Work | 2018 | 59(2) | 13 |
| Work | 2018 | 59(3) | 14 |
| Work | 2018 | 59(4) | 13 |
| Work | 2018 | 60(1) | 17 |
| Work | 2018 | 60(2) | 16 |
| Work | 2018 | 60(3) | 15 |
| Work | 2018 | 60(4) | 16 |
| Work | 2018 | 61(1) | 15 |
| Work | 2018 | 61(2) | 15 |
| Work | 2018 | 61(3) | 15 |
| Work | 2018 | 61(4) | 14 |
| Work | 2019 | 62(1) | 18 |
| Work | 2019 | 62(2) | 19 |
| Work | 2019 | 62(3) | 16 |
| Work | 2019 | 62(4) | 12 |
| Work | 2019 | 63(1) | 14 |
| Work | 2019 | 63(2) | 16 |
| Work | 2019 | 63(3) | 17 |
| Work | 2019 | 63(4) | 15 |
| Work | 2019 | 64(1) | 15 |
| Work | 2019 | 64(2) | 19 |
| Work | 2019 | 64(3) | 26 |
| Work | 2019 | 64(4) | 23 |
| Work | 2020 | 65(1) | 25 |
| Work | 2020 | 65(2) | 27 |
| Work | 2020 | 65(3) | 23 |
| Work | 2020 | 65(4) | 20 |
| Work | 2020 | 66(1) | 25 |
| Work | 2020 | 66(2) | 25 |
| Work | 2020 | 66(3) | 23 |
| Work | 2020 | 66(4) | 24 |
| Work | 2020 | 67(1) | 27 |
| Work | 2020 | 67(2) | 25 |
| Work | 2020 | 67(3) | 25 |
| Work | 2020 | 67(4) | 27 |
| Work | 2021 | 68(s1) | 28 |
| Work | 2021 | 68(1) | 27 |
| Work | 2021 | 68(2) | 23 |
| Work | 2021 | 68(3) | 39 |
| Work | 2021 | 68(4) | 31 |
| Work | 2021 | 69(1) | 30 |
| Work | 2021 | 69(2) | 34 |
| Work | 2021 | 69(3) | 39 |
| Work | 2021 | 69(4) | 21 |
| Work | 2021 | 70(1) | 31 |
| Work | 2021 | 70(2) | 30 |
| Work | 2021 | 70(3) | 32 |
| Work | 2021 | 70(4) | 26 |
| Work | 2022 | 71(1) | 26 |
| Work | 2022 | 71(2) | 20 |
| Work | 2022 | 71(3) | 31 |
| Work | 2022 | 71(4) | 38 |
| Work | 2022 | 72(1) | 36 |
| Work | 2022 | 72(2) | 37 |
| Work | 2022 | 72(3) | 34 |
| Work | 2022 | 72(4) | 49 |
| Work | 2022 | 73(s1) | 23 |
| Work | 2022 | 73(1) | 31 |
| Work | 2022 | 73(2) | 35 |
| Work | 2022 | 73(3) | 31 |
| Work | 2022 | 73(4) | 31 |
| Safety Science | 2015 | 71, part A | 10 |
| Safety Science | 2015 | 71, part B | 13 |
| Safety Science | 2015 | 71, part C | 12 |
| Safety Science | 2015 | 72 | 42 |
| Safety Science | 2015 | 73 | 18 |
| Safety Science | 2015 | 74 | 22 |
| Safety Science | 2015 | 75 | 19 |
| Safety Science | 2015 | 76 | 23 |
| Safety Science | 2015 | 77 | 22 |
| Safety Science | 2015 | 78 | 22 |
| Safety Science | 2015 | 79 | 37 |
| Safety Science | 2015 | 80 | 34 |
| Safety Science | 2016 | 81 | 12 |
| Safety Science | 2016 | 82 | 47 |
| Safety Science | 2016 | 83 | 13 |
| Safety Science | 2016 | 84 | 26 |
| Safety Science | 2016 | 85 | 30 |
| Safety Science | 2016 | 86 | 27 |
| Safety Science | 2016 | 87 | 30 |
| Safety Science | 2016 | 88 | 27 |
| Safety Science | 2016 | 89 | 37 |
| Safety Science | 2016 | 90 | 11 |
| Safety Science | 2017 | 91 | 38 |
| Safety Science | 2017 | 92 | 38 |
| Safety Science | 2017 | 93 | 28 |
| Safety Science | 2017 | 94 | 21 |
| Safety Science | 2017 | 95 | 21 |
| Safety Science | 2017 | 96 | 22 |
| Safety Science | 2017 | 97 | 18 |
| Safety Science | 2017 | 98 | 19 |
| Safety Science | 2017 | 99, part A | 13 |
| Safety Science | 2017 | 99, part B | 14 |
| Safety Science | 2017 | 100, part A | 15 |
| Safety Science | 2017 | 100, part B | 11 |
| Safety Science | 2018 | 101 | 33 |
| Safety Science | 2018 | 102 | 26 |
| Safety Science | 2018 | 103 | 34 |
| Safety Science | 2018 | 104 | 26 |
| Safety Science | 2018 | 105 | 24 |
| Safety Science | 2018 | 106 | 30 |
| Safety Science | 2018 | 107 | 22 |
| Safety Science | 2018 | 108 | 29 |
| Safety Science | 2018 | 109 | 43 |
| Safety Science | 2018 | 110, part A | 43 |
| Safety Science | 2018 | 110, part B | 30 |
| Safety Science | 2018 | 110, part C | 16 |
| Safety Science | 2019 | 111 | 31 |
| Safety Science | 2019 | 112 | 24 |
| Safety Science | 2019 | 113 | 48 |
| Safety Science | 2019 | 114 | 17 |
| Safety Science | 2019 | 115 | 42 |
| Safety Science | 2019 | 116 | 28 |
| Safety Science | 2019 | 117 | 53 |
| Safety Science | 2019 | 118 | 89 |
| Safety Science | 2019 | 119 | 46 |
| Safety Science | 2019 | 120 | 93 |
| Safety Science | 2020 | 121 | 62 |
| Safety Science | 2020 | 122 | 33 |
| Safety Science | 2020 | 123 | 31 |
| Safety Science | 2020 | 124 | 28 |
| Safety Science | 2020 | 125 | 34 |
| Safety Science | 2020 | 126 | 22 |
| Safety Science | 2020 | 127 | 36 |
| Safety Science | 2020 | 128 | 38 |
| Safety Science | 2020 | 129 | 58 |
| Safety Science | 2020 | 130 | 50 |
| Safety Science | 2020 | 131 | 41 |
| Safety Science | 2020 | 132 | 31 |
| Safety Science | 2021 | 133 | 44 |
| Safety Science | 2021 | 134 | 43 |
| Safety Science | 2021 | 135 | 27 |
| Safety Science | 2021 | 136 | 32 |
| Safety Science | 2021 | 137 | 20 |
| Safety Science | 2021 | 138 | 29 |
| Safety Science | 2021 | 139 | 41 |
| Safety Science | 2021 | 140 | 32 |
| Safety Science | 2021 | 141 | 31 |
| Safety Science | 2021 | 142 | 40 |
| Safety Science | 2021 | 143 | 32 |
| Safety Science | 2021 | 144 | 29 |
| Safety Science | 2022 | 145 | 35 |
| Safety Science | 2022 | 146 | 41 |
| Safety Science | 2022 | 147 | 49 |
| Safety Science | 2022 | 148 | 25 |
| Safety Science | 2022 | 149 | 13 |
| Safety Science | 2022 | 150 | 21 |
| Safety Science | 2022 | 151 | 26 |
| Safety Science | 2022 | 152 | 33 |
| Safety Science | 2022 | 153 | 30 |
| Safety Science | 2022 | 154 | 29 |
| Safety Science | 2022 | 155 | 23 |
| Safety Science | 2022 | 156 | 12 |

## 4 Grey literature searches with results

We searched a comprehensive selection of grey literature resources using search terms covering the main aspects of our framework of interventions and outcomes. We performed both simple and advanced searches and used filters if relevant, depending on the functionalities available for each individual resource. In the table below, we document the grey searches performed, with number of hits listed for each search term/combination of terms on each resource and with a specification of search type and filters. In total, we screened approximately 19.573 grey literature references.

| Resource | Type of search | Search terms | Results |
| --- | --- | --- | --- |
| Social Science Research Network | Title/abstract & keywords | inspect | 344 |
|  | Searched in title in the Economics, Health Economics, Medical, Public Health, Social Insurance, Sociology networks | inspection | 80 |
|  | Searched in title in the Economics, Health Economics, Medical, Public Health, Social Insurance, Sociology networks, sorted by date and screened papers posted in the period 2020-2022 | regulation | 454 |
|  | Searched in title in the Economics, Health Economics, Medical, Public Health, Social Insurance, Sociology networks, last three years | compliance | 198 |
|  | Searched in title, abstract and keywords in the Economics, Health Economics, Medical, Public Health, Social Insurance, Sociology networks | Occupational Health and Safety | 190 |
|  | Searched in title in the Economics, Health Economics, Medical, Public Health, Social Insurance, Sociology networks, last three years | enforce | 6 |
|  | Searched in title in the Economics, Health Economics, Medical, Public Health, Social Insurance, Sociology networks, last three years | enforcement | 190 |
| NBER working paper series | Free text, sorted by relevance and screened the first 200 (of 4785) | inspection | 200 |
|  | Free text, sorted by relevance | regulation | 14.197 (first 200 screened) |
|  | Free text, filter working paper, sorted by date | regulation | 10.168 (first 200 screened) |
|  | Free text, filter working paper, sorted by date | enforcement | 6.564 (first 200 screened) |
|  | Free text, sorted by relevance | compliance | 3.550 (first 200 screened) |
|  | Working papers, searched in title | OSHA | 81 |
| National Institute for Occupational Safety and Health (NIOSH) | Searched in NIOSH Numbered Publications | regulation | 20 |
|  | Searched in NIOSH Numbered Publications | inspection | 39 |
|  | Searched in NIOSH Numbered Publications | enforcement | 78 |
|  | Searched in NIOSH Numbered Publications | enforce | 8 |
|  | Searched in NIOSH Numbered Publications | compliance | 66 |
| OECD iLibrary | Advanced search: from (Title contains ‘the search word’) AND from (IGO collection contains ‘“igo/oecd”’) with type(s) subtype/journal OR subtype/article OR subtype/workingpaperseries OR subtype/workingpaper | regulat* | 265 |
|  | Advanced search: from (Title contains ‘the search word’) AND from (IGO collection contains ‘“igo/oecd”’) with type(s) subtype/journal OR subtype/article OR subtype/workingpaperseries OR subtype/workingpaper | inspect* | 0 |
|  | Advanced search: from (Title contains ‘the search word’) AND from (IGO collection contains ‘“igo/oecd”’) with type(s) subtype/journal OR subtype/article OR subtype/workingpaperseries OR subtype/workingpaper | enforce* | 18 |
|  | Advanced search: from (Title contains ‘the search word’) AND from (IGO collection contains ‘“igo/oecd”’) with type(s) subtype/journal OR subtype/article OR subtype/workingpaperseries OR subtype/workingpaper | compliance | 4 |
|  | Advanced search: from (Title contains ‘the search word’) AND from (IGO collection contains ‘“igo/oecd”’) with type(s) subtype/journal OR subtype/article OR subtype/workingpaperseries OR subtype/workingpaper | comply | 0 |
|  | Advanced search: from (All Fields contains ‘occupational’) from (Language contains ‘en’) AND from (Title contains ‘safety’) AND from (IGO collection contains ‘“igo/oecd”’) with type(s) subtype/journal OR subtype/article OR subtype/workingpaperseries OR subtype/workingpaper | occupational in all fields and safety in title | 16 |
|  | Advanced search: from (All Fields contains ‘occupational’) from (Language contains ‘en’) AND from (Title contains ‘health’) AND from (IGO collection contains ‘“igo/oecd”’) with type(s) subtype/journal OR subtype/article OR subtype/workingpaperseries OR subtype/workingpaper | occupational in all fields and health in title | 80 |
| OpenGrey in DANS EASY Archive | Advanced search: ‘search word’ in title | inspection | 19 |
|  | Advanced search: ‘search word’ in title | inspect | 0 |
|  | Advanced search: ‘search word’ in title | regulation | 265 |
|  | Advanced search: ‘search word’ in title | enforce | 5 |
|  | Advanced search: ‘search word’ in title | enforcement | 15 |
|  | Advanced search: ‘search word’ in title | compliance | 47 |
|  | Advanced search: ‘search word’ in title | occupational health and safety | 0 |
|  | Advanced search: ‘search word’ in any field | occupational health and safety | 7 |
| International Labour Organization | Browsing by subject | Occupational safety and health | 70 |
|  | Browsing by subject | Education and training | 33 |
|  | Browsing by subject | Working conditions | 32 |
|  | Browsing by subject | Labour legislation | 12 |
|  | Browsing by subject | Labour law | 10 |
|  | Browsing by subject | Hazardous work | 9 |
|  | Browsing by subject | Work environment | 9 |
|  | Browsing by subject | International labour standards | 7 |
|  | Browsing by subject | Labour inspection | 7 |
|  | Browsing by subject | Occupational diseases | 5 |
|  | Browsing by subject | Workplace violence | 2 |
|  | Browsing by subject | Occupational injuries | 2 |
|  | Browsing by subject | Safety training | 2 |
|  | Browsing by subject | Occupational accidents | 1 |
|  | Browsing by subject | Occupational health services | 1 |
|  | Browsing by subject | Stress | 1 |
| Google Scholar | Sorted by relevance | regulatory intervention | First 100 hits |
|  | Sorted by relevance | regulatory intervention workplace | First 100 hits |
|  | Sorted by relevance | occupational health safety workplace | First 100 hits |
|  | Sorted by relevance | work occupational health safety inspection | First 100 hits |
| United States Department of Labor ‐ Occupational Safety and Health Administration (OSHA) | Searched in ‘Publications' | regulation | 2 |
|  | Searched in ‘Publications' | regulate | 0 |
|  | Searched in ‘Publications' | enforce | 0 |
|  | Searched in ‘Publications' | compliance | 12 |
|  | Searched in ‘Publications' | inspection | 8 |
| The European Agency for Safety and Health at Work (EU‐OSHA) | Screened all Discusion papers |  | 51 |
|  | Searched in ‘Reports' | regulation | 10 |
|  | Searched in ‘Reports' | regulate | 6 |
|  | Searched in ‘Reports' | enforce | 0 |
|  | Searched in ‘Reports' | compliance | 3 |
|  | Searched in ‘Reports' | inspection | 0 |
| Canadian Centre for Occupational Health and Safety | Screened all publications |  | 33 |
| The Health and Safety Executive (UK) | Search restricted to ‘Research' | inspections of the workplace hse | 8 |
|  | Search restricted to ‘Research' | enforcement hse | 7 |
| European Union Senior Labour Inspectors Committee | Searched in ‘Publications catalogue & document database' | regulation | 159 |
|  | Searched in ‘Publications catalogue & document database' | regulate | 18 |
|  | Searched in ‘Publications catalogue & document database' | enforce | 35 |
|  | Searched in ‘Publications catalogue & document database' | compliance | 22 |
|  | Searched in ‘Publications catalogue & document database' | inspection | 72 |
| European Foundation for the Improvement of Living and Working Conditions (Eurofound) | Searched in ‘Publications’. Filter: research report | regulation | 197 |
|  | Searched in ‘Publications’. Filter: research report | regulate | 81 |
|  | Searched in ‘Publications’. Filter: research report | enforce | 23 |
|  | Searched in ‘Publications’. Filter: research report | compliance | 73 |
|  | Searched in ‘Publications’. Filter: research report | inspection | 43 |
|  | Searched in ‘Publications’. Topic: Health and well-being at work. Filter: research report |  | 78 |
| Safe Work Australia | Search restricted to ‘Reports' | regulation | 3 |
|  | Search restricted to ‘Reports' | regulate | 2 |
|  | Search restricted to ‘Reports' | enforce | 0 |
|  | Search restricted to ‘Reports' | enforcement | 2 |
|  | Search restricted to ‘Reports' | compliance | 3 |
|  | Search restricted to ‘Reports' | inspection | 2 |
| WorkSafe (New Zealand) | Searched in publications and resources - restricted to reports | regulation | 71 |
|  | Searched in publications and resources - restricted to reports | regulate | 20 |
|  | Searched in publications and resources - restricted to reports | enforce | 11 |
|  | Searched in publications and resources - restricted to reports | enforcement | 23 |
|  | Searched in publications and resources - restricted to reports | compliance | 9 |
|  | Searched in publications and resources - restricted to reports | inspection | 21 |
| The Danish Working Environment Authority (Arbejdstilsynet) | Searched in only search bar available on the site | regulation | 11 |
|  | Searched in only search bar available on the site | regulering | 135 |
|  | Searched in only search bar available on the site | regulativer | 1 |
|  | Searched in only search bar available on the site | håndhæve | 18 |
|  | Searched in only search bar available on the site | håndhævelse | 20 |
|  | Searched in only search bar available on the site | Compliance | 11 |
|  | Searched in only search bar available on the site | overholdelse | 57 |
|  | Searched in only search bar available on the site | Inspection | 14 |
|  | Searched in only search bar available on the site | Inspektion | 43 |
| World Health Organisation—IRIS | Title search (using “advanced filters”) | Inspect (inspection) | 269 |
|  | Title search (using “advanced filters”) | Regulation | 171 (first 100 screened) |
|  | Title search (using “advanced filters”) | Compliance | 56 |
|  | Title search (using “advanced filters”) | Occupational Health and Safety | 18 |
|  | Title search (using “advanced filters”) | Enforce (enforcement) | 14 |
|  | Free text + filter by subject (using “advanced filters”) | Occupational Health and Safety | 97 |
|  | Free text + filter by subject (using “advanced filters”) | Enforce | 178 (first 100 screened) |
|  | Free text + filter by subject (using “advanced filters”) | Work environment | 18 |
|  | Free text + filter by subject (using “advanced filters”) | Compliance | 79 |
| EBSCO Open Dissertations (EBSCO‐host) (all databases) | Advanced Search - Abstract | occupational Health and Safety AND regulation | 2.264 (first 200 screened) |
|  | Advanced Search - Abstract | Inspection AND workplace | 658 (first 300 screened) |
|  | Advanced Search - Abstract | Enforcement AND occupation* | 2.489 (first 100 screened) |
|  | Advanced Search - Abstract | compliance OR adherence OR noncompliance OR non adherence AND occupation* AND health OR safety | 3.169 (first 100 screened) |
| Institute for Work & Health (Canada) | Simple search | Inspection | 47 |
|  | Simple search | regulation | 94 |
|  | Simple search | Enforcement | 52 |
|  | Simple search | Training initiatives | 103 |
| The National Institute of Occupational Health Norway (STAMI) | Searched in ‘Publications' | compliance | 108 |
|  | Searched in ‘Publications' | osha | 35 |
|  | Searched in ‘Publications' | arbeidsmiljø | 69 |
|  | Searched in ‘Publications' | arbeidsmiljømyndighetene | 2 |
|  | Searched in ‘Publications' | lovgivning | 1 |
|  | Searched in ‘Publications' | tilsyn | 11 |
|  | Searched in ‘Publications' | myndighetstiltak | 1 |
|  | Searched in ‘Publications' | arbeidstilsynet | 7 |
|  | Searched in ‘Publications' | tilsynsmyndigheten | 0 |
|  | Searched in ‘Publications' | håndhevelse | 1 |
|  | Searched in ‘Publications' | arbeidsmiljølovgivningen | 0 |
|  | Searched in ‘Publications' | helse | 115 |
|  | Searched in ‘Publications' | sikkerhet | 11 |
|  | Searched in ‘Publications' | inspection | 1 |
|  | Searched in ‘Publications' | regulate | 6 |
|  | Searched in ‘Publications' | regulation | 15 |
|  | Searched in ‘Publications' | enforce | 0 |
|  | Searched in ‘Publications' | enforcement | 0 |
|  | Searched in ‘Publications' | compliance | 0 |
| The Norwegian Labour Inspection Authority (Arbeidstillsynet) | Searched all ‘KOMPASS tema-rapporter' |  | 31 |
|  | Searched all ‘Forskningsrapporter bestilt av Arbeidstilsynet' |  | 7 |
|  | Searched all ‘Rapporter fra tilsynsprosjekter' |  | 27 |
| Best Evidence Encyclopedia | Searched in only search bar available on the site | inspection | 0 |
|  | Searched in only search bar available on the site | regulate | 0 |
|  | Searched in only search bar available on the site | regulation | 0 |
|  | Searched in only search bar available on the site | enforce | 0 |
|  | Searched in only search bar available on the site | enforcement | 0 |
|  | Searched in only search bar available on the site | compliance | 0 |
|  | Searched in only search bar available on the site | safety | 0 |
|  | Searched in only search bar available on the site | OSHA | 0 |
|  | Searched in only search bar available on the site | Occupational Health | 0 |
| ANU—The National Research Centre for OHS Regulation | searched in publications | inspection | 3 |
|  | searched in publications | regulation | 123 |
|  | searched in publications | enforcement | 15 |
|  | searched in publications | compliance | 22 |
|  | searched in publications | Occupational Health | 9 |
|  | searched in publications | Occupation | 11 |
| Finnish Institute of Occupational Health | Simple search | inspection | 5 |
|  | Simple search | regulation | 16 |
|  | Simple search | enforcement | 0 |
|  | Simple search | compliance | 4 |
|  | Simple search | training | 56 |
|  | Simple search | safety | 90 |
| 3ie Systematic Review Database | Site-wide search results | regulation AND occupation | 34 |
|  | Search in 3ie development evidence portal | regulation AND occupation | 0 |
|  | Search in 3ie development evidence portal | regulation | 276 |
|  | Search in 3ie development evidence portal | occupation | 38 |
|  | Search in 3ie development evidence portal | occupation | 28 |
|  | Search in 3ie development evidence portal | Occupational Health and Safety | 14 |
|  | Sitewide search results | Occupational Health and Safety | 469 (first 100 screened) |
|  | Sitewide search results | work environment | 750 (first 100 screened) |
|  | Sitewide search results | Labour legislation | 65 |
|  | Evidence hub --> 3ie’s Development Evidence Portal (Study type: Systematic Reviews + Evidence Gap map). Advanced search | “Occupational Health and Safety” | 10 |
|  | Evidence hub --> 3ie’s Development Evidence Portal (Study type: Systematic Reviews + Evidence Gap map). Advanced search | “Labour legislation” | 0 |
|  | Evidence hub --> 3ie’s Development Evidence Portal (Study type: Systematic Reviews + Evidence Gap map). Advanced search | regulation AND occupation | 0 |
|  | Evidence hub --> 3ie’s Development Evidence Portal (Study type: Systematic Reviews + Evidence Gap map). Advanced search | “regulatory intervention” | 1 |
|  | Evidence hub --> 3ie’s Development Evidence Portal (Study type: Systematic Reviews + Evidence Gap map). Advanced search | “safety training” | 0 |
|  | Evidence hub --> 3ie’s Development Evidence Portal (Study type: Systematic Reviews + Evidence Gap map). Advanced search | inspection | 10 |
| Evidence‐Based Synthesis Program (Department of Veteran Affairs) | published reports --> searched all ESP reports | Occupational Health and Safety | 0 |
|  | published reports --> searched all ESP reports | regulation | 2 |
|  | published reports --> searched all ESP reports | Labour legislation | 1 |
|  | published reports --> searched all ESP reports | inspection | 1 |
|  | published reports --> searched all ESP reports | enforcement | 0 |
|  | published reports --> searched all ESP reports | compliance | 4 |
|  | published reports --> searched all ESP reports | safety | 23 |
|  | published reports --> searched all ESP reports | work environment | 1 |
|  | published reports --> searched all ESP reports | occupation | 0 |
|  | published reports --> searched all ESP reports | work | 22 |
| Campbell Systematic Reviews Journal | Advanced search (anywhere) | “Occupational Health and Safety” | 13 |
|  | Advanced search (anywhere) | “Labour legislation” | 3 |
|  | Advanced search (anywhere) | regulation AND occupation | 60 |
|  | Advanced search (anywhere) | Enforcement AND occupation | 121 |
|  | Advanced search (anywhere) | inspection | 188 |
|  | Advanced search (anywhere) | Training initiatives AND occupation | 83 |
| Open Science Framework | Simple search with tags (meta-analysis AND systematic review) | Occupational Health and Safety | 5 |
|  | Simple search with tags (meta-analysis AND systematic review) | Inspection | 1 |
|  | Simple search with tags (meta-analysis AND systematic review) | enforcement | 0 |
|  | Simple search with tags (meta-analysis AND systematic review) | Labour legislation | 0 |
|  | Simple search with tags (meta-analysis AND systematic review) | legislation | 0 |
|  | Simple search with tags (meta-analysis AND systematic review) | Training initiatives | 8 |
|  | Simple search with tags (meta-analysis AND systematic review) | regulation | 7 |
|  | Simple search with tags (meta-analysis AND systematic review) | safety training | 15 |
|  | Simple search with tags (meta-analysis AND systematic review) | Work environment | 13 |
|  | Simple search with tags (meta-analysis AND systematic review) | Hazardous work | 161 |
|  | Simple search with tags (meta-analysis AND systematic review) | compliance | 0 |
|  | Simple search with tags (meta-analysis AND systematic review) | Occupation | 1 |
| Epistemonikos | standard search, filter: systematic review | Occupational Health and Safety | 50 |
|  | Advanced search (title/abstract) Filters --> Publication type: Systematisk Review | accident OR injury AND occupational | 282 (first 100 hits) |
|  | Advanced search (title/abstract) Filters --> Publication type: Systematisk Review | compliance AND occupational health | 27 |
|  | Advanced search (title/abstract) Filters --> Publication type: Systematisk Review | occupational health AND inspection | 4 |
|  | standard search, filter: systematic review | work environment | 116 |
|  | Advanced search (title/abstract) Filters --> Publication type: Systematisk Review | occupational health and safety AND regulation | 10 |
|  | Advanced search (title/abstract) Filters --> Publication type: Systematisk Review | OSH AND regulation | 2 |
|  | Advanced search (title/abstract) Filters --> Publication type: Systematisk Review | OSHA AND regulation | 1 |
| google.com | advanced search in Google search bar | Regulation work OR safety -china | 301.000.000 (first 41 pages) |
|  | advanced search in google search bar | Inspection AND regulation (work OR occupational) | 160.000.000 (first 23 pages) |
|  | normal google search bar | Occupational Health AND Safety | 782.000.000 (first 44 pages) |
|  | advanced search in google search bar | Enforcement AND Labor Legislation | 45.200.000 (first 40 pages) |
|  | normal google search bar | Occupational Health AND Compliance | 109.000.000 (first 10 pages) |
|  | normal google search bar | work environment AND regulation | 1.760.000.000 (first 10 pages) |
|  | normal google search bar | regulation AND sanction (work OR occupational) | 134.000.000 (first 10 pages) |
|  | normal google search bar | incentive for compliance AND regulation (work OR occupational) | 63.100.000 (first 10 pages) |
|  | normal google search bar | injury prevention AND accident prevention (work OR occupational) | 39.700.000 (first 10 pages) |
| Evidence based policing matrix | Research Programs --> Systematic reviews --> Projects | Searched all ‘Projects' | 25 |
|  | Research Programs --> Evidence-Based Policing --> Projects | Searched all ‘Projects' | 34 |
| Federal Institute for Occupational Safety and Health (BAuA) | Searched in ‘Publications' | inspection | 60 |
|  | Searched in ‘Publications' | enforce | 9 |
|  | Searched in ‘Publications' | enforcement | 30 |
|  | Searched in ‘Publications' | regulation | 244 (first 100 screened) |
|  | Searched in ‘Publications' | regulate | 35 |
|  | Searched in ‘Publications' | compliance | 108 |
| Cochrane Library | By topic: Health and safety at work |  | 118 |
|  | title/abstract & keyword | work environment | 73 |
|  | title/abstract & keyword | regulatory enforcement | 2 |
|  | title/abstract & keyword | work regulation | 56 |
|  | title/abstract & keyword | work inspection | 45 |
|  | title/abstract & keyword | osha | 1 |
|  | advanced search | occupational health AND sanction | 2 |
|  | advanced search | occupational health AND citation | 26 |
|  | advanced search | occupational health AND certification | 1 |
|  | advanced search | occupational safety and health AND enforcement | 2 |
|  | advanced search | occupational safety and health AND training | 8 |
|  | advanced search | occupational safety and health AND campaign | 2 |
|  | advanced search | occupational safety and health AND intervention | 28 |
| The Swedish Work Environment Authority (Arbetsmiljöverket) | Filter by information type: report, Swedish chosen | Filter by information type: report | 256 (first 100 hits) |
|  | Filter by information type: report, English chosen | Filter by information type: report | 252 |
| The Danish National Research Centre for the Working Environment  (NFA) | Free text search + filter by “documents” and “publications” | Regulation (da: regulering) | 34 |
|  | Free text search + filter by “documents” and “publications” | Inspection (da: inspektion) | 13 |
|  | Free text search + filter by “documents” and “publications” | Occupational injury (da: arbejdsskade) | 17 |
|  | Free text search + filter by “documents” and “research” | Occupational accident (da: arbejdsulykke) | 8 |
|  | Search by subject category in publications + filter by “scientific article” | Allergy (da: allergi) | 418 |
|  | Search by subject category in publications + filter by “scientific article” | Occupational injuries (da: arbejdsskader) | 5 |
|  | Search by subject category in publications + filter by “scientific article” | Occupational accidents (da: arbejdsulykker) | 116 |
|  | Search by subject category in publications + filter by “scientific article” | A healthy and safe worklife (da: et sundt og sikkkert arbejdsliv) | 53 |
|  | Search by subject category in publications + filter by “scientific article” | Interventions (da: interventioner) | 76 |
|  | Search by subject category in publications + filter by “scientific article” | Mental health (da: mentalt helbred) | 59 |
|  | Search by subject category in publications + filter by “scientific article” | Sickness absence (da: sygefravær) | 261 |
|  | Search by subject category in publications + filter by “report” | arbejdsevne | 9 |
|  | Search by subject category in publications + filter by “report” | allergi | 61 |
|  | Search by subject category in publications + filter by “report” | arbejdshygiejne | 0 |
|  | Search by subject category in publications + filter by “report” | arbejdsmiljøepidemiologi | 39 |
|  | Search by subject category in publications + filter by “report” | arbejdsskader | 3 |
|  | Search by subject category in publications + filter by “report” | arbejdsmiljø og sikkerhedskultur | 34 |
|  | Search by subject category in publications + filter by “report” | arbejdsulykker | 49 |
|  | Search by subject category in publications + filter by “report“Search by subject category in publications + filter by “report” | et sund og sikkert arbejdsliv | 22 |
|  | Search by subject category in publications + filter by “report” | fysisk belastning | 12 |
|  | Search by subject category in publications + filter by “report” | interventioner | 15 |
|  | Search by subject category in publications + filter by “report” | kemisk arbejdsmiljø | 142 |
|  | Search by subject category in publications + filter by “report” | mentalt helbred | 8 |
|  | Search by subject category in publications + filter by “report” | muskel- og skeletbesvær og fysisk arbejdsbelastning | 47 |
|  | Search by subject category in publications + filter by “report“Search by subject category in publications + filter by “report” | psykosocialt arbejdsmiljø | 93 |
|  | Search by subject category in publications + filter by “report” | sikkerhedsadfærd | 5 |
|  | Search by subject category in publications + filter by “report” | sikkerhedsklima | 2 |
|  | Search by subject category in publications + filter by “report” | sikkerhedskultur | 38 |
|  | Search by subject category in publications + filter by “report” | stillesidende arbejde | 19 |
|  | Search by subject category in publications + filter by “report” | støj | 7 |
|  | Search by subject category in publications + filter by “report“Search by subject category in publications + filter by “report” | sygefravær | 34 |
|  | Search by subject category in publications + filter by “report” | vold og trusler | 11 |
|  | Search by subject category in publications + filter by “Ph.d-thesis” | allergi | 10 |
|  | Search by subject category in publications + filter by “Ph.d-thesis” | arbejdsevne | 1 |
|  | Search by subject category in publications + filter by “Ph.d-thesis” | arbejdshygiejne | 0 |
|  | Search by subject category in publications + filter by “Ph.d-thesis” | arbejdsmiljøepidemiologi | 3 |
|  | Search by subject category in publications + filter by “Ph.d-thesis” | arbejdsskader | 1 |
|  | Search by subject category in publications + filter by “Ph.d-thesis” | arbejdsmiljø og sikkerhedskultur | 6 |
|  | Search by subject category in publications + filter by “Ph.d-thesis” | arbejdsulykker | 10 |
|  | Search by subject category in publications + filter by “Ph.d-thesis” | et sund og sikkert arbejdsliv | 0 |
|  | Search by subject category in publications + filter by “Ph.d-thesis” | fysisk belastning | 1 |
|  | Search by subject category in publications + filter by “Ph.d-thesis” | interventioner | 3 |
|  | Search by subject category in publications + filter by “Ph.d-thesis” | kemisk arbejdsmiljø | 27 |
|  | Search by subject category in publications + filter by “Ph.d-thesis” | mentalt helbred | 1 |
|  | Search by subject category in publications + filter by “Ph.d-thesis” | muskel- og skeletbesvær og fysisk arbejdsbelastning | 12 |
|  | Search by subject category in publications + filter by “Ph.d-thesis” | psykosocialt arbejdsmiljø | 4 |
|  | Search by subject category in publications + filter by “Ph.d-thesis” | sikkerhedsadfærd | 1 |
|  | Search by subject category in publications + filter by “Ph.d-thesis” | sikkerhedsklima | 0 |
|  | Search by subject category in publications + filter by “Ph.d-thesis” | sikkerhedskultur | 6 |
|  | Search by subject category in publications + filter by “Ph.d-thesis” | stillesidende arbejde | 1 |
|  | Search by subject category in publications + filter by “Ph.d-thesis” | støj | 0 |
|  | Search by subject category in publications + filter by “Ph.d-thesis” | sygefravær | 9 |
|  | Search by subject category in publications + filter by “Ph.d-thesis” | vold og trusler | 2 |
| PROSPERO | standard search | occupational safety and health | 106 |
|  | standard search | work environment | 244 |
|  | standard search | OSHA | 22 |
|  | standard search | labour legislation | 1 |
|  | standard search | workplace regulation | 1 |
|  | standard search | safety at work | 5 |
|  | standard search | health at work | 21 |
|  | standard search | labour inspection | 3 |
|  | standard search | labor inspection | 1 |
|  | standard search | safety training | 16 |
|  | standard search | regulation compliance | 3 |
|  | standard search | regulatory compliance | 4 |
|  | standard search | incentive | 325 |
|  | standard search | sanction | 11 |
|  | standard search | enforcement | 195 |
|  | standard search | occupational injury | 24 |
|  | standard search | occupational accident | 8 |
| EPPI‐Centre publications | Searched all ‘Publications --> research reports' |  | 5 |
|  | Searched through ‘Publications --> Index of Knowledge’ for relevant topics. The references in the following topics were examined further: Employment, Workplace health, Incentive schemes, Accidental injury and Smoking |  | 15 |
| CORE | Advanced search | (work* or occupation*) and (safety or health or “working environment” or “work environment”) and (regulation* or regulatory or enforce* or intervention* or legislations* or citation or inspection*) | 225 (first 100 hits) |
|  | standard search | osha | 40.839 (first 100 hits) |
| AEA Social Science RCT Registry | standard search | occupational safety and health | 9 |
|  | standard search | work environment | 162 |
|  | standard search | labor inspection | 6 |
|  | standard search | labour legislation | 1 |
|  | standard search | labor regulation | 34 |
|  | standard search | regulation compliance | 18 |
|  | standard search | compliance incentive | 51 |
|  | standard search | sanction | 23 |
|  | standard search | regulatory enforcement | 9 |
|  | standard search | occupational injury | 2 |
|  | standard search | occupational accident | 2 |
|  | standard search | health at work | 455 (first 10 pages) |
|  | standard search | safety at work | 78 |
|  | standard search | work inspection | 6 |
|  | standard search | sickness absence | 6 |
| ClinicalTrials.gov | By topic: Occupational Diseases - 16 conditions listed, all screened |  | 1.112 (first 100 hits pr. condition) |
|  | standard search | work environment | 1.134 (first 100 hits) |
|  | standard search | occupational safety and health | 73 |
|  | standard search | work regulation | 1.328 (first 100 hits) |
|  | standard search | work inspection | 8.047 (first 100 hits) |
|  | standard search | compliance incentive | 104 |
|  | standard search | sanction | 31 |
|  | standard search | work injury | 3.537 (first 100 hits) |
|  | standard search | work accident | 211 (first 100 hits) |
|  | standard search | sickness absence | 540 |
| CENTRAL Trials Register within the Cochrane Library | title/abstract/keyword (trials tab chosen) | occupational health and safety | 684 (first 200 hits) |
|  | title/abstract/keyword (trials tab chosen) | occupational injury | 1.813 (first 100 hits) |
|  | title/abstract/keyword (trials tab chosen) | occupational accident | 699 (first 100 hits) |
|  | title/abstract/keyword (trials tab chosen) | work environment | 3.393 (first 100 hits) |
|  | title/abstract/keyword (trials tab chosen) | work inspection | 278 (first 100 hits) |
|  | title/abstract/keyword (trials tab chosen) | work citation | 105 |
|  | title/abstract/keyword (trials tab chosen) | OSHA | 36 |
|  | title/abstract/keyword (trials tab chosen) | OSH regulation | 1 |
|  | title/abstract/keyword (trials tab chosen) | OSHA regulation | 6 |
|  | title/abstract/keyword (trials tab chosen) | OSH enforcement | 2 |
|  | title/abstract/keyword (trials tab chosen) | work safety | 7.442 (first 100 hits) |
|  | title/abstract/keyword (trials tab chosen) | work health | 24.941(first 100 hits) |
| WHO International Clinical Trials Registry Platform | standard search | occupational safety and health | 91 |
|  | standard search | work inspection | 10 |
|  | standard search | work environment | 241 (first 100 hits) |
|  | standard search | OSHA | 5 |
|  | standard search | OSH | 2 |
|  | standard search | occupational injury | 764 (first 100 hits) |
|  | standard search | occupational accident | 28 |
|  | standard search | work accident | 19 |
|  | standard search | safety regulation | 57 |

## 5 Citation-tracking with results

We performed citation-tracking on all included studies, that is, the six systematic reviews, the 28 primary studies, and the three on-going studies. For the included systematic reviews, we also conducted forwards citation-tracking through Google Scholar and Web of Science to identify potentially relevant references that had cited these reviews.

In addition to citation-tracking the included references, we tracked the reference lists of a number of other reviews, research overviews, and primary studies identified during the search process that we judged to potentially contain relevant references.

A table documenting the citation-tracking proces of included and additional studies is provided below, with number of references screened for each study. In total, we screened 2.641 hits during the citation-tracking process.

| **Citation-tracking of included reviews** | **References screened** |
| --- | --- |
| Mischke (2013) | 17 |
| Andersen (2019) | 86 |
| Dyreborg (2022) | 32 |
| Rautiainen (2008) | 65 |
| Cashman (2009) | 40 |
| van der Molen (2018) | 91 |
|  |  |
| **Forwards citation-tracking of included reviews** | **References screened** |
| Mischke (2013) | Google Scholar: 92 |
|  | Web of Science: 29 |
| Andersen (2019) | 59 (Google Scholar and Web of Science combined) |
| Dyreborg (2022) | Google Scholar: 1 |
|  | Web of Science: 0 |
| Rautiainen (2008) | Google Scholar: 140 |
|  | Web of Science: 65 |
| Cashman (2009) | Google Scholar: 84 |
|  | Web of Science: 33 |
| van der Molen (2018) | Google Scholar: 100 |
|  | Web of Science: 23 |
|  |  |
| **Citation-tracking of included primary studies** | **References screened** |
| McLeod (2019) | 20 |
| Agnesi (2016) | 21 |
| Alwall (2020) | 46 |
| Chen (2008) | 10 |
| Dahl (2022) | 47 |
| Foley (2012) | 26 |
| Hogg-Johnson (2012) | 39 |
| Kemmlert (1996) | 124 |
| Kim (2021) | 53 |
| Johnson (2019) | 75 |
| López-Ruiz (2013) | 23 |
| McCaffrey (1983) | 17 |
| Moran (1985) | 21 |
| Nelson (1997) | 10 |
| Rubio-Romero (2015) | 40 |
| Peto (2016) | 32 |
| Ruser (1991) | 11 |
| Smith (1979) | 16 |
| Soriano-Serrano (2020) | 33 |
| Weil (1996) | 35 |
| Wickizer (2004) | 26 |
| Lewchuk (1996) | 26 |
| Stocks (2015) | 30 |
| Macpherson (2022) | 10 |
| Mancini (2005) | 17 |
| Dahl (2013) | 49 |
| McLeod (2018) | 18 |
| Björkdahl (2008) | 19 |
|  |  |
| **Citation-tracking of included on-going studies** | **References screened** |
| Abildgaard (2021) | no reference list |
| Indregard (2019) | 47 |
| Sundstrup (2022) | no reference list |
|  |  |
| **Citation-tracking of additional studies** | **References screened** |
| Kemmlert 1994 (included as secondary reference to Kemmlert (1996)) | 19 |
| Levine 2012 (included as secondary reference to Johnson (2019)) | 25 |
| Safe Work Australia (2013) | 43 |
| Johannessen (2017) | 152 |
| Salguero-Caparrós (2020) | 67 |
| Teufer (2019) | 51 |
| Tompa (2016) | 58 |
| Tompa (2007) | 47 |
| Verbeek (2013) | 47 |
| Arbetsmiljøverket (2016) | 124 |
| Haby (2016) | 3 |
| Robson (2007) | 80 |
| Clearinghouse for Labor Evaluation and Research | 27 |

## 6 First and second level screening

Three basic questions guided the screening which was performed in duplicate. First level screening was performed on the basis of titles and abstracts. Second level screening was on the basis of full texts. A study was excluded if one or more of the answers to questions 1-3 shown below were ‘No’ and there was agreement about this between the two screeners. If the answers to questions 1 to 3 were ‘Yes’ or ‘Uncertain’, the study was retrived in full text in order to assess second level eligibility. All unanswered questions were posed again on the basis of the full text.

**Screening questions:**

*1. Does the study measure the effects of eligible working environment regulatory interventions, as specified in the protocol?* Yes -include

No – stop here and exclude

Uncertain – include

Question 1 guidance: We only include interventions initiated by working environment regulatory authorities or agencies, thus excluding interventions started by individual businesses or employers at their own initiative. The following categories of intervention are eligible: formulation of regulatory standards, incentives for compliance, inspection by regulatory agencies, enforcement by regulatory agencies (sanctions), information, guidance, and consulting, and finally training initiatives. Please refer to the protocol for examples. If in doubt, include for second level screening on full text. *2. Does the study measure effects for workers above the age of 15 from workplaces within the OECD?* Yes – include

No – stop here and exclude

Uncertain – include Question 2 guidance: The population of relevance to this EGM includes workers above the age of 15 and their workplaces. We limit our scope to workplaces located in nations within the OECD. Note here that it is the workplaces that must be located in OECD countries, whereas workers in these workplaces may be citizens of all countries.

*3. Is the report/article a primary quantitative study with a control or comparison condition or a systematic review of effectiveness studies?* Yes – include

No – stop here and exclude

Uncertain – include

Question 3 guidance: We are only interested in primary quantitative studies with a control or comparison group or systematic reviews of effectiveness studies. For specification of included designs, see the section entitled “Types of study designs” in the protocol.

## 7 Data extraction

Data extraction was performed in EPPI-Reviewer using a codeset designed to accommodate the creation of the evidence and gap map in EPPI-Mapper. The codeset included clickable options with free-text fields supplied in order to capture all relevant details surrounding the publication itself, the study design, intervention and outcome domains, and population details (information about sample sizes if applicable, workplace location, and type of work/industry), as shown below:

- Publication type
  - Journal article
  - Dissertation
  - Report
  - Other
- Publication language
  - English
  - Danish
  - Norwegian
  - Swedish
  - Other
- Publication status
  - Completed
  - Ongoing
- Study design
  - Randomised controlled trial (RCT)
  - Non-randomised study (NRS)
  - Systematic review (SR) (high AMSTAR-rating)
  - Systematic review (SR) (moderate AMSTAR-rating)
  - Systematic review (SR) (low AMSTAR-rating)
  - Systematic review (SR) (critically low AMSTAR-rating)
  - Systematic review (SR) (no AMSTAR-rating)
- Type of regulatory intervention
  - Formulation of regulatory standards
  - Incentives for compliance
  - Inspection by regulatory agency
  - Enforcement by regulatory agency (sanctions)
  - Information, guidance, and consulting activity
  - Training initiative
- Intervention initiator
  - National regulatory authority
  - International regulatory authority
  - Other organisation authorised as regulator
- Outcomes (intermediate and final)
  - Compliance with regulation or regulatory action
  - Work-related exposure (e.g. to chemicals)
  - Incidence of work-related injuries
  - Health
  - Sickness absence
- Other outcome measures (free-text specification)
- Level of outcome
  - Organizational/workplace level
  - Individual/worker level
- Population details
  - Intervention group characteristics
    - Age
    - Socio-economic status (SES)
    - Gender
    - Sample size
  - Control group characteristics
    - Age
    - Socio-economic status (SES)
    - Gender
    - Sample size
- Workplace location (OECD country)
  - Portugal
  - Poland
  - Norway
  - Japan
  - The United States
  - The United Kingdom
  - Turkey
  - Switzerland
  - Sweden
  - Spain
  - Slovenia
  - Slovak Republic
  - New Zealand
  - The Netherlands
  - Mexico
  - Luxembourg
  - Lithuania
  - Latvia
  - Korea
  - Italy
  - Israel
  - Ireland
  - Iceland
  - Germany
  - France
  - Greece
  - Hungary
  - Finland
  - Estonia
  - Denmark
  - Austria
  - Costa Rica
  - Czech Republic
  - Colombia
  - Chile
  - Canada
  - Belgium
  - Australia
- Type of work/industry
  - Construction
  - Manufacturing
  - Mining
  - Agriculture, Forestry, and Fishing
  - Transportation and related services
  - Wholesale and retail trade
  - Accommodation and food service activities
  - Professional scientific and technological services
  - Human health and social work activities
  - Administrative and support service activities
  - Other/Not specified
- Comments

## 8 Link to online interactive EGM

Add link at the full report stage

## 9 AMSTAR-assessment of included systematic reviews

Sent as Excel-file to editor
